# Modeling control and transduction of electrochemical gradients in acid-stressed bacteria

**DOI:** 10.1016/j.isci.2023.107140

**Published:** 2023-06-17

**Authors:** Marcus S. Benyamin, Matthew P. Perisin, Caleb A. Hellman, Nathan D. Schwalm, Justin P. Jahnke, Christian J. Sund

**Affiliations:** 1 Biological and Biotechnology Sciences Division, DEVCOM Army Research Laboratory, Adelphi, MD, USA

**Keywords:** Electrochemistry, Microbial physiology, Microbiology

## Abstract

Transmembrane electrochemical gradients drive solute uptake and constitute a substantial fraction of the cellular energy pool in bacteria. These gradients act not only as “homeostatic contributors,” but also play a dynamic and keystone role in several bacterial functions, including sensing, stress response, and metabolism. At the system level, multiple gradients interact with ion transporters and bacterial behavior in a complex, rapid, and emergent manner; consequently, experiments alone cannot untangle their interdependencies. Electrochemical gradient modeling provides a general framework to understand these interactions and their underlying mechanisms. We quantify the generation, maintenance, and interactions of electrical, proton, and potassium potential gradients under lactic acid-stress and lactic acid fermentation. Further, we elucidate a gradient-mediated mechanism for intracellular pH sensing and stress response. We demonstrate that this gradient model can yield insights on the energetic limitations of membrane transport, and can predict bacterial behavior across changing environments.

## Highlights

• Ion transporters interact emergently through shared electrochemical gradients
• pH-gated KcsA imparts pH sensing to F-ATPase, ultimately controlling cytosolic pH
• The pH sensing is mediated by Δψ, which thermodynamically controls F-ATPase activity
• Sensitive to ion flux, Δψ rapidly and efficiently responds to environmental changes

## Graphical abstract

**Figure:**
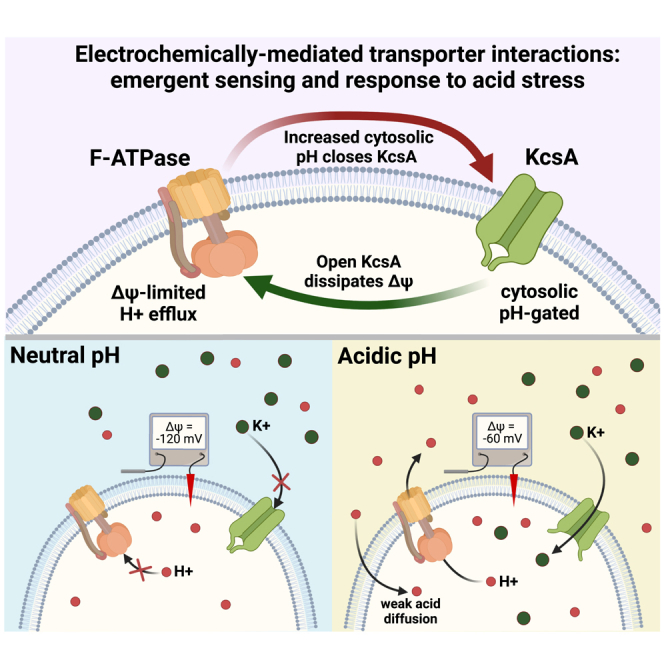

## Introduction

Transmembrane electrochemical gradients act as rapid-response mediators between membrane transport, signaling, homeostasis, and metabolism in bacteria.^1^^,^^2^^,^^3^ These gradients are maintained by a network of membrane-bound transporters, which together interconvert between ATP, the electrical gradient, and multiple ionic gradients.^4^^,^^5^ This “transportome” consumes a large portion of total cellular maintenance cost (as much as 60%), but it enables more flexible and efficient transport than ATP-driven transport alone.^6^ Membrane transporters frequently rely on the coupling of gradients in transport, where electrical and ionic gradients are consumed to drive otherwise unfavorable solute uptake or efflux. In addition to providing flexibility, such couplings enable survival in extreme environments. For example, acidophiles hold the electrical potential Δψ to be inside-positive, enabling $H^{+}$ removal even at an external pH of 2 or below.^4^ In this manner, electrochemical gradients provide cellular energy pools for nutrient uptake, maintenance of homeostasis, and stress response.^7^^,^^8^ However, these gradients are challenging to study at the system level, as they interact via membrane transporters that both maintain the gradients and rely on them to drive transport. To understand membrane transport at a system level, the complex interplay between electrochemical gradients, transporter expression, extracellular conditions, and bacterial phenotype must be untangled.^1^

The complex interactions and rapid dynamics of transport-gradient couplings have challenged experimental study at the system level. Complex interactions arise because a single transport system may influence multiple gradients, which in turn control the directionality and kinetics of many different transporters.^9^^,^^10^^,^^11^ For example, the electrical gradient Δψ arises from (and therefore influences) any net transport of charge across the cell membrane, including ion transport. These interactions are further confounded by rapid gradient dynamics,^12^^,^^13^^,^^14^ to which the electrical gradient is particularly sensitive. A bacterial cell membrane is fully polarized to −200 mV by the movement of $10^{5}$ ions across the cell membrane,^15^^,^^16^ corresponding to only a ∼ 40 *μ*M change in intracellular ion concentration. To untangle some of these interdependencies, ionophores are used to selectively extinguish ionic or electrical gradients in cells,^17^^,^^18^^,^^19^ and fluorescent compounds are used to passively measure electrochemical potentials during cell growth^14^^,^^20^ or in response to environmental changes.^13^^,^^21^^,^^22^ These methods establish correlations between electrochemical gradients and extracellular/intracellular conditions, elucidate gradient interactions, or determine the influence of an electrochemical gradient on overall cellular function. However, these experiments typically yield insights that are limited to a single gradient interaction, organism, and set of experimental conditions. Electrochemical gradients are likely to display emergent interactions, which cannot be elucidated through experiments on single gradient components.^11^^,^^22^^,^^23^^,^^24^^,^^25^^,^^26^^,^^27^^,^^28^^,^^29^^,^^30^ At the system level, the possible combinations of such interactions far exceed the capacity to identify them experimentally.

As a complement to experimentation, computational modeling can identify emergent interactions between electrochemical gradients, membrane transporters, and bacterial functions, such as metabolism and stress response. Although individual transport-gradient interactions have been experimentally investigated, the primary advantage of a computational approach is that it can model multiple gradients, transport systems, and interactions in tandem. At the single-process level, transport-gradient interactions are mathematically well-described and generalizable between systems. Ion transport forms electrochemical gradients across the cell membrane, which acts like a capacitor; the formulae for this approach have been extensively described,^1^^,^^16^ and most famously modeled by Hodgkin and Huxley,^31^ who related electrical potential to ionic currents. Conversely, electrochemical gradients are known to impose thermodynamic control on ion transport, and to exert additional gating control specific to a transport system.^32^^,^^33^ By combining models of individual ion transport systems, complex interactions that involve multiple transporters and gradients can be elucidated, and these interactions can be generalized between species with conserved mechanisms of ion transport. Integrated into a larger metabolic model, the interactions of transport systems, gradients, and bacterial behavior can then be predicted for specific organisms. Other bacterial models have empirically related stress response, metabolism, and growth to intracellular conditions, such as internal pH, solute concentrations, and metabolite concentrations.^34^^,^^35^^,^^36^ These models are supported by experimental data on cell stress, such as the deleterious effects of low pH and weak acid accumulation, and the minimum internal pH for growth and metabolism.^19^^,^^21^^,^^37^ Here, we focus on ion transport as a key stress-response mechanism to capture the influence of ion transport and electrochemical gradients on metabolism. With a mechanistic model of cell stress response, it is possible to model emergent interactions between transporters, gradients, and metabolism in a bacterial system.

One of the simplest systems with emergent behavior between ion transport, electrochemical gradients, and metabolism is the acid-stress response in lactic acid bacteria, which is mediated by the Δψ, ΔpH, and $\Delta \left[K^{+}\right]$ gradients. Δψ, ΔpH, and $\Delta \left[K^{+}\right]$ mediate energy conversion and storage,^13^^,^^38^ environmental signaling,^22^^,^^39^ stress response,^40^^,^^41^ and metabolic shifts^42^^,^^43^ across many prokaryotes. These gradients predominate in non-saline environments, and provide a simple system for elucidating gradient-based interactions. Under acid-stress conditions, the $F_{1}F_{0}$-ATPase (F-ATPase) pump maintains cytoplasmic pH via efflux of $H^{+}$ from the cytosol. This efflux generates a proton-motive force (PMF), composed of both Δψ and ΔpH. Because even small amounts of net charge transport cause large changes in Δψ, Δψ must be converted or $H^{+}$ efflux will cease. This conversion is accomplished by $K^{+}$ influx, which depolarizes the cell membrane and facilitates the interchange of Δψ to ΔpH.^13^^,^^44^ Experiments have demonstrated that $K^{+}$ influx is necessary for cytoplasmic pH maintenance and survival under acid stress,^17^^,^^40^ and that this influx must be gated to prevent total membrane depolarization or over-alkalinization of the cytosol.^45^ Rapid $K^{+}$ influx occurs through $K^{+}$ channels, such as KcsA, which is gated by both electrochemical potential^46^^,^^47^ and intracellular pH.^48^^,^^49^ The controlled manner of $K^{+}$ influx, and the necessity of $K^{+}$ influx for F-ATPase activity under acid stress, suggests that two-way control exists between F-ATPase and KcsA, and that this control is mediated by emergent interactions between Δψ, ΔpH, and $\Delta \left[K^{+}\right]$.

In this work, we present a case study of emergent interactions between electrochemical gradients. Specifically, we model the interplay of the electrical, $H^{+}$, and $K^{+}$ gradients under acid stress and acid fermentation, where bacteria must maintain a ΔpH to survive. We model homolactic fermentation by lactic acid bacteria, as their metabolism has well-studied and well-defined ATP and product yields,^50^ and allows us to study the acid stress conditions generated by fermentation. In addition, this fermentative metabolism allows us to exclude the consumption of Δψ by the electron transport chain, which would need to be considered in a model of respiration. We model $H^{+}$ and $K^{+}$ transport in cases of increasing complexity: we first consider $H^{+}$ transport in isolation, then couple it with $K^{+}$ transport, and finally combine both with a simplified metabolic model. In the first two model cases, $H^{+}$ enters the cell by diffusion of lactic acid and is excluded by the activity of F-ATPase. In the case of coupled $H^{+}$ and $K^{+}$ transport, Δψ is converted to $\Delta \left[K^{+}\right]$ by KcsA. Using a system of ordinary differential equations (ODEs), we model cytosolic pH response to acid stress where Δψ is constant ($H^{+}$ current only) or dynamic ($H^{+}$ and $K^{+}$ currents). Here, we capture energetic limitations and tradeoffs of cells under acid-stress, and demonstrate how Δψ mediates pH-control of the F-ATPase through KcsA. Finally, we model ionic transport in concert with metabolism in a Monod-based acid fermentation model, and estimate the amount and “quality” of energy stored in each gradient. As a result, we can model organism robustness and explore strategies to ameliorate or aggravate cell stress by changing culture conditions, or by engineering changes in membrane proteins with respect to expression level, affinities, and activation/deactivation. With this model, we provide a framework to identify gradient-transport-function interactions for experimental investigation, and to inform organism engineering strategies for enhanced survival.

## Results

### Inherent limits of F-ATPase pH response under acid stress

The $F_{1}F_{0}$-ATPase (F-ATPase) consumes ATP to efflux $H^{+}$ and generate a proton-motive force (PMF) consisting of Δψ and ΔpH.^51^ Efflux through F-ATPase comprises a key part of the acid-stress response,^52^^,^^53^^,^^54^ as evidenced by the necessity of F-ATPase for survival at low pH^55^ and the increased expression of F-ATPase with decreasing pH.^56^ However, PMF generation by F-ATPase becomes thermodynamically limited as the work required for $H^{+}$ efflux approaches the free energy of ATP hydrolysis. The amount of work required is determined by Δψ, ΔpH, and the number of $H^{+}$ transported per ATP (*n*). The effects of these limitations have been demonstrated experimentally; for example, bacteria cannot simultaneously maintain a large Δψ and a large ΔpH, particularly in the presence of a permeating weak acid.^57^ Accordingly, we expect that Δψ and the $H^{+}$/ATP ratio *n* will limit the steady-state intracellular pH under acid-stress conditions.

**Figure 1:**
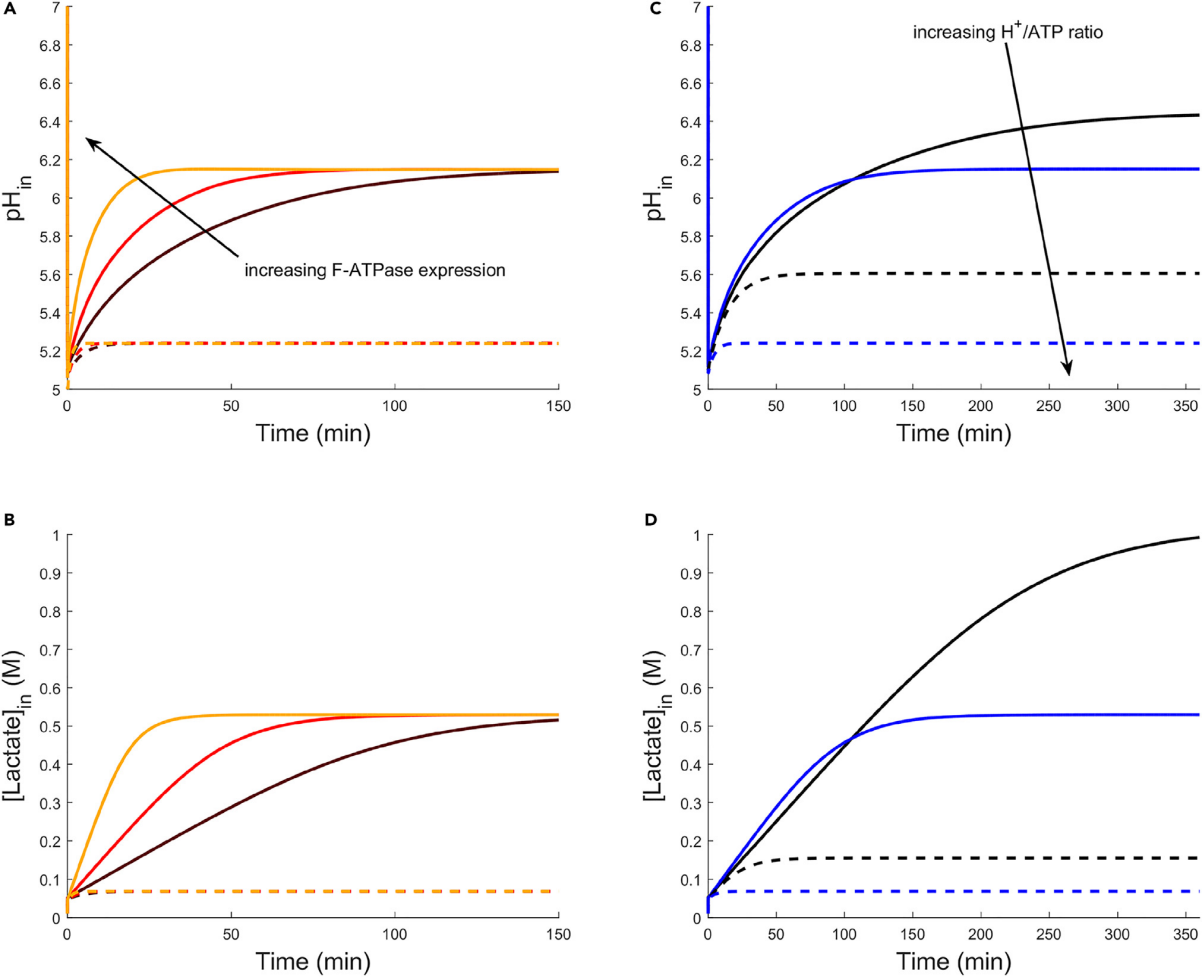
Cytosolic pH recovery driven by F-ATPase activity Dynamics of pH recovery and internal lactate accumulation under lactic acid shock at fixed membrane potential (Δψ). (A) Cytosolic pH recovery for varied F-ATPase expression level. F-ATPase expression level is set to 1x (brown), 2x (red), or 5x (gold) expression, and membrane potential is fixed at −40 mV (solid lines) or −100 mV (dashed lines). Initial cytosolic pH is 7.0, and external acid-stress is 50 mM lactate at an extracellular pH of 5.0. (B) Internal lactate accumulation corresponding to (A). Initial internal lactate concentration is 10 mM. (C) Cytosolic pH recovery for varied $H^{+}$/ATP ratio. $H^{+}$/ATP ratio is set to 3.3 (black) or 4.0 (blue), and membrane potential is fixed at −40 mV (solid lines) or −100 mV (dashed lines). Initial cytosolic pH is 7.0, and external acid-stress is 50 mM lactate at an extracellular pH of 5.0. (D) Internal lactate accumulation corresponding to (C). Initial internal lactate concentration is 10 mM.

To test whether our model captures these limitations, we modeled $H^{+}$ transport in response to weak acid stress, and calculated the dynamics of the cytosolic pH response (Figure 1) using a system of ordinary differential equations. Here, we considered the transport of $H^{+}$ into the cell by passive diffusion of lactic acid, and the removal of $H^{+}$ by F-ATPase. The rate of $H^{+}$ efflux by F-ATPase depends on substrate and product concentrations (ATP, ADP, and Pi) and the PMF.^58^^,^^59^ To isolate ΔpH formation, we held constant the concentrations of cytosolic ATP, ADP, and Pi, and fixed Δψ at a constant value of −100 mV (dashed lines) or −40 mV (solid lines). We chose these Δψ values according to the polarized (large Δψ) and depolarized (small Δψ) membrane states of lactic acid bacteria under acid stress.^44^ To model F-ATPase activity, we developed a kinetic expression that depends on Δψ, ΔpH, and *n*, based on literature data and mechanism.^59^^,^^60^ We then considered the impact on steady-state pH of increased F-ATPase expression, which occurs during acid stress, and of changes in *n*, which differs between species and presents a potential target for genetic engineering.^61^^,^^62^

We first modeled the dynamics of cytosolic pH recovery (Figure 1A) and internal lactate concentration (Figure 1B) for varied F-ATPase expression level, with expression held at 1x (brown), 2x (red), or 5x (gold). In Figure 1A, the influx of lactic acid causes a rapid decrease in internal pH (initially 7.0). Dissociated lactate remained in the cytosol (1B), and the concentrations were consistent with the expected partition of lactate anion, given the pH difference. As F-ATPase pumped $H^{+}$ from the cytosol, the pH partially recovered in all cases. Although the steady-state pH depended strongly on Δψ, it exhibited no dependence on F-ATPase expression level. Rather, the only benefit of increased F-ATPase expression level was an improvement in pH recovery dynamics, and only at small Δψ. Although the expression level impacted dynamics, it did not shift equilibrium; for a given Δψ, the steady-state internal pH and internal lactate concentration both remained the same.

In contrast to F-ATPase expression level, the $H^{+}$/ATP ratio *n* controls the thermodynamics of $H^{+}$ efflux, as the work required for efflux is directly proportional to *n*. This ratio is determined by the stoichiometry of the $F_{0}$ c-ring, which can be modified by genetic engineering to produce a chimeric $F_{1}F_{0}$–ATPase with a different number of c-subunits.^62^^,^^63^ To study how *n* influences the dynamics of cytosolic pH recovery, we repeated the simulation, but held the F-ATPase expression level constant, and instead varied *n* (Figures 1C and 1D). As with the prior simulation, the cytosolic pH in 1C dropped sharply from 7.0, before partially recovering to a steady-state value (Figure 1C). Likewise, the cytosolic lactate concentration in 1D was consistent with the expected partition (Figure 1D). Unlike changing the F-ATPase expression level, increasing the $H^{+}$/ATP ratio caused directionally opposite shifts in recovery dynamics and steady-state cytosolic pH. At small Δψ, increased *n* produced a modest, transient improvement in pH recovery dynamics, because of an increased number of $H^{+}$ ions transported per ATP hydrolyzed and similar initial rates of ATP hydrolysis. However, the steady-state pH was lower (0.3 pH units), as the work required for proton efflux was directly proportional to both the ratio *n* and the PMF.^59^ At large Δψ, the increased ratio *n* causes a similar reduction in steady state pH (0.35 pH units), but with no apparent improvement in recovery dynamics (Figure 1C). These results yielded insights on the energetic tradeoffs of the $H^{+}$/ATP ratio in the F-ATPase acid-stress response system. At high $H^{+}$/ATP ratio, F-ATPase produces greater initial $H^{+}$ efflux with more efficient use of ATP, but cytosolic pH recovery is reduced at steady-state. Furthermore, although changes in $H^{+}$/ATP ratio can influence steady-state pH recovery, this ratio is a static quantity that is fixed for a particular species.^64^^,^^65^^,^^66^ Therefore, the governing dynamic quantity for the F-ATPase response under acid-stress is Δψ, since the membrane depolarization (shift from large Δψ to small Δψ) enables cytosolic pH recovery.

### pH gating in KcsA imparts pH sensing and control to F-ATPase

We modeled F-ATPase as the sole driver of cytosolic pH recovery under acid-stress, and demonstrated that this recovery is governed by shifts in the electrical potential Δψ (Figure 1). We then modeled how shifts in Δψ can occur through other ion transporters, and explore the additional properties that these shifts impart to the cytosolic pH response. Notably, our prior single-transporter model did not account for changes in Δψ because of ion flux, including the $H^{+}$ efflux through F-ATPase. Without counter-ion transport, $H^{+}$ efflux through F-ATPase is Δψ-limited and cannot generate a large ΔpH. For many bacteria, this Δψ-limitation is overcome by $K^{+}$ influx, which depolarizes the cell membrane, thereby enabling the conversion of Δψ to ΔpH.^17^^,^^40^^,^^44^ However, this $K^{+}$ influx must be controlled to prevent complete depolarization, depletion of ATP, and over-alkalinization of the cytosol.^45^^,^^67^ Control of $K^{+}$ channels can take the form of feedback regulation by ATP and ADP.^68^ However, we hypothesize that this control can also be achieved by interactions between the ΔpH, $\Delta \left[K^{+}\right]$, and Δψ gradients. To investigate how this control can be effected, we extended our single-transporter model to include $K^{+}$ currents through KcsA. To isolate gradient interactions, we excluded consideration of $K^{+}$ movement through high-affinity $K^{+}$ transport systems such as Kdp; this exclusion is justified in environments where potassium is sufficient, as Kdp is expressed only at very low extracellular $\left[K^{+}\right]\text{.}$^69^

**Figure 2:**
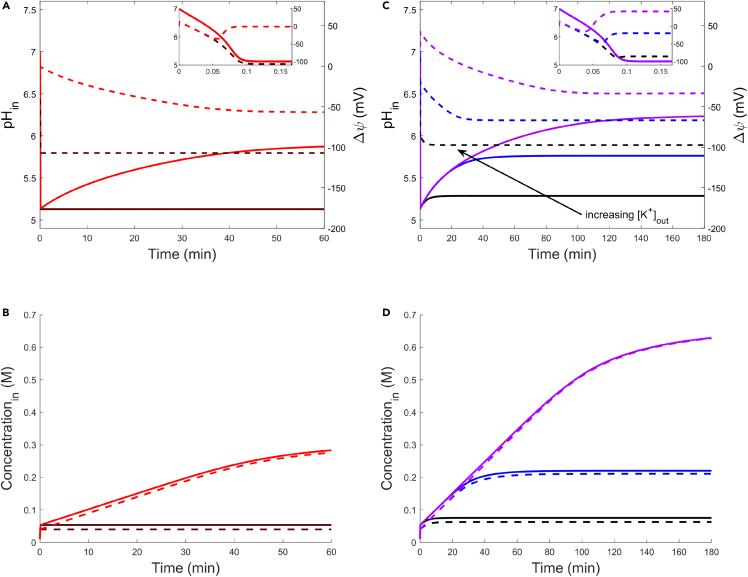
Cytosolic pH and membrane potential ( Δψ) driven by F-ATPase and KcsA activities under lactic acid shock (A) Dynamics of cytosolic pH (solid lines) and Δψ (dashed lines) with KcsA activity (red) or without KcsA activity (brown). Inset shows dynamics for the first 10 s. Initial cytosolic pH is 7.0, initial Δψ is 0 mV, and acid-stress is 50 mM lactate at an external pH of 5. (B) Internal lactate (solid lines) and $K^{+}$ (dashed lines) accumulation corresponding to (A). Initial internal lactate and $K^{+}$ concentrations are 10 mM and 40 mM, respectively. (C) Dynamics of cytosolic pH (solid lines) and Δψ (dashed lines) at varied extracellular $\left[K^{+}\right]$. Extracellular $\left[K^{+}\right]$ is set to 2 mM (black), 20 mM (blue), or 200 mM (purple). Inset shows dynamics for the first 10 s. Initial cytosolic pH is 7.0, initial Δψ is 0 mV, and external acid-stress is 50 mM lactate at an extracellular pH of 5. (D) Internal lactate (solid lines) and $K^{+}$ (dashed lines) accumulation corresponding to (C). Initial internal lactate and $K^{+}$ concentrations are 10 mM and 40 mM, respectively.

KcsA kinetics were calculated using a published kinetic expression,^12^^,^^70^^,^^71^ modified here to include pH gating.^48^ With this expression, we modeled the two-transporter response, where the acid stress was the same as in the prior single-transporter model (Figure 2). In Figure 2A, we model the cytosolic pH (solid lines) and Δψ (dashed lines) responses to acid stress, where KcsA is not expressed (brown) or KcsA is expressed (red). As with the single transporter model, the cytosolic pH sharply decreased to near 5 because of the influx of lactic acid. As F-ATPase pumped $H^{+}$ from the cytosol, the membrane polarized; in the case where KcsA was not expressed, Δψ decreased to near −100mV and prevented further $H^{+}$ efflux. In the case where KcsA was expressed, it activated at low cytosolic pH, enabling the influx of $K^{+}$. The cationic influx caused a positive shift in Δψ (Figure 2A, inset), reversing the polarization caused by F-ATPase. At a more positive Δψ, $H^{+}$ efflux by F-ATPase resumed, and the cytosolic pH partially recovered. The response characteristics and steady-state values for Δψ and cytosolic pH were largely insensitive to initial intracellular $K^{+}$ concentration (see Figure S3A), though experimental studies of similar transport interactions are typically conducted with $K^{+}$-depleted cells.^13^ Although steady-state required an hour or longer to reach, it is notable that the Δψ-mediated response initiates within seconds of the acid stress event. The fast dynamics of this response are because of the cell membrane’s low capacitance; as a result, small ionic currents can polarize or depolarize the cell membrane, and the membrane potential is almost completely insensitive to its initial value (see Figure S3B). The behavior of Δψ and cytosolic pH are supported by the concentrations of intracellular lactate and $K^{+}$, shown in Figure 2B. The lactate concentration matched the expected partition given the ΔpH, and the increase in $K^{+}$ concentration tracked closely with lactate when KcsA was expressed. At steady-state, the total PMF (sum of Δψ and ΔpH) was constant with or without the expression of KcsA, though KcsA expression was required to transduce Δψ to ΔpH.

Experimental measurements have demonstrated that the interchange of Δψ and ΔpH requires extracellular potassium and occurs in a concentration-dependent manner.^44^^,^^72^ To investigate the dynamics of this behavior, we repeated the simulation where KcsA was expressed, but we varied external $\left[K^{+}\right]$ (Figures 2C and 2D). Holding other conditions the same as previously, we set ${\left[K^{+}\right]}_{\text{out}}$ to 2 mM (black), 20 mM (blue), or 200 mM (purple). We chose the lower bound for this concentration range (2 mM) such that we could exclude the consideration of additional, high-affinity $K^{+}$-uptake systems.^69^ In Figure 2C, the cytosolic pH sharply decreased because of lactic acid influx, accompanied by a simultaneous decrease in Δψ. As the cytosol was acidified, $K^{+}$ influx through KcsA induced depolarization of the membrane (Figure 2C, inset), which maintained the cytosolic pH response through F-ATPase. As Δψ reached a steady state, F-ATPase approached its thermodynamic limit, and the cytosolic pH recovery stalled for all $K_{\text{out}}^{+}$ concentrations. Both the magnitude of the Δψ depolarization response and the steady-state depolarization of the membrane increased with ${\left[K^{+}\right]}_{\text{out}}$. With a more depolarized membrane, F-ATPase generated a larger ΔpH; this is confirmed by the partition of lactate within the cytosol, which increases with ${\left[K^{+}\right]}_{\text{out}}$ (1D). These results demonstrate that extracellular $K^{+}$ facilitates the recovery of cytosolic pH under acid stress, and they agree with known gradient interactions in acid-stressed bacteria: namely, that a large Δψ prevents the generation of a ΔpH,^73^ that $K^{+}$-induced depolarization enables the formation of a ΔpH,^13^^,^^41^ and that the distribution of PMF components favors ΔpH as ${\left[K^{+}\right]}_{\text{out}}$ increases.^44^

Membrane depolarization relaxed the PMF limitation on the F-ATPase and enabled pH recovery, such that ΔpH generation required KcsA expression and increased with ${\left[K^{+}\right]}_{\text{out}}$. When coupled, F-ATPase and KcsA generated two ionic gradients; F-ATPase generated a ΔpH and a Δψ, whereas KcsA transduced Δψ to a $\Delta \left[K^{+}\right]$. KcsA mediated control of F-ATPase through Δψ, because of the thermodynamic limitations imposed by the PMF. Conversely, F-ATPase controlled KcsA through the cytosolic pH, because of the pH-gated filter of KcsA (see Figure S3C for the Δψ and cytosolic pH response where KcsA gating is removed). As such, these results demonstrate pH sensing by a two-way control between F-ATPase and KcsA.

### Potassium uptake maintains PMF during fermentation

With the single-transporter and two-transporter models, we demonstrated how the cytosolic pH response is mediated by Δψ and how the pH-gating characteristics of KcsA impart cytosolic pH control to F-ATPase. The model allows tuning of transporter expression levels, thermodynamic/gating characteristics of transporters, and extracellular stress conditions; consequently, our results generalize across systems where the same electrochemical gradients predominate and where these transporters are conserved as the means of gradient transduction. To demonstrate a specific case application of this model, we simulated the generation of electrochemical gradients and transduction of energy during homolactic fermentation. In tandem with our two-protein stress response model, we simulated biomass, ATP, and lactic acid formation from glucose in phosphate-buffered medium containing ammonium and amino acids. To calculate growth and lactic acid production rates, we modified Monod-based expressions to include inhibition at low ${\text{pH}}_{\text{in}}$, such that biomass formation ceases at a ${\text{pH}}_{\text{in}}$ of 6 and metabolism ceases at a ${\text{pH}}_{\text{in}}$ of 5. These values were chosen according to evidence that biomass formation ceases at a higher pH than metabolism,^19^ and that lactic acid bacteria can maintain activity near an internal pH of 5.^74^^,^^75^ In addition, we calculated the total (per cell) energy stored as ATP, $\Delta \left[K^{+}\right]$, ΔpH, and Δψ. From literature measurements and our prior models, we expected that $K^{+}$ influx would facilitate the generation of a ΔpH, and maintain the PMF at a near-constant value during fermentation.^13^^,^^42^^,^^76^ We also expected that the Δψ and ΔpH would contribute a small fraction of cellular energy, because of the low electrical capacitance of the cell and the low cytosolic concentrations of $H^{+}$.

**Figure 3:**
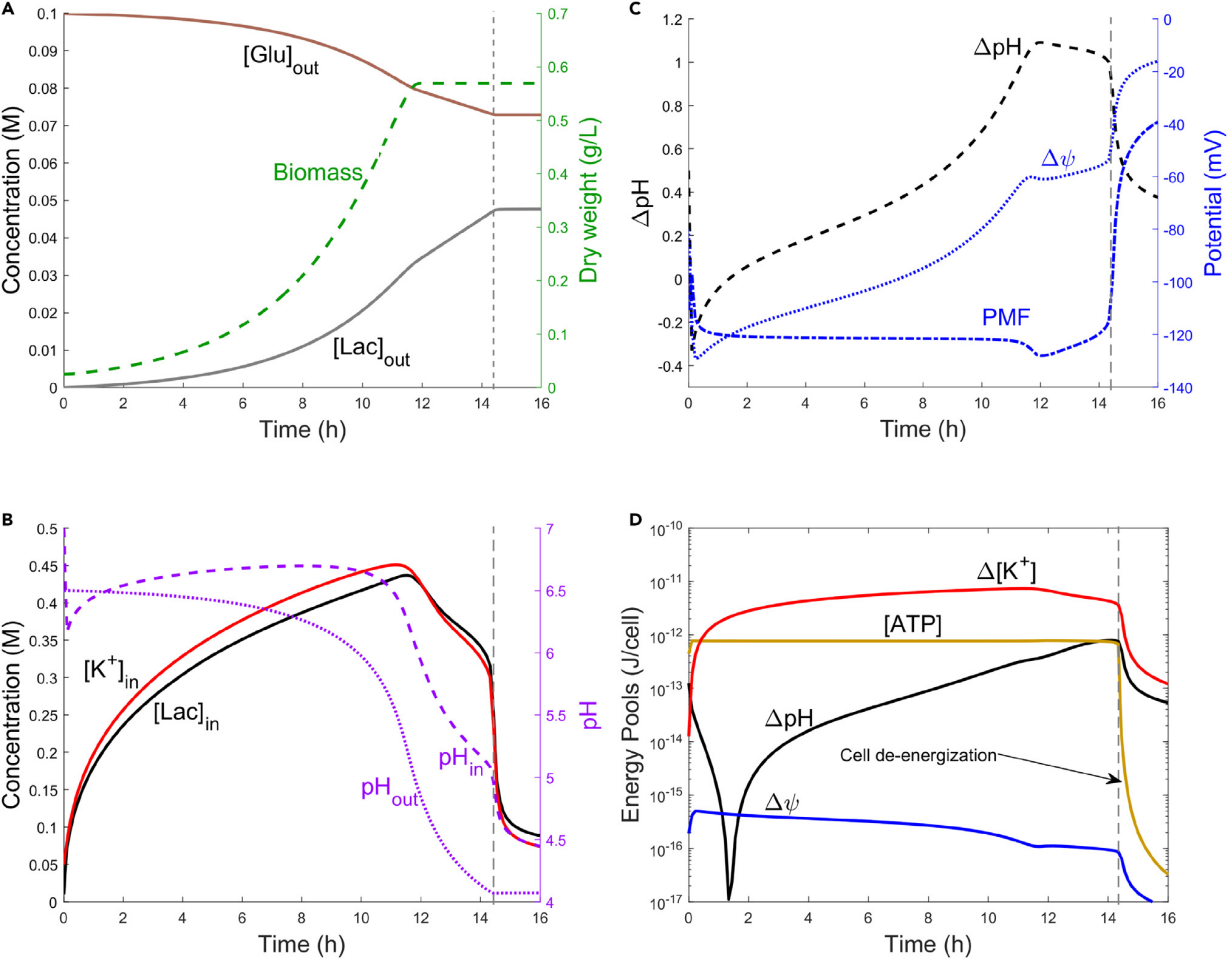
Biomass, internal/external solute concentrations, internal/external pH, potential gradients, and cellular energy pools during lactic acid fermentation (A) Extracellular glucose (brown, solid), extracellular lactate (gray, solid), and dry biomass (green, dashed) concentrations during fermentation. (B) Left axis: Intracellular lactate (black, solid) and $K^{+}$ (red, solid) concentrations. Right axis: intracellular pH (dashed) and extracellular pH (dotted). (C) Energetic gradients during fermentation. Left axis: ΔpH. Right axis: Δψ and total PMF. (D) Cellular energy pools during fermentation, in terms of total energy per cell. For the $\Delta \left[K^{+}\right]$, ΔpH, and Δψ energy pools, energy is calculated as the sum of available energy from fully depleting the gradient ($\Delta \left[K^{+}\right]$ and ΔpH) or depolarizing the membrane (Δψ). For the ATP pool, energy is calculated as the sum of available energy from total hydrolysis of ATP to ADP and Pi.

Simulation results are presented in Figure 3. During fermentation, glucose was consumed to generate biomass or to generate ATP and internal lactic acid (Figure 3A), which resulted in acidification of the cytosol (Figure 3B). Initially, $H^{+}$ efflux through F-ATPase generated a large and negative Δψ, which arrested further efflux (Figure 3C). As the cytosolic pH decreased, the pH-gated KcsA channel opened, causing an influx of $K^{+}$ (Figure 3B) and a corresponding positive shift in Δψ. At a more positive Δψ, F-ATPase generated a larger ΔpH, maintaining the cytosolic pH as the extracellular medium was acidified. Even though the Δψ and ΔpH components interchange, the total PMF remained constant near 120 mV over the course of the fermentation (about 14 h, Figure 3C). As a result of the combined activities of F-ATPase and KcsA, the cytosolic pH was maintained at near neutral values for most of the fermentation.

As the ΔpH increased above 1.0 (about 12 h), the combined activities of F-ATPase and KcsA could no longer maintain the cytosolic pH at optimal values. The potential required to influx $K^{+}$ against the concentration gradient approached the value of Δψ, such that no further $K^{+}$ influx occurred and Δψ stabilized. Consequently, $H^{+}$ efflux through F-ATPase was slowed and eventually arrested by PMF limitations, because of simultaneous contributions of Δψ and ΔpH. As the internal pH decreased to 6.0, biomass formation ceased. Metabolism continued to consume glucose and generate ATP and lactate until the internal pH reached 5.0, at which point ATP production ceased, ΔpH collapsed, and the membrane was depolarized. The cessation of metabolism and collapse of energetic gradients, or “cell de-energization” (Figure 3), is the direct result of the form of the Monod-based expression for metabolism, where the glycolysis rate approaches zero as the internal pH approaches 5 (see STAR Methods).

During lactic acid fermentation, the cell continuously exchanges between the energetic “pools” of ATP, ΔpH, Δψ, and $\Delta \left[K^{+}\right]$.^76^ These pools represent the total amount of energy available to the cell through ATP hydrolysis or through dissipation of an electrical or ionic gradient. To understand the sizes and dynamics of these different energy pools, we calculate the total and specific energies stored as ATP, ΔpH, Δψ, and $\Delta \left[K^{+}\right]$ gradients. Total energy pools, on a per-cell basis, are presented in Figure 3D, whereas specific energies are shown in Figure S4. The specific energy of each gradient, defined as the free energy per mole of ions transported, was about 10–20% of the energy of ATP hydrolysis. In comparing total energy capacity (pools), the energy stored in Δψ was more than 1000-fold lower than the ATP pool, as expected. However, the ionic gradients held a substantial energy capacity: the $\Delta \left[K^{+}\right]$ pool held 10-fold greater energy than the ATP pool, and the ΔpH pool held as much energy as the ATP pool near the end of the fermentation. Although the capacity of the $\Delta \left[K^{+}\right]$ pool can be attributed to the large internal concentration of $K^{+}$, the capacity of the ΔpH pool was surprising because of the small number of free $H^{+}$ per cell.^1^ These results demonstrate that maintenance of homeostasis during lactic acid fermentation requires dynamic shifts in the Δψ, ΔpH, and $\Delta \left[K^{+}\right]$ gradients, and that these gradients can hold a total energy comparable to, or greater than, the energy contained as intracellular ATP.

## Discussion

Computational models of bacteria have grown in sophistication and complexity, fueled by vast troves of available genomic, transcriptomic, and metabolomic data.^77^^,^^78^^,^^79^ Yet, relatively few models have focused on the role of electrical and ionic gradients, even though these gradients constitute a substantial fraction of the cellular energy pool,^7^^,^^30^ and drive transport-based uptake and stress response.^11^^,^^44^^,^^80^ This is partially because of the challenges of gradient measurement, and partially because of assumptions that these gradients are mere “homeostatic contributors.”^1^ With the advent of improved gradient indicators and imaging techniques,^14^^,^^81^ studies have since highlighted the dynamic and keystone role of electrochemical gradients in environmental sensing,^2^^,^^82^ cell-cell communication,^22^^,^^39^ metabolism,^43^ and sporulation.^83^ Outside of such well-studied systems, however, much remains unclear concerning the interplay between transporters, electrochemical gradients, and bacterial behavior.^1^ Due to the complex and rapid interactions of gradients and transporters, experiment alone cannot bridge this gap.^14^ Electrochemical gradient modeling provides a general framework to understand these interactions and their underlying mechanisms, complementing the existing suite of bacterial models. Here, we demonstrated that a simple gradient model yields insights into the energetic limitations of transport, elucidates mechanisms for bacterial survival under stress, and predicts bacterial behavior across different environments.

In this model, we quantified the generation, maintenance, and interactions of electrical, proton, and potassium potential gradients under lactic acid-stress and lactic acid fermentation. Using kinetic models of KcsA and F-ATPase, we demonstrated that electrogenic $K^{+}$ transport imparts pH-sensing to the acid-stress response. Under acid-stress, $K^{+}$ transport is necessary for $H^{+}$ efflux^40^ and pH homeostasis.^11^^,^^44^^,^^79^^,^^84^ This has long-been understood to be because of membrane polarization (a large and negative Δψ), as PMF limitations on F-ATPase prevent the simultaneous maintenance of large Δψ and ΔpH.^4^^,^^42^ Addition of extracellular $K^{+}$ to potassium-starved cells depolarizes the cell membrane by dissipating Δψ, enabling generation of a ΔpH.^17^^,^^40^^,^^44^ This agrees with our two-protein model, where $K^{+}$ influx through KcsA depolarizes the cell membrane, enabling an increase in cytosolic pH because of F-ATPase activity. Membrane depolarization can also be accomplished by addition of a $K^{+}$ ionophore; at low external pH, this causes the PMF components to interchange in a similar manner.^40^ At neutral pH, however, $K^{+}$ ionophores cause over-alkalinization of the cytosol, which does not occur with channel-mediated $K^{+}$ transport.^45^ Our model indicates that this discrepancy is caused by pH-gating in $K^{+}$ transport. At high cytosolic pH, the $K^{+}$ channel KcsA exists in a closed state, which blocks $K^{+}$ influx even for large Δψ.^48^^,^^85^ As relatively few ions are required to polarize the cell membrane, F-ATPase generates a PMF with Δψ as the major component.^40^ As the cytosolic pH decreases, KcsA shifts to the open state,^85^ permitting the influx of $K^{+}$ and subsequent depolarization of the membrane. By this mechanism, the pH-gating of KcsA controls $H^{+}$ efflux through F-ATPase, and therefore functions as a cytosolic pH sensor for the acid-stress response. This sensing occurs without direct interaction between F-ATPase and KcsA, and without indirect metabolite-based gating, as has been found in other systems such as TrkA/TrkH.^68^ Both metabolite-based and charge transport-based emergent interactions are known to occur at the cell-cell level; such interactions drive much of the robustness and metabolic flexibility found within mixed microbial communities.^86^^,^^87^^,^^88^^,^^89^ Notably, however, gradient-mediated pH-sensing occurs within a single cell, arising from emergent interactions because of a shared gradient (Δψ) between different transporters.

Key to this pH-sensing is the limitation of F-ATPase activity by Δψ. $H^{+}$ efflux through F-ATPase is electrogenic, and therefore controlled by Δψ.^40^^,^^59^ Consistent with this notion, we found that Δψ limits steady-state pH recovery under acid stress, and that over-expression of F-ATPase cannot overcome this limitation. However, F-ATPase expression does increase under acid stress,^56^^,^^80^^,^^90^ though this increase is part of a multifaceted response that includes membrane depolarization.^8^^,^^19^^,^^91^ With a polarized membrane (large Δψ), F-ATPase is thermodynamically limited, and the kinetic differences between F-ATPase expression levels become imperceptible. With a depolarized membrane (small Δψ), we found that increased F-ATPase expression caused a transient increase in cytosolic pH, which may benefit cells by limiting acid stress-induced damage. Therefore, the limitation imposed by F-ATPase expression level is purely kinetic, as the equilibrium pH is not a function of expression level. Our findings give context to a recent review,^52^ which notes that increased F-ATPase expression maintains ${\text{pH}}_{\text{in}}$ in acid-stressed bacteria. To maintain $H^{+}$ efflux, increased F-ATPase expression must be accompanied by a depolarization response to avoid PMF-limitation. Notably, PMF-limitation of F-ATPase is multiplicative in effect with the $H^{+}$/ATP ratio, which is determined by the number of c-subunits in the $F_{0}$ domain.^32^^,^^59^^,^^66^ Although the c-subunit number is fixed for a given species, it varies greatly between organisms, and promotes survival specific to an extracellular environment.^61^ The $H^{+}$/ATP ratio determines the work required for $H^{+}$ efflux, which equals the free energy of ATP hydrolysis at equilibrium (no net transport). As the ratio increases, there is an increase in the efficiency: that is, the number of $H^{+}$ ions removed per molecule of ATP. However, there is a corresponding decrease in the power, or the maximal PMF at which $H^{+}$ can be removed. We found that this efficiency/power tradeoff favors a high $H^{+}$/ATP ratio at low Δψ. However, acidic environments do not exclude the existence of high $H^{+}$/ATP ratios, which are found in some acidophilic bacteria.^63^ Rather, a high $H^{+}$/ATP ratio provides more efficient use of ATP, at the cost of requiring a small or positive Δψ, or tolerance of a more acidic cytosol. The bacterial acid-stress and acid-shock responses can potentially be improved by manipulating the $H^{+}$/ATP ratio, such as by engineering chimeric ATPases with variable c-subunit stoichiometries.^62^ By quantifying the tradeoffs between cytosolic pH maintenance, Δψ maintenance, and consumption of ATP, this model predicts the conditions where such engineering strategies are likely to succeed.

As previously discussed, $H^{+}$ efflux is controlled by the interactions of the ΔpH, $\Delta [K^{+}]$, and Δψ gradients. However, these gradients are not static quantities; rather, they function as energy pools that vary with cellular metabolism and the extracellular environment.^1^^,^^7^ Accordingly, we modeled these gradients in concert with acid-fermentation metabolism and calculated the size of each energy pool. We found that the Δψ and ΔpH gradients interchanged over the course of the fermentation, maintaining a near-constant PMF. These results show good agreement with literature data over the same external pH range,^76^ as does our estimate for ΔpH under acid stress.^75^^,^^92^^,^^93^ However, the similar energy content of the ΔpH and ATP pools was unexpected, given that there are few free protons per bacterium.^1^ This result is explained by the large buffering capacity of the cytosol, where phosphate and lactate quickly equilibrate with free $H^{+}$. Owing to the high buffer concentrations and fast kinetics of $H^{+}$-transfer reactions, stochastic phenomena would play a smaller role than expected in $H^{+}$ efflux.^94^ In addition, we found that the Δψ pool contains far less energy than available from ATP hydrolysis or ionic gradient dissipation; in contrast, the specific energy of Δψ (or energy per monovalent ion transported) is more than 20% of the specific energy of ATP hydrolysis^95^ (Figure S4). Together, these findings demonstrate the energetic advantages of Δψ-mediated stress response. Owing to its small size, the Δψ pool exhibits rapid turnover and high sensitivity to changes in ion flow.^16^ However, since transport favorability depends on specific energy, Δψ controls transport rates while undergoing rapid changes at little energetic cost to the cell. Consequently, Δψ offers a sensitive and efficient means of sensing changes to the extracellular environment and maintaining homeostasis.

The model presented here elucidates how electrochemical gradients can control the acid-stress response and how Δψ can function as a highly sensitive and dynamic pH sensor and regulator. By capturing and predicting the dynamics and interactions of electrochemical gradients, this model bridges the experimental gap caused by the large number of potential interactions and by measurement techniques that fail to capture rapid dynamics.^14^ The identified gradient interactions offer targets for future experimental studies, supported by the development of specialized measurement methods with improved dynamics.^96^^,^^97^ By including additional transport systems and ionic gradients, this model can be adapted to other organisms and extracellular environments. This model offers insights on the control of electrochemical gradient interactions and their influence on bacterial behavior, which will inform strategies for organism engineering and growth optimization.

### Limitations of the study

This model captures aspects of transport-gradient-behavior interactions in acid-stressed lactic acid bacteria, but extending these results to other extracellular conditions and organisms would require further theoretical development and experimental validation. For example, modeling bacterial behavior in a potassium-deficient medium would necessitate the inclusion of high affinity transporters, such as the potassium pump Kdp. Kdp can generate a 50,000-fold $\left[K^{+}\right]$ gradient^98^ through the combined driving force of Δψ and ATP hydrolysis, corresponding to an outward potassium ion-motive force (IMF) of greater than 250 mV. But, Kdp is repressed at extracellular potassium concentrations above 2 mM,^69^ such that its effect on potassium IMF and ATP consumption can be excluded in this model. Lactic acid bacteria express other high-affinity $K^{+}$-transport systems such as KupA/KupB, as well as constitutively expressed transporters such as Trk/Ktr, which predominate at near-neutral pH.^72^ However, both KupA/KupB and Trk/Ktr are inhibited by cyclic-di-AMP,^99^^,^^100^ which is expected to increase under acid stress.^101^ Inclusion of these $K^{+}$ transport systems would be necessary to extend the model to potassium-deficient or higher-pH environments, or to organisms with reduced or non-existent cyclic-di-AMP.^102^^,^^103^ Similarly, extending this model to respiring bacteria would require inclusion of the electron-transport chain and its effect on Δψ. Additional challenges are posed by other acidophiles, such as archaea, which possess $K^{+}$ uptake systems that are poorly characterized.^104^ In this case, both a theoretical model of the transporter and experimental validation of the transport rate expression are required for the model to be valid. Experimental validation is also particularly important for conditions where other ionic gradients predominate (e.g. saline conditions), as models for both additional transporters and additional ionic gradients ($\Delta \left[{\text{Na}}^{+}\right]$) would need to be developed. Lastly, multi-component model extensions are required for eukaryotes such as yeast, which encounter acid-stress in many renewable and industrial bioprocesses.^105^^,^^106^^,^^107^^,^^108^^,^^109^ In this case, multiple cellular components (e.g., vacuole and plasma membrane) must be modeled in tandem, because of the combined effect of vacuolar and plasma membrane ATPases in maintaining cytosolic pH under acid-stress.^109^^,^^110^ For the wide range of extracellular conditions and cellular physiologies, the increasing availability of kinetic and expression data for transport systems will aid in extending the theoretical framework developed herein.^11^^,^^30^^,^^78^

## STAR★Methods

### Key resources table

**Table:** 

REAGENT or RESOURCE	SOURCE	IDENTIFIER
**Software and algorithms**		

Source code	This study	Data S1.zip
Source code for parent model	Liao et al.^35^	https://doi.org/10.1073/pnas.1423143112
pHtools source code	Dougherty et al.^111^	Data S1.zip
Matlab R2018a	MathWorks	https://www.mathworks.com/products/matlab.html

### Resource availability

#### Lead contact

Further information and requests for resources should be directed to and will be fulfilled by the lead contact, Marcus Benyamin (marcus.s.benyamin2.civ@army.mil).

#### Materials availability

This study did not generate new unique reagents.

### Method details

We model the dynamics of Δ pH, Δψ, and $\Delta \left[K^{+}\right]$ during lactic acid stress, and then extend this model to include biomass and lactic acid production during fermentation. To do so, we first review the calculation of Δψ and lactic acid influx rates, and derive rate equations for the transport proteins F-ATPase and KcsA. Using these rate equations, we build a system of ordinary differential equations (ODEs) to predict intracellular/extracellular solute concentrations and growth rate. We then solve these ODEs using the MATLAB solver ode15s, adapting the code framework and pHtools solver from Liao et al.^35^ and Dougherty et al.,^111^ respectively. Model parameters are given in Table S1.

#### Membrane potential

Changes in Δψ occur by any net movement of charge across the cell membrane; proteins that facilitate such charge transport (such as ion transporters) are considered electrogenic. We can express Δψ as a function of ions accumulated across the cell membrane,^16^ as given by Equation 1. For *n* different ionic solutes, *Z* is the ionic charge (Coulombs per mol), *m* is the amount of ions (mol) accumulated in transport across the cell membrane, and *C* is the cell membrane capacitance (Farads).

(Equation 1) $$\Delta \psi =\frac{1}{C}\sum_{i=1}^{n}Z_{i}m_{i}$$

In electrogenic transport, ions remain close to the cell membrane or exchange with ions in the bulk solution.^16^ As a result, *m* in Equation 1 corresponds to transport-accumulated ions, rather than the bulk ionic concentration. To relate Δψ to the rate of ionic transport, we take the derivative of 1, and find, for a constant cell membrane capacitance:

(Equation 2) $$\frac{d\Delta \psi }{dt}=\frac{1}{C}\sum_{i=1}^{n}Z_{i}\frac{dm_{i}}{dt}$$

Equation 2 predicts changes in Δψ by the sum of all ionic transport processes. In this model, the specific processes considered are $H^{+}$ transport through F-ATPase and $K^{+}$ transport through KcsA.

#### Lactic acid diffusion

Under lactic acid-stress, extracellular lactate is present at an acidic external pH. Following Liao et al.,^35^ we assume that only undissociated lactic acid permeates the cell membrane. Accordingly, undissociated acid concentrations for external and internal lactate are calculated using Equations 3 and 4, respectively.

(Equation 3) $${\left[HLac\right]}_{out}=\frac{{\left[Lac_{total}\right]}_{out}}{1+10^{pH_{out}-pKa_{Lac}}}$$

(Equation 4) $${\left[HLac\right]}_{in}=\frac{{\left[Lac_{total}\right]}_{in}}{1+10^{pH_{in}-pKa_{Lac}}}$$

The rate of lactic acid influx is directly proportional to the concentration difference and the permeability constant P ($s^{-1}$), as given in Equation 5.

(Equation 5) $$r_{influx}=P\left({\left[HLac\right]}_{out}-{\left[HLac\right]}_{in}\right)$$

For both the cytosol and extracellular medium, phosphate acts as the buffering agent, and acidification is caused by the presence or production of lactic acid. The buffering capacity of other medium constituents such as amino acids are expected to be small^112^ and are therefore omitted from pH calculations.

#### $F_{1}F_{0}$-ATPase

$F_{1}F_{0}$-ATPase (F-ATPase) is a membrane-bound enzyme complex that couples the efflux of protons across the cell membrane to the hydrolysis of ATP. The complex consists of the catalytic $F_{1}$ domain and the membrane-integral rotary $F_{0}$ domain, which rotates in distinct 120 ${}^{\circ }$ steps each catalytic cycle.^58^ $H^{+}$ efflux generates a PMF composed of Δ pH and Δψ; consequently, we expect F-ATPase kinetics to exhibit interdependence with both substrate/product concentrations (ATP, ADP, and Pi) and driving forces (PMF). Here, we describe the stoichiometry and driving forces for F-ATPase, and derive a rate equation for F-ATPase kinetics using the equilibrium approximation.^113^ We first develop a simplified reaction schematic from the reported mechanism, identifying fast steps and a rate limiting (slow) step. We then identify electrogenic steps, where ions move through the transmembrane potential, and incorporate PMF-dependence into those steps. Finally, we determine equilibrium K-values for fast steps from published free-energy profiles, and check the resulting rate expression with published kinetic data. This derivation should generalize to building rate expressions for other electrogenic ATPase pumps.

The stoichiometry of $H^{+}$ transport by F-ATPase is given in Equation 6, where n is the $H^{+}$/ATP ratio:

(Equation 6) $$nH_{in}^{+}+ATP↔nH_{out}^{+}+ADP+Pi$$

Based on the published F-ATPase mechanism,^114^ we list simplified reaction steps (S1)-(S5) for the $F_{1}$ catalytic trimer complex. Each step involves one of three processes: binding/unbinding (steps (S1) and (S4), hydrolysis reaction (step (S3), and conformational change (steps (S2) and (S5). Our notation omits binding/unbinding to different catalytic subunits; rather, we denote the $F_{1}$ complex to exist in either the E1 or E2 conformation. The shift from E1 to E2 occurs by an 80 ${}^{\circ }$ rotation, and the return to E1 occurs upon a further 40 ${}^{\circ }$ rotation. As it rotates, the complex is bound to ATP, ADP, and Pi as denoted in the subscript. In accordance with published kinetic studies, Pi dissociation at step (S4) is assumed to be the rate-determining step (r.d.s.).^59^^,^^115^ At step (S5), the $F_{1}$ subunit returns to the E1 state after a total of 120 ${}^{\circ }$ of rotation, requiring two additional catalytic cycles to complete a full rotation. Since $H^{+}$ transport is associated with conformational changes that occure in steps (S2) and (S5), we assume that these steps are electrogenic.^59^

$$E1+ATP\overset{K_{1}}{⇌}E1_{ATP}+ADP\;\left(S1\right)$$

$$E1_{ATP}\overset{K_{2}}{⇌}E2_{ATP}\;\left(S2\right)$$

$$E2_{ATP}\overset{K_{3}}{⇌}E2_{ADP\cdot Pi}\;\left(S3\right)$$

$$E2_{ADP\cdot Pi}\underset{k_{b}}{\overset{k_{f}}{⇌}}E2_{ADP}+Pi\left(r.d.s\text{.}\right)\;\left(S4\right)$$

$$E2_{ADP}\overset{K_{5}}{⇌}E1\;\left(S5\right)$$

Under the equilibrium approximation, we assume steps S1, S2, S3, and S5 to be fast and at equilibrium. For each fast step, an equilibrium K-value relates the ratio of reactant and product concentrations, as given in Equations 7, 8, 9, and 11. For rate-determining step, the rate is expressed in terms of reaction intermediates, and is shown in Equation 10. In (Equation 7), (Equation 8), (Equation 9), (Equation 10), (Equation 11), solute concentration terms (bracketed) represent unbound ATP, ADP, and Pi, while enzyme concentration terms (unbracketed) represent the fraction of total enzyme that exists in each state.

(Equation 7) $$K_{1}=\frac{E1_{ATP}\cdot \left[ADP\right]}{E1\cdot \left[ATP\right]}$$

(Equation 8) $$K_{2}=\frac{E2_{ATP}}{E1_{ATP}}$$

(Equation 9) $$K_{3}=\frac{E2_{ADP\cdot Pi}}{E2_{ATP}}$$

(Equation 10) $$r_{ATPase}=k_{f}\cdot E2_{ADP\cdot Pi}-k_{b}\cdot E2_{ADP}\cdot \left[Pi\right]$$

(Equation 11) $$K_{5}=\frac{E1}{E2_{ADP}}$$

Since enzyme concentrations in (Equation 7), (Equation 8), (Equation 9), (Equation 10), (Equation 11) are fractions of the total, we assume the total enzyme concentration is unity and write Equation 12.

(Equation 12) $$1=E1+E1_{ATP}+E2_{ATP}+E2_{ADP\cdot Pi}+E2_{ADP}$$

We then choose E1 as the ”base state” for the enzyme and build an expression that includes only equilibrium K-values and solute concentrations, which yields Equation 13.

(Equation 13) $$E1=\frac{1}{1+\frac{E1_{ATP}}{E1}+\frac{E2_{ATP}}{E1}+\frac{E2_{ADP\cdot Pi}}{E1}+\frac{E2_{ADP}}{E1}}$$

Using (Equation 7), (Equation 8), (Equation 9), (Equation 10), (Equation 11), (Equation 12), (Equation 13), we relate the ratio of each enzyme state to the base state E1, deriving (Equation 14), (Equation 15), (Equation 16), (Equation 17).

(Equation 14) $$\frac{E1_{ATP}}{E1}=\frac{K_{1}\cdot \left[ATP\right]}{\left[ADP\right]}$$

(Equation 15) $$\frac{E2_{ATP}}{E1}=\frac{K_{2}K_{1}\cdot \left[ATP\right]}{\left[ADP\right]}$$

(Equation 16) $$\frac{E2_{ADP\cdot Pi}}{E1}=\frac{K_{3}K_{2}K_{1}\cdot \left[ATP\right]}{\left[ADP\right]}$$

(Equation 17) $$\frac{E2_{ADP}}{E1}=\frac{1}{K_{5}}$$

Combining (Equation 13), (Equation 14), (Equation 15), (Equation 16), (Equation 17), we express the fraction of enzyme in the E1 state in terms of only solute concentrations and equilibrium constants, yielding (18).

(Equation 18) $$E1=\frac{1}{1+\frac{K_{1}\cdot \left[ATP\right]}{\left[ADP\right]}+\frac{K_{2}K_{1}\cdot \left[ATP\right]}{\left[ADP\right]}+\frac{K_{3}K_{2}K_{1}\cdot \left[ATP\right]}{\left[ADP\right]}+\frac{1}{K_{5}}}$$

Based on our earlier rate Equation 10, the ratios (16) and (17), and our expression for E1 (18), we arrive at the overall kinetic expression for F-ATPase in Equation 19.

(Equation 19) $$r_{ATPase}=\frac{k_{f}\cdot \frac{K_{3}K_{2}K_{1}\cdot \left[ATP\right]}{\left[ADP\right]}-k_{b}\cdot \frac{\left[Pi\right]}{K_{5}}}{1+\frac{K_{1}\cdot \left[ATP\right]}{\left[ADP\right]}+\frac{K_{2}K_{1}\cdot \left[ATP\right]}{\left[ADP\right]}+\frac{K_{3}K_{2}K_{1}\cdot \left[ATP\right]}{\left[ADP\right]}+\frac{1}{K_{5}}}$$

With Equation 19, the reaction rate is expressed in terms of solute concentrations and equilibrium K-values. For the rate-determining step, the forward rate constant $k_{f}$ is averaged from literature values,^116^ while the backward rate constant is calculated to satisfy the equilibrium condition in (20), where the forward and backward rates must be equal:

(Equation 20) $$k_{f}\cdot K_{3}K_{2}K_{1}\cdot K_{eq}^{ATPsynthesis}=k_{b}\cdot \frac{1}{K_{5}}$$

For each fast step *i*, equilibrium K-values are calculated according to Equation 21. The standard free energy of each step ($\Delta G_{i}^{\circ }$) is taken from a published free energy diagram of the reaction.^117^

(Equation 21) $$K_{i}=e^{-\Delta G_{i}/RT}$$

From the rate expression (19), we incorporate the PMF into the rate expression by considering electrogenic steps (S2) and (S5). We do so by following the mathematical treatment presented in Gao et al.,^59^ and treat Δ pH and Δψ as components of an external load that is applied to reaction steps (S2) and (S5). The total work under an external load is given by Equations 22 and 23, where $\Delta \mu_{H^{+}}$ is the chemical potential of $H^{+}$. In (23), *F* is Faraday’s constant, *R* is the universal gas constant, and T is the absolute temperature (Kelvin).

(Equation 22) $$W_{total,load}=W_{rotation}+n\Delta \mu_{H^{+}}$$

(Equation 23) $$W_{total,load}=W_{rotation}+n\left(-F\Delta \psi +RTln\left(\frac{{\left[H^{+}\right]}_{out}}{{\left[H^{+}\right]}_{in}}\right)\right)$$

The external load of (23) is distributed between steps (S2) and (S5). We parameterize this distribution as *x*, where *x* is the fraction of the load assigned to (S2). Since the torque profile of F-ATPase is constant,^60^ we assign 2/3 of the load to the 80 ${}^{\circ }$ rotation of (S2) and the remaining 1/3 to the 40 ${}^{\circ }$ rotation of (S5). Since work is equivalent to $\Delta G_{i}^{\circ }$ for these steps, $K_{2}$ and $K_{5}$ are recalculated using (23) and (21):

(Equation 24) $$K_{2,load}=e^{-\frac{2}{3}\frac{W_{total,load}}{RT}}$$

(Equation 25) $$K_{5,load}=e^{-\frac{1}{3}\frac{W_{total,load}}{RT}}$$

The complete rate Equation 26, then, is:

(Equation 26) $$r_{ATPase}=\frac{k_{f}\cdot \frac{K_{3}K_{2,load}K_{1}\cdot \left[ATP\right]}{\left[ADP\right]}-k_{b}\cdot \frac{\left[Pi\right]}{K_{5,load}}}{1+\frac{K_{1}\cdot \left[ATP\right]}{\left[ADP\right]}+\frac{K_{2,load}K_{1}\cdot \left[ATP\right]}{\left[ADP\right]}+\frac{K_{3}K_{2,load}K_{1}\cdot \left[ATP\right]}{\left[ADP\right]}+\frac{1}{K_{5,load}}}$$

Rewritten for simplification, the rate equation becomes:

(Equation 27) $$r_{ATPase}=\frac{k_{f}K_{5,load}K_{3}K_{2,load}K_{1}\cdot \left[ATP\right]-k_{b}\cdot \left[Pi\right]\left[ADP\right]}{\left[ADP\right]\left(K_{5,load}+1\right)+\left[ATP\right]K_{1}K_{5,load}\left(1+K_{2,load}+K_{3}K_{2,load}\right)}$$

With Equation 27, we have developed a rate expression for F-ATPase that depends on substrate concentrations and PMF. The forward (ATP hydrolysis) reaction in expression (27) resembles Michaelis-Menten kinetics with respect to [ATP], and the backwards (ATP synthesis) reaction resembles Michaelis-Menten kinetics with respect to [ADP] and first-order kinetics with respect to [Pi]. While the rate decreases without bound as [Pi] increases, this is a consequence of assuming that phosphate dissociation is the rate-determining step. As we hold cytosolic phosphate concentration to be constant, the assumption is reasonable in this model.

We plot the ATP hydrolysis rate as a function of PMF in Figure S1 for different PMF values and ADP concentrations. At non-limiting ATP and ADP concentrations, the maximum F-ATPase hydrolysis/synthesis rates at high and low PMF show good agreement with other studies.^59^^,^^118^ Further, the reaction rate is zero (intersections with dashed lines) where the free energy of ATP hydrolysis is equal to the free energy of $H^{+}$ transport, so the rate equation is thermodynamically consistent.

#### KcsA

To model the behavior of $K^{+}$ transport, we build a rate equation for the potassium channel KcsA, which conducts $K^{+}$ according to Equation 28.

(Equation 28) $$K_{out}^{+}⇌K_{in}^{+}$$

As there is a net transport of charge in 28, $K^{+}$ transport will also affect Δψ in accordance with (2). KcsA transports $K^{+}$ both quickly ($10^{8}s^{-1}$) and selectively over other metal ions,^119^^,^^120^ and is driven entirely by the $K^{+}$ concentration gradient and Δψ. At equilibrium, the two driving forces must sum to zero, as given by Equation 29.

(Equation 29) $$-F\Delta \psi =RTln\left(\frac{{\left[K^{+}\right]}_{in,eq}}{{\left[K^{+}\right]}_{out,eq}}\right)$$

From (29), we can check the thermodynamic consistency of the KcsA rate expression. We begin with the association/dissociation (AD) model for KcsA kinetics,^12^^,^^70^^,^^71^ which yields the rate expression in Equation 30. Under the AD model, KcsA activity is a function of ${\left[K^{+}\right]}_{\text{in}}$, ${\left[K^{+}\right]}_{\text{out}}$, and Δψ, and exhibits Michaelis-Menten saturation for each. The overall rate of $K^{+}$ transport, then, is:

(Equation 30) $$r_{KcsA}=\frac{k_{d}\left({\left[K^{+}\right]}_{out}e^{-\alpha f\Delta \psi }-{\left[K^{+}\right]}_{in}e^{\left(1-\alpha \right)f\Delta \psi }\right)}{K_{d}\left(e^{-\alpha f\Delta \psi }+e^{\left(1-\alpha \right)f\Delta \psi }\right)+\left({\left[K^{+}\right]}_{in}+{\left[K^{+}\right]}_{out}\right)}$$

In (30), α is the charge transfer coefficient for $K^{+}$ transport and is assumed to be 0.5 for a symmetrical reaction. The parameter *f* is used to simplify the expression by combining Faraday’s constant F, the gas constant R, and the temperature T as given in (31):

(Equation 31) $$f=\frac{F}{RT}$$

The numerator of (30) is the Butler-Volmer equation for $K^{+}$ transport, so the rate expression satisfies (29) and is thermodynamically consistent. Equation 30 describes KcsA kinetics only at low cytoplasmic pH, where the channel exists in a conductive state. However, KcsA contains a pH-sensitive filter that closes at high internal pH, blocking $K^{+}$ transport.^48^^,^^49^ We incorporate pH-dependence of KcsA activity in the rate expression (32) by including the ${\text{pK}}_{a}$ of the KcsA pH filter. The ${\text{pK}}_{a}$ is 4.2 for the model protein, with a Hill coefficient of 2.^48^

(Equation 32) $$r_{KcsA,gated}=\frac{k_{d}\left({\left[K^{+}\right]}_{out}e^{-\alpha f\Delta \psi }-{\left[K^{+}\right]}_{in}e^{\left(1-\alpha \right)f\Delta \psi }\right)}{K_{d}\left(e^{-\alpha f\Delta \psi }+e^{\left(1-\alpha \right)f\Delta \psi }\right)+\left({\left[K^{+}\right]}_{in}+{\left[K^{+}\right]}_{out}\right)}\cdot \frac{1}{1+{\left(10^{pH-pK_{a}}\right)}^{2}}$$

Figure S2 plots KcsA activity for symmetric $\left[K^{+}\right]$. In Figure S2, cytosolic pH is held constant at 3.0 (S2A) or is varied (S2B). At low cytosolic pH, KcsA activity exactly matches that of the parent AD model. However, as cytosolic pH increases (S2B), $K^{+}$ transport rate decreases regardless of direction, such that even a large Δψ cannot drive $K^{+}$ transport. This is consistent with literature measurements of KcsA activity at symmetric $\left[K^{+}\right]$.^47^ The AD model used for (32) can be applied to other secondary transport processes,^12^^,^^121^ and can be similarly modified to include gating or inhibition.

#### Metabolism

We base our metabolic model on homolactic fermentation, where glucose is converted to lactate without respiration. In homolactic fermentation, NADH does not transport charge across the cell membrane, as no respiration occurs. The stoichiometry for this metabolism is given in 33; here, one mole of external glucose is converted to two moles of ATP and two moles of internal lactic acid, with a net-zero NADH balance.^122^^,^^123^ In (34), extracellular glucose, ATP, and extracellular ammonium are converted to biomass, with stoichiometry from literature estimates.^124^^,^^125^ Ammonium is included due to its pH-buffering and ionic strength effects.

(Equation 33) $$Glucose+2ADP+2Pi\to 2Lactate+2H^{+}+2ATP$$

(Equation 34) $$Glucose+\frac{X_{glu}}{Y_{ATP}^{\max}}ATP+\frac{X_{glu}}{Y_{NH3}}NH_{4}^{+}\to X_{glu}\cdot Biomass$$

Rates of lactate and biomass formation are based on multi-substrate Monod without substrate inhibition,^126^ and are given as Equations 35 and 36 respectively.

(Equation 35) $$r_{metabolism}=V_{\max\;1}\cdot \frac{\left[Glu_{out}\right]}{K_{m,Glu}+\left[Glu_{out}\right]}\cdot \frac{\left[ADP\right]}{K_{m,ADP}+\left[ADP\right]}\cdot \frac{\left[Pi\right]}{K_{m,Pi}+\left[Pi\right]}$$

(Equation 36) $$r_{biomass}=V_{\max\;2}\cdot \frac{\left[Glu_{out}\right]}{K_{m,Glu}+\left[Glu_{out}\right]}\cdot \frac{\left[ATP\right]}{K_{m,ATP}+\left[ATP\right]}\cdot \frac{\left[NH_{3}\right]}{K_{m,NH_{3}}+\left[NH_{3}\right]}$$

ATP is also consumed by cell maintenance, as given by Equation 37.

(Equation 37) $$r_{maintain}=\mu_{maintain}\cdot \frac{\left[ATP\right]}{K_{maintain,ATP}+\left[ATP\right]}$$

We further include metabolic and growth inhibition due to low internal pH, given in Equations 38 and 39, using the form developed by Han and Levenspiel for noncompetitive inhibition.^127^ As internal pH decreases, both metabolic and growth rates decrease,^73^^,^^128^ but growth ceases before metabolism.^128^ Inhibition at low $pH_{in}$ is omitted for (37) as the fraction of ATP consumed for maintenance is expected to increase with cell stress.^129^ Minimum cytosolic pH values are largely organism-dependent, but viability at internal pH as low as 4.5-5 has been reported for lactic-acid bacteria.^73^^,^^74^

(Equation 38) $$r_{metabolism}=V_{\max\;1}\cdot \frac{\left[Glu_{out}\right]}{K_{m,Glu}+\left[Glu_{out}\right]}\cdot \frac{\left[ADP\right]}{K_{m,ADP}+\left[ADP\right]}\cdot \frac{\left[Pi\right]}{K_{m,Pi}+\left[Pi\right]}\cdot \left(1-10^{pH_{in}-pH_{\lim,metabolism}}\right)$$

(Equation 39) $$r_{biomass}=V_{\max\;2}\cdot \frac{\left[Glu_{out}\right]}{K_{m,Glu}+\left[Glu_{out}\right]}\cdot \frac{\left[ATP\right]}{K_{m,ATP}+\left[ATP\right]}\cdot \frac{\left[NH_{3}\right]}{K_{m,NH_{3}}+\left[NH_{3}\right]}\cdot \left(1-10^{pH_{in}-pH_{\lim,growth}}\right)$$

The lactic acid produced at a rate according to (38) acidifies the cytosol; $pH_{in}$ is maintained by $H^{+}$ efflux through F-ATPase, while Δψ is maintained by the combined activities of F-ATPase and KcsA. The kinetics of F-ATPase and KcsA depend, in part, on $pH_{in}$ and Δψ. With these two components, we model the dynamics of the cytosolic pH and the transmembrane potential. The kinetic expressions for membrane potential (Equation 2), lactic acid diffusion (Equation 5), F-ATPase (Equation 27), KcsA (Equation 32), metabolism (Equation 38), cell maintenance (Equation 37), and biomass formation (Equation 39) form a system of ODEs, which we solve numerically with ode15s. We first consider F-ATPase in isolation, then include KcsA, and finally integrate both proteins into a whole-cell metabolic model. For simulated cell response and fermentation, the model is capable of several modes: constant ATP, constant ATP production, and batch fermentation.

#### Energy pool calculations

Over the course of the fermentation, we calculate the specific and total energy available from charge transport (Δψ), ATP hydrolysis, and ion transport ($\Delta \left[H^{+}\right]$, $\Delta \left[K^{+}\right]$). For charge transport, total available energy is stored as a capacitor, and is given as (40), where *C* is the cell membrane capacitance.

(Equation 40) $$U_{\Delta \psi }=\frac{1}{2}C{\left(\Delta \psi \right)}^{2}$$

For ATP hydrolysis and ion transport, we first calculate the specific free energy according to (41), where ΔG is the free energy at nonstandard conditions, $\Delta G^{\circ }$ is the free energy at standard conditions, and *Q* is the reaction quotient.

(Equation 41) $$\Delta G=\Delta G^{\circ }+RTln\left(Q\right)$$

The free energy of ATP hydrolysis, therefore, is given as (42).

(Equation 42) $$\Delta G_{ATP}=\Delta G_{ATP}^{\circ }+RTln\left(\frac{\left[ADP\right]\left[Pi\right]}{\left[ATP\right]}\right)$$

To calculate the total energy stored as ATP, we integrate the specific energy of hydrolysis over the concentration of ATP, assuming complete hydrolysis. We define ϵ as the concentration of ATP that has been hydrolyzed. In that case, we find from Equation 42 that the total energy available for ATP hydrolysis (per cell) is given by Equation 43:

(Equation 43) $$U_{ATP}=V_{cell}\cdot \int_{0}^{\left[ATP\right]}\left(\Delta G_{ATP}^{\circ }+RTln\left(\frac{\left(\left[ADP\right]+\epsilon \right)\left(\left[Pi\right]+\epsilon \right)}{\left[ATP\right]-\epsilon }\right)\right)d\epsilon$$

For ionic transport, we first calculate the specific free energy of transporting ions out of the cell. Here, the standard free energy is zero, yielding Equation 44, where $M^{+}$ is $H^{+}$ or $K^{+}$.

(Equation 44) $$\Delta G_{M^{+}}=RTln\left(\frac{{\left[M^{+}\right]}_{out}}{{\left[M^{+}\right]}_{in}}\right)$$

We can calculate the specific energy of ion transport of both $H^{+}$ and $K^{+}$ using (44). For $K^{+}$, the total energy for ion transport is given by Equation 45, assuming constant extracellular $\left[K^{+}\right]$:

(Equation 45) $$U_{K^{+}}=V_{cell}\cdot \int_{{\left[K^{+}\right]}_{in}}^{{\left[K^{+}\right]}_{out}}RTln\left(\frac{{\left[K^{+}\right]}_{out}}{\left[K^{+}\right]}\right)d\left[K^{+}\right]$$

In the case of $H^{+}$, equillibrium is reached when the cytosolic pH and extracellular pH are equal. However, only a small fraction of $H^{+}$ is available as free protons,^1^ with the rest bound to phosphate or other intracellular buffers. If we consider the buffer capacity of the cytosol to provide a source of $H^{+}$, we can calculate the total energy available through $H^{+}$ transport. The buffer capacity β is calculated using the pHtools toolkit and is given by Equation 46, where *M* is the concentration of acid or base:

(Equation 46) $$\beta \left(pH\right)=\frac{\Delta M}{\Delta pH}$$

Therefore, integrating to calculate the total energy, we find:

(Equation 47) $$U_{H^{+}}=V_{cell}\cdot 2.303RT\int_{pH_{in}}^{pH_{out}}\left(\beta \left(pH\right)\cdot \left(pH-pH_{out}\right)\right)dpH$$

From (47) and (45), we can calculate the total energy available from ion transport (referred to in the text as “energy pools”) for both ΔpH and $\Delta \left[K^{+}\right]$.

## Data Availability

This paper analyzes existing, publicly available data. The references for the data are listed in Table S1. All original code is available in the supplemental information. Any additional information required to reanalyze the data reported in this paper is available from the lead contact upon request.

## Supplemental information

Document S1. Figures S1–S4 and Table S1

Data S1. Source code used to generate Figures 1–3

## References

[1] Benarroch J.M., Asally M. (2020). The microbiologists guide to membrane potential dynamics. Trends Microbiol..

[2] Schofield Z., Meloni G.N., Tran P., Zerfass C., Sena G., Hayashi Y., Grant M., Contera S.A., Minteer S.D., Kim M. (2020). Bioelectrical understanding and engineering of cell biology. J. R. Soc. Interface.

[3] Selberg J., Gomez M., Rolandi M. (2018). The potential for convergence between synthetic biology and bioelectronics. Cell Syst..

[4] Albers S.-V., Van de Vossenberg J.L., Driessen A.J., Konings W.N. (2001). Bioenergetics and solute uptake under extreme conditions. Extremophiles.

[5] Calisto F., Sousa F.M., Sena F.V., Refojo P.N., Pereira M.M. (2021). Mechanisms of energy transduction by charge translocating membrane proteins. Chem. Rev..

[6] Darbani B., Kell D.B., Borodina I. (2018). Energetic evolution of cellular transportomes. BMC Genom..

[7] Brown I.I., Galperin M.Y., Glagolev A.N., Skulachev V.P. (1983). Utilization of energy stored in the form of na+ and k+ ion gradients by bacterial cells. Eur. J. Biochem..

[8] Papadimitriou K., Alegría Á., Bron P.A., De Angelis M., Gobbetti M., Kleerebezem M., Lemos J.A., Linares D.M., Ross P., Stanton C. (2016). Stress physiology of lactic acid bacteria. Microbiol. Mol. Biol. Rev..

[9] Konings W.N., Poolman B., van Veen H.W. (1994). Solute transport and energy transduction in bacteria. Antonie Leeuwenhoek.

[10] Konings W.N., Lolkema J.S., Bolhuis H., van Veen H.W., Poolman B., Driessen A.J. (1997). The role of transport processes in survival of lactic acid bacteria, energy transduction and multidrug resistance. Antonie Leeuwenhoek.

[11] Beagle S.D., Lockless S.W. (2021). Unappreciated roles for k+ channels in bacterial physiology. Trends Microbiol..

[12] Nelson P.H. (2011). A permeation theory for single-file ion channels: One-and two-step models. J. Chem. Phys..

[13] Bakker E.P., Mangerich W.E. (1981). Interconversion of components of the bacterial proton motive force by electrogenic potassium transport. J. Bacteriol..

[14] Mancini L., Terradot G., Tian T., Pu Y., Li Y., Lo C.-J., Bai F., Pilizota T. (2020). A general workflow for characterization of nernstian dyes and their effects on bacterial physiology. Biophys. J..

[15] Brown G.C. (1992). The leaks and slips of bioenergetic membranes. Faseb. J..

[16] White D., Drummond J., Fuqua C. (2012).

[17] Abee T., Hellingwerf K.J., Konings W.N. (1988). Effects of potassium ions on proton motive force in rhodobacter sphaeroides. J. Bacteriol..

[18] Mayer A., Weuster-Botz D. (2017). Reaction engineering analysis of the autotrophic energy metabolism of clostridium aceticum. FEMS Microbiol. Lett..

[19] Kashket E.R. (1987). Bioenergetics of lactic acid bacteria: cytoplasmic ph and osmotolerance. FEMS Microbiol. Lett..

[20] Liu J., Martinez-Corral R., Prindle A., Lee D.-y. D., Larkin J., Gabalda-Sagarra M., Garcia-Ojalvo J., Süel G.M. (2017). Coupling between distant biofilms and emergence of nutrient time-sharing. Science.

[21] Pandey R., Vischer N.O.E., Smelt J.P.P.M., van Beilen J.W.A., Ter Beek A., De Vos W.H., Brul S., Manders E.M.M. (2016). Intracellular ph response to weak acid stress in individual vegetative bacillus subtilis cells. Appl. Environ. Microbiol..

[22] Prindle A., Liu J., Asally M., Ly S., Garcia-Ojalvo J., Süel G.M. (2015). Ion channels enable electrical communication in bacterial communities. Nature.

[23] Fritts R.K., Bird J.T., Behringer M.G., Lipzen A., Martin J., Lynch M., McKinlay J.B. (2020). Enhanced nutrient uptake is sufficient to drive emergent cross-feeding between bacteria in a synthetic community. ISME J..

[24] Flemming H.-C., Wingender J., Szewzyk U., Steinberg P., Rice S.A., Kjelleberg S. (2016). Biofilms: an emergent form of bacterial life. Nat. Rev. Microbiol..

[25] Horaruang W., Hills A., Blatt M.R. (2020). Communication between the plasma membrane and tonoplast is an emergent property of ion transport. Plant Physiol..

[26] Levin M., Martyniuk C.J. (2018). The bioelectric code: An ancient computational medium for dynamic control of growth and form. Biosystems.

[27] Yang C.-Y., Bialecka-Fornal M., Weatherwax C., Larkin J.W., Prindle A., Liu J., Garcia-Ojalvo J., Süel G.M. (2020). Encoding membrane-potential-based memory within a microbial community. Cell Syst..

[28] Grobas I., Bazzoli D.G., Asally M. (2020). Biofilm and swarming emergent behaviours controlled through the aid of biophysical understanding and tools. Biochem. Soc. Trans..

[29] Bruni G.N., Kralj J.M. (2020). Membrane voltage dysregulation driven by metabolic dysfunction underlies bactericidal activity of aminoglycosides. Elife.

[30] Galera-Laporta L., Comerci C.J., Garcia-Ojalvo J., Süel G.M. (2021). Ionobiology: The functional dynamics of the intracellular metallome, with lessons from bacteria. Cell Syst..

[31] Hodgkin A.L., Huxley A.F. (1952). A quantitative description of membrane current and its application to conduction and excitation in nerve. J. Physiol..

[32] Turina P., Petersen J., Gräber P. (2016). Thermodynamics of proton transport coupled atp synthesis. Biochim. Biophys. Acta.

[33] Cordero-Morales J.F., Cuello L.G., Perozo E. (2006). Voltage-dependent gating at the kcsa selectivity filter. Nat. Struct. Mol. Biol..

[34] Senger R.S., Papoutsakis E.T. (2008). Genome-scale model for clostridium acetobutylicum: Part i. metabolic network resolution and analysis. Biotechnol. Bioeng..

[35] Liao C., Seo S.-O., Celik V., Liu H., Kong W., Wang Y., Blaschek H., Jin Y.-S., Lu T. (2015). Integrated, systems metabolic picture of acetone-butanol-ethanol fermentation by clostridium acetobutylicum. Proc. Natl. Acad. Sci. USA.

[36] Gilbert D., Heiner M., Jayaweera Y., Rohr C. (2019). Towards dynamic genome-scale models. Briefings Bioinf..

[37] Russell J. (1992). Another explanation for the toxicity of fermentation acids at low ph: anion accumulation versus uncoupling. J. Appl. Bacteriol..

[38] Kroll R.G., Booth I.R. (1983). The relationship between intracellular ph, the ph gradient and potassium transport in escherichia coli. Biochem. J..

[39] Humphries J., Xiong L., Liu J., Prindle A., Yuan F., Arjes H.A., Tsimring L., Süel G.M. (2017). Species-independent attraction to biofilms through electrical signaling. Cell.

[40] Kakinuma Y. (1998). Inorganic cation transport and energy transduction in enterococcus hirae and other streptococci. Microbiol. Mol. Biol. Rev..

[41] Ochrombel I., Ott L., Krämer R., Burkovski A., Marin K. (2011). Impact of improved potassium accumulation on ph homeostasis, membrane potential adjustment and survival of corynebacterium glutamicum. Biochim. Biophys. Acta.

[42] Huang L., Gibbins L.N., Forsberg C.W. (1985). Transmembrane ph gradient and membrane potential in clostridium acetobutylicum during growth under acetogenic and solventogenic conditions. Appl. Environ. Microbiol..

[43] Gries C.M., Sadykov M.R., Bulock L.L., Chaudhari S.S., Thomas V.C., Bose J.L., Bayles K.W. (2016). Potassium uptake modulates staphylococcus aureus metabolism. mSphere.

[44] Kashket E.R., Barker S.L. (1977). Effects of potassium ions on the electrical and ph gradients across the membrane of streptococcus lactis cells. J. Bacteriol..

[45] Poolman B., Hellingwerf K.J., Konings W.N. (1987). Regulation of the glutamate-glutamine transport system by intracellular ph in streptococcus lactis. J. Bacteriol..

[46] Bernèche S., Roux B. (2003). A microscopic view of ion conduction through the k+ channel. Proc. Natl. Acad. Sci. USA.

[47] Zakharian E., Reusch R.N. (2004). Streptomyces lividans potassium channel kcsa is regulated by the potassium electrochemical gradient. Biochem. Biophys. Res. Commun..

[48] Chakrapani S., Cordero-Morales J.F., Perozo E. (2007). A quantitative description of kcsa gating i: macroscopic currents. J. Gen. Physiol..

[49] Gao L., Mi X., Paajanen V., Wang K., Fan Z. (2005). Activation-coupled inactivation in the bacterial potassium channel kcsa. Proc. Natl. Acad. Sci. USA.

[50] Neves A.R., Ramos A., Costa H., van Swam I.I., Hugenholtz J., Kleerebezem M., de Vos W., Santos H. (2002). Effect of different nadh oxidase levels on glucose metabolism by lactococcus lactis: kinetics of intracellular metabolite pools determined by in vivo nuclear magnetic resonance. Appl. Environ. Microbiol..

[51] Sun Y. (2016).

[52] Guan N., Liu L. (2020). Microbial response to acid stress: mechanisms and applications. Appl. Microbiol. Biotechnol..

[53] Wu C., Zhang J., Chen W., Wang M., Du G., Chen J. (2012). A combined physiological and proteomic approach to reveal lactic-acid-induced alterations in lactobacillus casei zhang and its mutant with enhanced lactic acid tolerance. Appl. Microbiol. Biotechnol..

[54] Cotter P.D., Hill C. (2003). Surviving the acid test: responses of gram-positive bacteria to low ph. Microbiol. Mol. Biol. Rev..

[55] Lorca G.L., Font de Valdez G. (2001). Acid tolerance mediated by membrane atpases in lactobacillus acidophilus. Biotechnol. Lett..

[56] Carvalho A.L., Turner D.L., Fonseca L.L., Solopova A., Catarino T., Kuipers O.P., Voit E.O., Neves A.R., Santos H. (2013). Metabolic and transcriptional analysis of acid stress in lactococcus lactis, with a focus on the kinetics of lactic acid pools. PLoS One.

[57] Alexander B., Leach S., Ingledew W.J. (1987). The relationship between chemiosmotic parameters and sensitivity to anions and organic acids in the acidophile thiobacillus ferrooxidans. Microbiology.

[58] Yasuda R., Noji H., Yoshida M., Kinosita K., Itoh H. (2001). Resolution of distinct rotational substeps by submillisecond kinetic analysis of f1-atpase. Nature.

[59] Gao Y.Q., Yang W., Karplus M. (2005). A structure-based model for the synthesis and hydrolysis of atp by f1-atpase. Cell.

[60] Adachi K., Oiwa K., Nishizaka T., Furuike S., Noji H., Itoh H., Yoshida M., Kinosita K. (2007). Coupling of rotation and catalysis in f1-atpase revealed by single-molecule imaging and manipulation. Cell.

[61] Nesci S., Trombetti F., Ventrella V., Pagliarani A. (2016). The c-ring of the f1fo-atp synthase: facts and perspectives. J. Membr. Biol..

[62] Pogoryelov D., Klyszejko A.L., Krasnoselska G.O., Heller E.-M., Leone V., Langer J.D., Vonck J., Müller D.J., Faraldo-Gómez J.D., Meier T. (2012). Engineering rotor ring stoichiometries in the atp synthase. Proc. Natl. Acad. Sci. USA.

[63] Nirody J.A., Budin I., Rangamani P. (2020). Atp synthase: Evolution, energetics, and membrane interactions. J. Gen. Physiol..

[64] Krebstakies T., Aldag I., Altendorf K., Greie J.-C., Deckers-Hebestreit G. (2008). The stoichiometry of subunit c of escherichia coli atp synthase is independent of its rate of synthesis. Biochemistry.

[65] Ballhausen B., Altendorf K., Deckers-Hebestreit G. (2009). Constant c 10 ring stoichiometry in the escherichia coli atp synthase analyzed by cross-linking. J. Bacteriol..

[66] Silverstein T.P. (2014). An exploration of how the thermodynamic efficiency of bioenergetic membrane systems varies with c-subunit stoichiometry of f1f0 atp synthases. J. Bioenerg. Biomembr..

[67] Poolman B., Molenaar D., Smid E.J., Ubbink T., Abee T., Renault P.P., Konings W.N. (1991). Malolactic fermentation: electrogenic malate uptake and malate/lactate antiport generate metabolic energy. J. Bacteriol..

[68] Zhang H., Pan Y., Hu L., Hudson M.A., Hofstetter K.S., Xu Z., Rong M., Wang Z., Prasad B.V.V., Lockless S.W. (2020). Trka undergoes a tetramer-to-dimer conversion to open trkh which enables changes in membrane potential. Nat. Commun..

[69] Roe A.J., McLaggan D., O‘Byrne C.P., Booth I.R. (2000). Rapid inactivation of the escherichia coli kdp k+ uptake system by high potassium concentrations. Mol. Microbiol..

[70] Nelson P.H. (2003). A permeation theory for single-file ion channels: Concerted-association/dissociation. J. Chem. Phys..

[71] Nelson P.H. (2002). A permeation theory for single-file ion channels: Corresponding occupancy states produce michaelis–menten behavior. J. Chem. Phys..

[72] Stautz J., Hellmich Y., Fuss M.F., Silberberg J.M., Devlin J.R., Stockbridge R.B., Hänelt I. (2021). Molecular mechanisms for bacterial potassium homeostasis. J. Mol. Biol..

[73] Booth I.R. (1985). Regulation of cytoplasmic ph in bacteria. Microbiol. Rev..

[74] Nannen N.L., Hutkins R.W. (1991). Intracellular ph effects in lactic acid bacteria. J. Dairy Sci..

[75] Hutkins R.W., Nannen N.L. (1993). Ph homeostasis in lactic acid bacteria. J. Dairy Sci..

[76] Kashket E.R., Blanchard A.G., Metzger W.C. (1980). Proton motive force during growth of streptococcus lactis cells. J. Bacteriol..

[77] Bonneau R., Facciotti M.T., Reiss D.J., Schmid A.K., Pan M., Kaur A., Thorsson V., Shannon P., Johnson M.H., Bare J.C. (2007). A predictive model for transcriptional control of physiology in a free living cell. Cell.

[78] Van Santen J.A., Kautsar S.A., Medema M.H., Linington R.G. (2021). Microbial natural product databases: moving forward in the multi-omics era. Nat. Prod. Rep..

[79] Karlsen S.T., Vesth T.C., Oregaard G., Poulsen V.K., Lund O., Henderson G., Bælum J. (2021). Machine learning predicts and provides insights into milk acidification rates of lactococcus lactis. PLoS One.

[80] Slonczewski J.L., Fujisawa M., Dopson M., Krulwich T.A. (2009). Cytoplasmic ph measurement and homeostasis in bacteria and archaea. Adv. Microb. Physiol..

[81] Arce-Rodríguez A., Volke D.C., Bense S., Häussler S., Nikel P.I. (2019). Non-invasive, ratiometric determination of intracellular ph in pseudomonas species using a novel genetically encoded indicator. Microb. Biotechnol..

[82] Comerci C.J., Gillman A.L., Galera-Laporta L., Gutierrez E., Groisman A., Larkin J.W., Garcia-Ojalvo J., Süel G.M. (2022). Localized electrical stimulation triggers cell-type-specific proliferation in biofilms. Cell Syst..

[83] Sirec T., Benarroch J.M., Buffard P., Garcia-Ojalvo J., Asally M. (2019). Electrical polarization enables integrative quality control during bacterial differentiation into spores. iScience.

[84] Follmann M., Becker M., Ochrombel I., Ott V., Krämer R., Marin K. (2009). Potassium transport in corynebacterium glutamicum is facilitated by the putative channel protein cglk, which is essential for ph homeostasis and growth at acidic ph. J. Bacteriol..

[85] Thompson A.N., Posson D.J., Parsa P.V., Nimigean C.M. (2008). Molecular mechanism of ph sensing in kcsa potassium channels. Proc. Natl. Acad. Sci. USA.

[86] Rodriguez-Verdugo A. (2021). Evolving interactions and emergent functions in microbial consortia. mSystems.

[87] Schwalm N.D., Mojadedi W., Gerlach E.S., Benyamin M., Perisin M.A., Akingbade K.L. (2019). Developing a microbial consortium for enhanced metabolite production from simulated food waste. Fermentation.

[88] Brown J.L., Perisin M.A., Swift C.L., Benyamin M., Liu S., Singan V., Zhang Y., Savage E., Pennacchio C., Grigoriev I.V., O'Malley M.A. (2022). Co-cultivation of anaerobic fungi with clostridium acetobutylicum bolsters butyrate and butanol production from cellulose and lignocellulose. J. Ind. Microbiol. Biotechnol..

[89] Lovley D.R., Holmes D.E. (2022). Electromicrobiology: the ecophysiology of phylogenetically diverse electroactive microorganisms. Nat. Rev. Microbiol..

[90] Zhang J., Wu C., Du G., Chen J. (2012). Enhanced acid tolerance in lactobacillus casei by adaptive evolution and compared stress response during acid stress. Biotechnol. Bioproc. Eng..

[91] Richard H., Foster J.W. (2004). Escherichia coli glutamate-and arginine-dependent acid resistance systems increase internal ph and reverse transmembrane potential. J. Bacteriol..

[92] O’Sullivan E., Condon S. (1997). Intracellular ph is a major factor in the induction of tolerance to acid and other stresses in lactococcus lactis. Appl. Environ. Microbiol..

[93] Zhu Z., Yang P., Wu Z., Zhang J., Du G. (2019). Systemic understanding of lactococcus lactis response to acid stress using transcriptomics approaches. J. Ind. Microbiol. Biotechnol..

[94] Goch W., Bal W. (2020). Stochastic or not? method to predict and quantify the stochastic effects on the association reaction equilibria in nanoscopic systems. J. Phys. Chem. A.

[95] Konings W. (2002). Lactic Acid Bacteria: Genetics, Metabolism and Applications.

[96] Paternò G.M., Bondelli G., Lanzani G. (2021). Bringing microbiology to light: Toward all-optical electrophysiology in bacteria. Bioelectricity.

[97] Roy D., Shapira Z., Weiss S. (2022). Membrane potential sensing: Material design and method development for single particle optical electrophysiology. J. Chem. Phys..

[98] Bakker E.P., Harold F.M. (1980). Energy coupling to potassium transport in streptococcus faecalis. interplay of atp and the protonmotive force. J. Biol. Chem..

[99] Quintana I.M., Gibhardt J., Turdiev A., Hammer E., Commichau F.M., Lee V.T., Magni C., Stülke J. (2019). The kupa and kupb proteins of lactococcus lactis il1403 are novel c-di-amp receptor proteins responsible for potassium uptake. J. Bacteriol..

[100] Fuss M.F., Wieferig J.-P., Corey R.A., Hellmich Y., Tascón I., Sousa J.S., Stansfeld P.J., Vonck J., Hänelt I. (2023). Cyclic di-amp traps proton-coupled k+ transporters of the kup family in an inward-occluded conformation. bioRxiv.

[101] Bowman L., Zeden M.S., Schuster C.F., Kaever V., Gründling A. (2016). New insights into the cyclic di-adenosine monophosphate (c-di-amp) degradation pathway and the requirement of the cyclic dinucleotide for acid stress resistance in staphylococcus aureus. J. Biol. Chem..

[102] Trchounian A., Kobayashi H. (1999). Kup is the major k+ uptake system in escherichia coli upon hyper-osmotic stress at a low ph. FEBS Lett..

[103] Pham H.T., Nhiep N.T.H., Vu T.N.M., Huynh T.N., Zhu Y., Huynh A.L.D., Chakrabortti A., Marcellin E., Lo R., Howard C.B. (2018). Enhanced uptake of potassium or glycine betaine or export of cyclic-di-amp restores osmoresistance in a high cyclic-di-amp lactococcus lactis mutant. PLoS Genet..

[104] Herbold C.W., Lehtovirta-Morley L.E., Jung M.-Y., Jehmlich N., Hausmann B., Han P., Loy A., Pester M., Sayavedra-Soto L.A., Rhee S.-K. (2017). Ammonia-oxidising archaea living at low ph: insights from comparative genomics. Environ. Microbiol..

[105] Walker G.M., Basso T.O. (2020). Mitigating stress in industrial yeasts. Fungal Biol..

[106] Jahnke J.P., Mackie D.M., Benyamin M., Ganguli R., Sumner J.J. (2015).

[107] Jahnke J.P., Benyamin M.S., Sumner J.J., Mackie D.M. (2016). Using reverse osmosis membranes to couple direct ethanol fuel cells with ongoing fermentations. Ind. Eng. Chem. Res..

[108] Benyamin M.S., Jahnke J.P., Mackie D.M. (2017). Vapor-fed bio-hybrid fuel cell. Biotechnol. Biofuels.

[109] Guaragnella N., Bettiga M. (2021). Acetic acid stress in budding yeast: From molecular mechanisms to applications. Yeast.

[110] Peetermans A., Foulquié-Moreno M.R., Thevelein J.M. (2021). Mechanisms underlying lactic acid tolerance and its influence on lactic acid production in saccharomyces cerevisiae. Microb. Cell.

[111] Dougherty D.P., Da Conceicao Neta E.R., McFeeters R.F., Lubkin S.R., Breidt F. (2006). Semi-mechanistic partial buffer approach to modeling ph, the buffer properties, and the distribution of ionic species in complex solutions. J. Agric. Food Chem..

[112] Thomas K.C., Hynes S.H., Ingledew W.M. (2002). Influence of medium buffering capacity on inhibition of saccharomyces cerevisiae growth by acetic and lactic acids. Appl. Environ. Microbiol..

[113] Junge W., Sielaff H., Engelbrecht S. (2009). Torque generation and elastic power transmission in the rotary fof1-atpase. Nature.

[114] Okuno D., Iino R., Noji H. (2011). Rotation and structure of f o f 1-atp synthase. J. Biochem..

[115] Okazaki K.-i., Hummer G. (2013). Phosphate release coupled to rotary motion of f1-atpase. Proc. Natl. Acad. Sci. USA.

[116] Gao Y.Q., Yang W., Marcus R.A., Karplus M. (2003). A model for the cooperative free energy transduction and kinetics of atp hydrolysis by f1-atpase. Proc. Natl. Acad. Sci. USA.

[117] Mukherjee S., Warshel A. (2011). Electrostatic origin of the mechanochemical rotary mechanism and the catalytic dwell of f1-atpase. Proc. Natl. Acad. Sci. USA.

[118] Shu Y.-G., Lai P.-Y. (2008). Systematic kinetics study of fof1-atpase: analytic results and comparison with experiments. J. Phys. Chem. B.

[119] Noskov S.Y., Bernèche S., Roux B. (2004). Control of ion selectivity in potassium channels by electrostatic and dynamic properties of carbonyl ligands. Nature.

[120] Gibby W., Luchinsky D., Kaufman I.K., Ward A., McClintock P. (2017). 2017 International Conference on Noise and Fluctuations (ICNF).

[121] Carvalho-de Souza J.L., Saponaro A., Bassetto C., Rauh O., Schroeder I., Franciolini F., Catacuzzeno L., Bezanilla F., Thiel G., Moroni A. (2022). Experimental challenges in ion channel research: Uncovering basic principles of permeation and gating in potassium channels. Adv. Phys. X.

[122] Gänzle M.G. (2015). Lactic metabolism revisited: metabolism of lactic acid bacteria in food fermentations and food spoilage. Curr. Opin. Food Sci..

[123] Wang Y., Wu J., Lv M., Shao Z., Hungwe M., Wang J., Bai X., Xie J., Wang Y., Geng W. (2021). Metabolism characteristics of lactic acid bacteria and the expanding applications in food industry. Front. Bioeng. Biotechnol..

[124] Papoutsakis E.T. (2000). Equations and calculations for fermentations of butyric acid bacteria. Biotechnol. Bioeng..

[125] Stouthamer A.H., Bettenhaussen C. (1973). Utilization of energy for growth and maintenance in continuous and batch cultures of microorganisms: A reevaluation of the method for the determination of atp production by measuring molar growth yields. Biochim. Biophys. Acta.

[126] Şeker Ş., Beyenal H., Salih B., Tanyolaç A. (1997). Multi-substrate growth kinetics of pseudomonas putida for phenol removal. Appl. Microbiol. Biotechnol..

[127] Han K., Levenspiel O. (1988). Extended monod kinetics for substrate, product, and cell inhibition. Biotechnol. Bioeng..

[128] Even S., Lindley N.D., Cocaign-Bousquet M. (2003). Transcriptional, translational and metabolic regulation of glycolysis in lactococcus lactis subsp. cremoris mg 1363 grown in continuous acidic cultures. Microbiology.

[129] Deng Y., Beahm D.R., Ionov S., Sarpeshkar R. (2021). Measuring and modeling energy and power consumption in living microbial cells with a synthetic atp reporter. BMC Biol..

