# Supplementary material for: Modeling control and transduction of electrochemical gradients in acid-stressed bacteria
Source: iScience. 2023 Jun 17;26(7):107140. doi: 10.1016/j.isci.2023.107140 (PMC10316662; doi:10.1016/j.isci.2023.107140)
Supplement: Document S1. Figures S1–S4 and Table S1 [file mmc1.pdf]

iScience, Volume *26*

Supplemental information

Modeling control and transduction of electrochemical gradients in acid-stressed bacteria

Marcus S. Benyamin, Matthew P. Perisin, Caleb A. Hellman, Nathan D. Schwalm III, Justin P. Jahnke, and Christian J. Sund

Supplemental

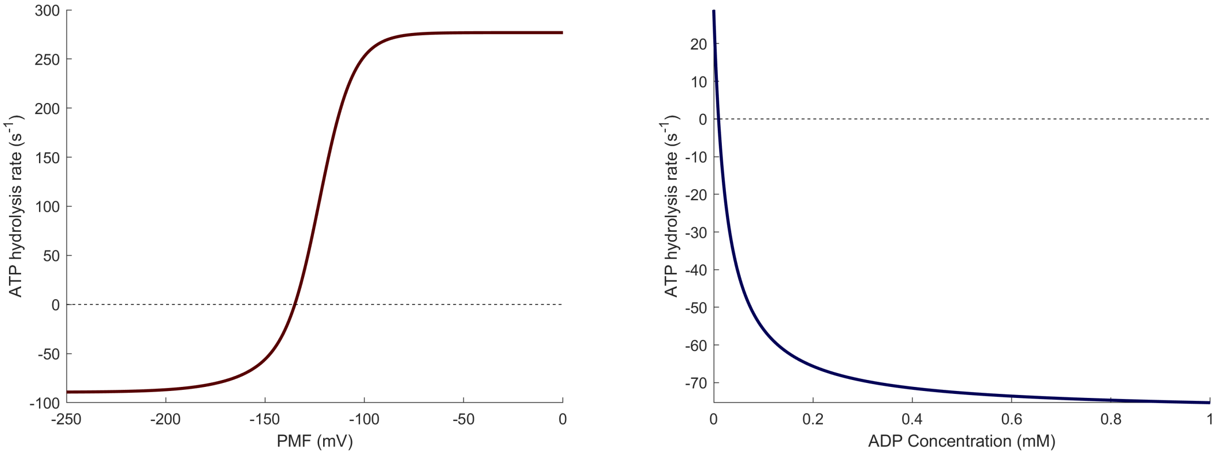

(A) (B)

Figure S1, Supplemental: F-ATPase kinetics. This figure supports Figures 1, 2, and 3 in the main text.
(A) F-ATPase kinetics with varied PMF at 1 mM ATP, 0.1 mM ADP, and 10 mM Pi.
(B) F-ATPase kinetics with varied ADP concentration at 1 mM ATP, 10 mM Pi, and $\Delta\psi = -150$ mV.

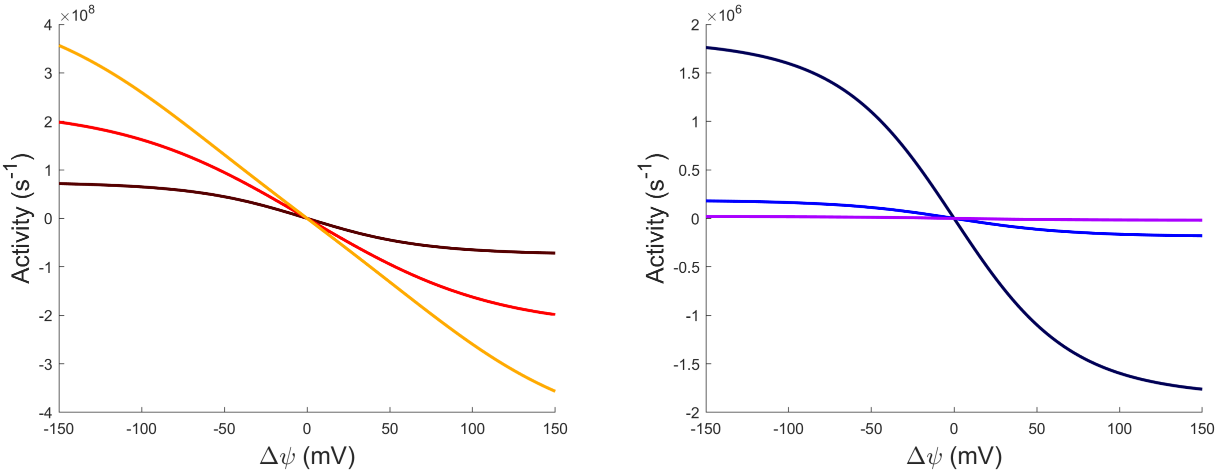

(A) (B)

Figure S2, supplemental: KcsA kinetics. This figure supports Figures 2 and 3 in the main text.
(A) KcsA K^+ transport rate for different symmetrical K^+ concentrations, at $pH_{in} = 3.0$: $[K^+]$ is 0.25 M (brown), 0.75 M (red), and 1.5 M (gold)
(B) KcsA K^+ transport rate for different cytosolic pH, at a symmetrical K^+ concentration of 0.25 M: pH_{in} is 5.0 (navy), 5.5 (blue), or 6.0 (purple).

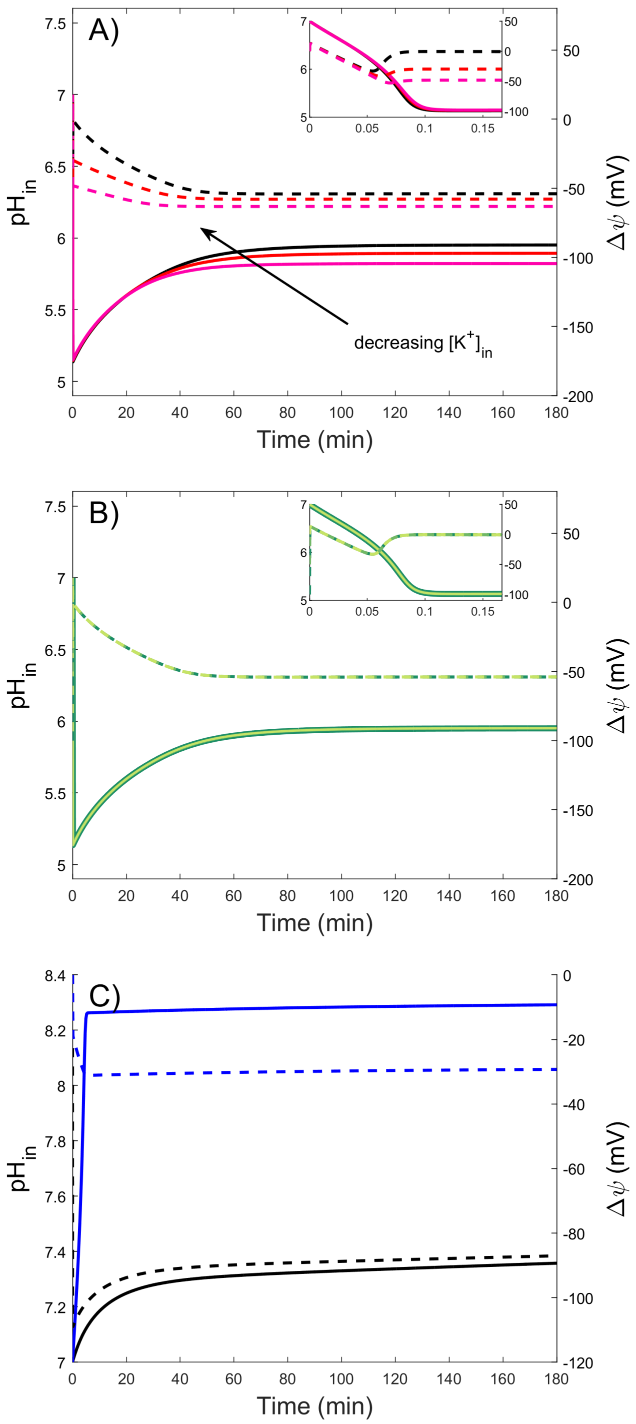

Figure S3, supplemental: Two-transporter model system behavior under different initial conditions or in the absence of acid stress, supporting Figure 2 in the main text. Cytosolic pH (left axis, solid lines) and $\Delta\psi$ (right axis, dashed lines) are shown.

(A) Sensitivity analysis of the acid stress response to initial intracellular potassium concentration. Simulation results are shown for initial concentrations of 50 mM (black), 150 mM (red), and 300 mM (pink).

(B) Sensitivity analysis of the acid stress response to initial membrane potential. Simulation results are shown for initial concentrations of 0 mV (dark green), -50 mV (green), and -100 mV (yellow-green).

(C) Two-transporter model system in the absence of acid stress. Here, KcsA is pH-gated (black) or ungated (blue). The external pH is set to 7.

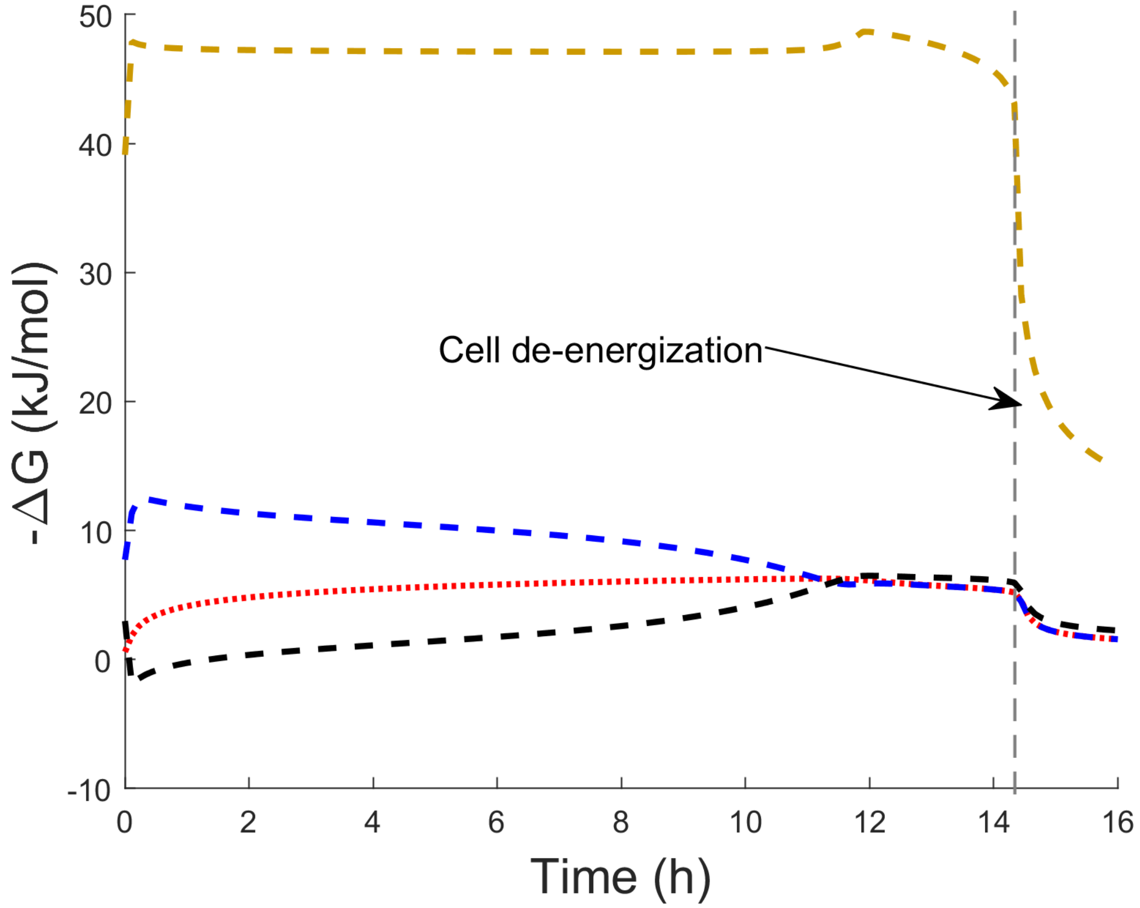

Figure S4, supplemental: Specific energies of energy pool contributors during lactic acid fermentation, supporting Figure 3 in the main text. Specific energy is given for ATP hydrolysis and for ion transport along each gradient. Shown: ATP hydrolysis (gold), $\Delta\psi$ (blue), $\Delta[K^+]$ (red), and ΔpH (black).

Table S1: Table of model parameters

Parameter Description	Symbol	Value	Units	Reference
Cell Volume	V_{cell}	$4.2 \cdot 10^{-15}$	L	S1
Cell Mass	M_{cell}	$4.62 \cdot 10^{-15}$	kg	calculated from S1
Biomass formed from 1 mol glucose	X_{glu}	156	g	calculated from S2
Biomass yield, ATP	Y_{ATP}^{max}	31	$g \cdot mol^{-1}$	S3
Biomass yield, NH3	Y_{NH3}	121	$g \cdot mol^{-1}$	calculated from S2, S4
Kinetic constant, metabolism (cell volume basis)	$V_{max,1}$	0.0067	$M \cdot s^{-1}$	calculated from S5
Kinetic constant, growth (cell volume basis)	$V_{max,2}$	0.00067	$M \cdot s^{-1}$	calculated from S5, S6
Glucose saturation constant	$K_{m,Glu}$	0.00153	M	estimated from S7
ATP saturation constant	$K_{m,ATP}$	0.01	M	estimated from S8
NH3 saturation constant	$K_{m,NH3}$	0.0001	M	estimated from S9
ADP saturation constant	$K_{m,ADP}$	0.001	M	estimated from S8
Phosphate saturation constant	$K_{m,Pi}$	0.005	M	estimated from S10
ATP maintenance coefficient	$\mu_{maintain}$	0.001	$M \cdot s^{-1}$	estimated from S11
ATP maintenance, saturation constant	$K_{maintain,ATP}$	0.001	M	estimated from S11
Limiting internal pH, metabolism	$pH_{lim,metabolism}$	5	–	S12
Limiting internal pH, growth	$pH_{lim,growth}$	6	–	S13
Forward reaction rate, F-ATPase	k_f	320	s^{-1}	calculated from S14
Backward reaction rate, F-ATPase	k_b	$6.71 \cdot 10^4$	$M^{-1}s^{-1}$	calculated from S14, S15, S16
Step 1 equillibrium constant, F-ATPase	K_1	0.228	–	calculated from S17
Step 2 equillibrium constant, F-ATPase	K_2	117	–	calculated from S17
Step 3 equillibrium constant, F-ATPase	K_3	6.42	–	calculated from S17
Step 5 equillibrium constant, F-ATPase	K_5	221000	–	calculated from S17
Concentration (abundance), F-ATPase	C_{ATPase}	$7.52 \cdot 10^{-8}$	M	estimated from S18
Work distribution, F-ATPase	x	0.66	–	S19
H+/ATP ratio, F-ATPase	n	$3 - 4$	–	S20 and S21

Table S1: Table of model parameters (continued)

Parameter Description	Symbol	Value	Units	Reference
Catalytic rate constant, KcsA	k_d	$3.8 \cdot 10^8$	s^{-1}	calculated from S22
Charge transfer coefficient, KcsA	α	0.5	–	calculated from S22
Equillibrium dissociation constant, KcsA	K_d	0.63	M	S22
Channel opening pKa, KcsA	pKa_{KcsA}	4.2	–	S23
Concentration (abundance), KcsA	C_{KcsA}	$3 \cdot 10^{-8}$	M	estimated from S18; 24
Permeability constant, Lactic acid	P_{Lactic}	3	s^{-1}	calculated from S1; 25
pKa, Lactic Acid	pKa_{Lac}	3.86	–	S1
pKa1, Phosphate	pKa_{p1}	2.15	–	S1
pKa2, Phosphate	pKa_{p2}	7.16	–	S1
pKa3, Phosphate	pKa_{p3}	12.30	–	S1
pKa, Ammonium	pKa_{NH3}	9.30	–	S1

References

S1. Liao, C., Seo, S.-O., Celik, V., Liu, H., Kong, W., Wang, Y., Blaschek, H., Jin, Y.-S. and Lu, T. (2015). Integrated, systems metabolic picture of acetone-butanol-ethanol fermentation by clostridium acetobutylicum. Proceedings of the National Academy of Sciences 112, 8505–8510.

S2. Papoutsakis, E. T. (2000). Equations and calculations for fermentations of butyric acid bacteria. Biotechnology and bioengineering 67, 813–826.

S3. Russell, J. B. and Cook, G. M. (1995). Energetics of bacterial growth: balance of anabolic and catabolic reactions. Microbiological reviews 59, 48–62.

S4. Fagerbakke, K. M., Heldal, M. and Norland, S. (1996). Content of carbon, nitrogen, oxygen, sulfur and phosphorus in native aquatic and cultured bacteria. Aquatic Microbial Ecology 10, 15–27.

S5. Sauer, M., Russmayer, H., Grabherr, R., Peterbauer, C. K. and Marx, H. (2017). The efficient clade: lactic acid bacteria for industrial chemical production. Trends in biotechnology 35, 756–769.

S6. Adamberg, K., Seiman, A. and Vilu, R. (2012). Increased biomass yield of lactococcus lactis

by reduced overconsumption of amino acids and increased catalytic activities of enzymes. PloS one 7, e48223.

S7. Altıok, D., Tokatlı, F. and Harsa, Ş. (2006). Kinetic modelling of lactic acid production from whey by lactobacillus casei (nrrl b-441). Journal of Chemical Technology & Biotechnology: International Research in Process, Environmental & Clean Technology 81, 1190–1197.

S8. Bennett, B. D., Kimball, E. H., Gao, M., Osterhout, R., Van Dien, S. J. and Rabinowitz, J. D. (2009). Absolute metabolite concentrations and implied enzyme active site occupancy in escherichia coli. Nature chemical biology 5, 593–599.

S9. Kim, M., Zhang, Z., Okano, H., Yan, D., Groisman, A. and Hwa, T. (2012). Need-based activation of ammonium uptake in escherichia coli. Molecular systems biology 8, 616.

S10. Mason, P., Carbone, D. P., Cushman, R. and Waggoner, A. (1981). The importance of inorganic phosphate in regulation of energy metabolism of streptococcus lactis.. Journal of Biological Chemistry 256, 1861–1866.

S11. Deng, Y., Beahm, D. R., Ionov, S. and Sarpeshkar, R. (2021). Measuring and modeling energy and power consumption in living microbial cells with a synthetic atp reporter. BMC biology 19, 1–21.

S12. Nannen, N. L. and Hutkins, R. W. (1991). Intracellular ph effects in lactic acid bacteria. Journal of Dairy Science 74, 741–746.

S13. Kashket, E. R. (1987). Bioenergetics of lactic acid bacteria: cytoplasmic ph and osmotolerance. FEMS Microbiology Reviews 3, 233–244.

S14. Gao, Y. Q., Yang, W., Marcus, R. A. and Karplus, M. (2003). A model for the cooperative free energy transduction and kinetics of atp hydrolysis by f1-atpase. Proceedings of the National Academy of Sciences 100, 11339–11344.

S15. Alberty, R. A. (2001). Effect of temperature on standard transformed gibbs energies of formation of reactants at specified ph and ionic strength and apparent equilibrium constants of biochemical reactions. The Journal of Physical Chemistry B 105, 7865–7870.

S16. Milo, R., Jorgensen, P., Moran, U., Weber, G. and Springer, M. (2010). Bionumbers- the database of key numbers in molecular and cell biology. Nucleic acids research 38, D750–D753.

S17. Mukherjee, S. and Warshel, A. (2011). Electrostatic origin of the mechanochemical rotary mechanism and the catalytic dwell of f1-atpase. Proceedings of the National Academy of Sciences 108, 20550–20555.

S18. Ivey, D. M. and Ljungdahl, L. G. (1986). Purification and characterization of the f1-atpase from clostridium thermoaceticum. Journal of bacteriology 165, 252–257.

S19. Adachi, K., Oiwa, K., Nishizaka, T., Furuike, S., Noji, H., Itoh, H., Yoshida, M. and Kinosita Jr, K. (2007). Coupling of rotation and catalysis in f1-atpase revealed by single-molecule imaging and manipulation. Cell 130, 309–321.

S20. Nirody, J. A., Budin, I. and Rangamani, P. (2020). Atp synthase: Evolution, energetics, and membrane interactions. Journal of General Physiology 152.

S21. Soga, N., Kimura, K., Kinosita Jr, K., Yoshida, M. and Suzuki, T. (2017). Perfect chemomechanical coupling of fof1-atp synthase. Proceedings of the National Academy of Sciences 114, 4960–4965.

S22. Nelson, P. H. (2002). A permeation theory for single-file ion channels: Corresponding occupancy states produce michaelis–menten behavior. The Journal of chemical physics 117, 11396–11403.

S23. Chakrapani, S., Cordero-Morales, J. F. and Perozo, E. (2007). A quantitative description of kcsa gating i: macroscopic currents. The Journal of general physiology 130, 465–478.

S24. Zafar, H. and Saier Jr, M. H. (2020). Comparative genomics of the transport proteins of ten lactobacillus strains. Genes 11, 1234.

S25. Walter, A. and Gutknecht, J. (1986). Permeability of small nonelectrolytes through lipid bilayer membranes. The Journal of membrane biology 90, 207–217.
