# Supplementary material for: Modeling control and transduction of electrochemical gradients in acid-stressed bacteria
Source: iScience. 2023 Jun 17;26(7):107140. doi: 10.1016/j.isci.2023.107140 (PMC10316662; doi:10.1016/j.isci.2023.107140)
Supplement: Data S1. Source code used to generate Figures 1–3 [file mmc2.zip › pHtools_release/pHtools/reportsheet.pdf]

pHTools Titration Report Sheet

Experiment #:

Performed by: ____________ Dielectric Constant: ____________

Date: ____________ Sol. Temp. (°C): ____________

pH Probe: ____________ Initial Volume (Liters): ____________

Description of Solution Being Titrated:

____

____

Obs.	Run	Titrant	Molarity of Titrant	Vol. of Titrant Added	pH
1					
2					
3					
4					
5					
6					
7					
8					
9					
10					
11					
12					
13					
14					
15					
16					
17					
18					
19					
20					
21					
22					
23					
24					
25					

Comments:

Measured Quantities (e.g. HPLC)

Compound	Conc.	Compound	Conc
