# Supplementary material for: Modeling control and transduction of electrochemical gradients in acid-stressed bacteria
Source: iScience. 2023 Jun 17;26(7):107140. doi: 10.1016/j.isci.2023.107140 (PMC10316662; doi:10.1016/j.isci.2023.107140)
Supplement: Data S1. Source code used to generate Figures 1–3 [file mmc2.zip › pHtools_release/pHtools_man.pdf]

# pHTools Version 1.3 Software for Modeling pH and Buffer Capacity in Dynamic Systems

Daniel P. Dougherty

Last Modified: 2011/03/23

## Contents

PC Installation . . . . . . . . . . . . . . . . . . . . . . . . . . . . . . . . . 6
UNIX Installation . . . . . . . . . . . . . . . . . . . . . . . . . . . . . . . 6

**Creating Buffer Solutions** **7**

**Generating Titration Curves** **8**
Partial Contributions . . . . . . . . . . . . . . . . . . . . . . . . . . . . . 8

**Generating Buffer Capacity Curves** **8**

**Generating Ionic Strength Curves** **10**

**Generating Sillén Diagrams** **12**

**Capturing the Output** **12**

**Determining the Initial pH of Buffer Solution** **13**

**Reaching a Desired pH** **13**

**Ionic Strength, Temperature and Dielectric Adjustments** **14**

**Finding the Right Buffer** **15**

**Creating Your Own Buffer Database** **15**

**Handling Complex Buffers** **16**

**Managing the Buffer Databases pHTools Searches** **18**

**Combining and Averaging Solutions** **18**

**Buffer Capacity and pH in Dynamic Applications** **19**

**Dealing With Complexation/Precipitation Reactions** **22**

**Brønsted-Lowry Acids and Bases** **23**

**Generalized Titrations** **25**

**Dynamic Systems Modeling** **26**

**Local Polynomial Modeling of Buffers** **28**

**Generalized Additive Modeling of Buffer Mixtures** **29**

**Ionic Strength and Temperature Adjustments** **30**
Ionic Strength of Complex Buffers . . . . . . . . . . . . . . . . . . . . . 31

**Database Organization** **34**
Nickname . . . . . . . . . . . . . . . . . . . . . . . . . . . . . . . 34
Name . . . . . . . . . . . . . . . . . . . . . . . . . . . . . . . . 35
Comment . . . . . . . . . . . . . . . . . . . . . . . . . . . . . . . 35
SaltType . . . . . . . . . . . . . . . . . . . . . . . . . . . . . . . 35
$pK_a$ . . . . . . . . . . . . . . . . . . . . . . . . . . . . . . . . 36
$pK_b$ . . . . . . . . . . . . . . . . . . . . . . . . . . . . . . . . 36
Data . . . . . . . . . . . . . . . . . . . . . . . . . . . . . . . . . 39

**Complex Buffers** **39**

**Using Your Own Smoothing Functions** **39**
Appendix39
CB. . . . . . . . . . . . . . . . . . . . . . . . . . . . . . . . . . . 39
CBGAM. . . . . . . . . . . . . . . . . . . . . . . . . . . . . . . . . 40
CBGAMDER. . . . . . . . . . . . . . . . . . . . . . . . . . . . . . . 41
CBGAMVAL. . . . . . . . . . . . . . . . . . . . . . . . . . . . . . . 41
CONTENTS. . . . . . . . . . . . . . . . . . . . . . . . . . . . . . . 41
BUFFCAPEVAL. . . . . . . . . . . . . . . . . . . . . . . . . . . . . 44
BUFFERSTATS. . . . . . . . . . . . . . . . . . . . . . . . . . . . . 44
CONTAINSDLG. . . . . . . . . . . . . . . . . . . . . . . . . . . . . 45
DCBDH. . . . . . . . . . . . . . . . . . . . . . . . . . . . . . . . . 45
DCBDT. . . . . . . . . . . . . . . . . . . . . . . . . . . . . . . . . 45
DAVIES. . . . . . . . . . . . . . . . . . . . . . . . . . . . . . . . . 46
FINDBUFFER. . . . . . . . . . . . . . . . . . . . . . . . . . . . . . 47

FINDBUFFSET. . . . . . . . . . . . . . . . . . . . . . . . . . . . . . . 47
FINDPI. . . . . . . . . . . . . . . . . . . . . . . . . . . . . . . . . . 48
FINDPI_OBJ. . . . . . . . . . . . . . . . . . . . . . . . . . . . . . . . 49
GETBUFFER. . . . . . . . . . . . . . . . . . . . . . . . . . . . . . . 49
GETSOL. . . . . . . . . . . . . . . . . . . . . . . . . . . . . . . . . 49
INITPH. . . . . . . . . . . . . . . . . . . . . . . . . . . . . . . . . 49
INITPHOBJ. . . . . . . . . . . . . . . . . . . . . . . . . . . . . . . 50
IONICSTR. . . . . . . . . . . . . . . . . . . . . . . . . . . . . . . . 50
KEEP. . . . . . . . . . . . . . . . . . . . . . . . . . . . . . . . . . 51
MANAGEDB. . . . . . . . . . . . . . . . . . . . . . . . . . . . . . . 51
MIXSOL. . . . . . . . . . . . . . . . . . . . . . . . . . . . . . . . . 51
MKADJ. . . . . . . . . . . . . . . . . . . . . . . . . . . . . . . . . 52
MKSOL. . . . . . . . . . . . . . . . . . . . . . . . . . . . . . . . . 52
MULTISELECTDLG. . . . . . . . . . . . . . . . . . . . . . . . . . . 53
OPTIMADJ. . . . . . . . . . . . . . . . . . . . . . . . . . . . . . . 53
PHTOOLSINFO. . . . . . . . . . . . . . . . . . . . . . . . . . . . . 54
PRINTDB. . . . . . . . . . . . . . . . . . . . . . . . . . . . . . . . 54
REPORTSHEET. . . . . . . . . . . . . . . . . . . . . . . . . . . . . 55
RESETALLPOSITIONS. . . . . . . . . . . . . . . . . . . . . . . . . . 55
SEARCHTEXT. . . . . . . . . . . . . . . . . . . . . . . . . . . . . . 55
SETLASTPATH_PHTOOLS. . . . . . . . . . . . . . . . . . . . . . . . 55
SPECIES. . . . . . . . . . . . . . . . . . . . . . . . . . . . . . . . 56
SPECIES_NEW. . . . . . . . . . . . . . . . . . . . . . . . . . . . . . 57
SPREADSTRUCT. . . . . . . . . . . . . . . . . . . . . . . . . . . . . 57
STRUCTCAT. . . . . . . . . . . . . . . . . . . . . . . . . . . . . . . 58
TARGETCONC. . . . . . . . . . . . . . . . . . . . . . . . . . . . . . 59
TARGETCONC_OBJ. . . . . . . . . . . . . . . . . . . . . . . . . . . 60
TARGETPH. . . . . . . . . . . . . . . . . . . . . . . . . . . . . . . 60
TARGETPH_MC. . . . . . . . . . . . . . . . . . . . . . . . . . . . . 61
TARGETPHOBJ. . . . . . . . . . . . . . . . . . . . . . . . . . . . . 62
TARGETSOL. . . . . . . . . . . . . . . . . . . . . . . . . . . . . . . 62
TARGETSOL_CNSTRNTDLG. . . . . . . . . . . . . . . . . . . . . . . 64
TARGETSOL_GUI. . . . . . . . . . . . . . . . . . . . . . . . . . . . 64
TARGETSOL_OBJ. . . . . . . . . . . . . . . . . . . . . . . . . . . . 65
TEST. . . . . . . . . . . . . . . . . . . . . . . . . . . . . . . . . . 65
TESTDATA. . . . . . . . . . . . . . . . . . . . . . . . . . . . . . . 65
UPDATE_OLD_DB. . . . . . . . . . . . . . . . . . . . . . . . . . . . 65
USERDB. . . . . . . . . . . . . . . . . . . . . . . . . . . . . . . . . 65

**Simple Examples** **66**

**Generating Experimental Designs** **68**
Preparation of NaOH Stock . . . . . . . . . . . . . . . . . . . . . . . 70
Calibration of NaOH Stock . . . . . . . . . . . . . . . . . . . . . . . 71
Calibration of HCL Stock . . . . . . . . . . . . . . . . . . . . . . . . 72

# List of Figures

1 Titration plot of a 30mM solution of acetic acid over the default pH range of [2, 12]. . . . . . . . . . . . . . . . . . . . . . . . . . . 9
2 Titration plot of a 30mM solution of acetic acid over pH range of [2, 6]. The pH used were generated evenly over the interval using the Matlab function LINSPACE. . . . . . . . . . . . . . . . . . 9
3 Plot of partial contributions to the titration of a 30mM solution of acetic acid. . . . . . . . . . . . . . . . . . . . . . . . . . . . . 10
4 Plot of the buffer capacity of a 30mM solution of acetic acid. The peak occurs when the pH equals the $pK_a$ of the acid (i.e. 4.75 for acetic acid). . . . . . . . . . . . . . . . . . . . . . . . . . . . . 11
5 Plot of partial contributions to the buffer capacity of a 30mM solution of acetic acid. . . . . . . . . . . . . . . . . . . . . . . . 11
6 Partial ionic strength contributions in a 30mM acetic acid solution. 12
7 Sillén diagram of ionic species of a 30mM acetic acid solution. . . 13
8 Comparison of adjusted and unadjusted buffer capacity curves. Increasing ionic strength tends to cause a decrease in an acids $pK_a$ (up to a point anyway.) . . . . . . . . . . . . . . . . . . . . . 15
9 Screen grab of the FINDBUFFER dialog. You can use the findbuffer dialog to find buffers matching certain criteria. . . . . . . . 16
10 Screen grab of the REPORTSHEET dialog. . . . . . . . . . . . . 17
11 Results of the bioproc example MATLAB code. This is a demonstration of a simplistic model of a homolactic batch fermentation with dynamically varying pH. . . . . . . . . . . . . . . . . . . . . 23
12 Partial contributions to $C_b$ from the components of a 0.01M NaOH solution. In a generalized titration it is *not* the total $C_b$ but rather the partial contribution (solid line) that is relevant. 26
13 Partial contributions to $C_b$ derived from the components of a 0.01M gluconic acid solution. In a generalized titration it is *not* the total $C_b$ but rather the partial contribution (solid line) that is relevant. . . . . . . . . . . . . . . . . . . . . . . . . . . . . . . 27
14 Comparison of the Davies equation with the modification due to Samson et al. (1999). . . . . . . . . . . . . . . . . . . . . . . . . 31
15 Partial buffer capacity diagram of a 15 mM solution of a theoretical acid with 6 $pK_a$. To account for the changes in ionic strength, the $pK_a$'s were modified by their activities as calculated by the Davies equation. The $pK_a$ of the acid at I=0 are 3,5,7,9,11, and 12. 32

16 Partial ionic strength diagram of a 15 mM solution of a theoretical acid with 6 pKa. To account for the changes in ionic strength, the $pK_a$ 's were modified by their activities as calculated by the Davies equation. The $pK_a$ of the acid at I=0 are 3,5,7,9,11, and 12. Clearly, as the pH increases, the predominant species are more negatively charged and have greater affect on the ionic strength. 33
17 A)Reservoir containing sodium hydroxide stock B) $CO_2$ trap. Polyethylene drying tube is first packed with about 3 cm of cotton followed by about 3 cm Drierite$^{TM}$then 3 cm of Ascarite$^{TM}$followed by 3 cm of Drierite and finally more cotton. C) Reservoir outlet to volumetric pipet D) From reservoir outlet connecting to stop-cock 1 of volumetric pipet. E) Double stopcock volumetric pipet. F) Flask containing magnetic stir bar and buffer to be titrated. G) Magnetic stir plate. H) Ring stand . . . . . . . . . . . . . . . . 70

Before You Get Started. . .

There are several conventions used throughout this manual which you should be aware of.

- All Matlab m-files are given formatted in ALL CAPS. This conforms with the Matlab standards for documentation.
- Matlab is case-sensitive so be sure of a function's correct name when using it.

Introduction The pHTools toolbox is a collection of Matlab$^{TM}$files which allow mathematical modeling of buffer systems. The core software routines allow the prediction of buffer capacity,ionic strength, andtitration curves. These routines are then used in other routines to achieve various goals. For example the software allows one to compute the amount of a primary buffer required to achieve a desired pH in secondary buffer. There are also routines that can be incorporated into systems of differential equations for predicting pH , buffer capacity and species concentration in dynamical systems .

In many applications, buffer solutions are complex consisting of many components which may not or can not be measured. pHTools , however, permits one to account for the buffering of complex buffers. The basic approach is to perform Local polynomial regression on the data from experimental titrations of the media. From this data, estimates of buffer capacity and ionic strength are made. A particularly nice feature of pHTools is that if 1 or more components of a complex buffer have been quantified their effects are automatically assimilated into predictions of buffer capacity and ionic strength[1] as well as the other numerical computations mentioned above.

Several preliminary buffer databases come with pHTools which can be added to or modified via graphical interfaces. Graphical interfaces are also available to search the databases for buffers using various selection criteria. Experimental titration data associated with a complex buffer can be entered into a database

[1] See Section

via a graphical interface or one can uses an import/export feature which allows import of text files containing the titration data as well as the export of such data.

Installation

## PC Installation

1. Copy the pHTools software folder onto a drive on your computer
2. Start MATLAB
3. Use MATLAB's PATHTOOL to inform MATLAB of the location of the software.
   (a) Type pathtool at the MATLAB command prompt
   (b) Select the path location of pHTools
   (c) Using the "Add With Subfolders" option add the path
   (d) Click on Save
4. To confirm installation try typing "help pHtools" at the MATLAB command prompt.

## UNIX Installation

On UNIX systems the user will typically not have write access to the directory in which Matlab is installed so the pHTools software con not be saved there. Also, it may be more advantageous for UNIX users on a network to use a startup.m file to set the path location of pHTools rather than PATHTOOL. MATLAB upon start-up will look in the user's home directory for a sub-directory called matlab. If there is a m-file called startup.m within this sub-directory then MATLAB executes this m-file and any commands it contains.

Within the user's home directory create a directory called **matlab**. Create a file called **startup.m** within the **matlab** directory. The startup.m program should be written to add the full filenames of the toolboxes to Matlab's search path. See the example below.

EXAMPLE 1

**Example of a typical startup.m file.**

```
%startup.m
%
disp('Executing user preferences...');

base = pwd; %This gets the present working directory.

%You may need to adjust the base path
%to suit your particular UNIX installation.

addpath(fullfile(base,'pHtools'));
addpath(fullfile(base,'lpregtools));
addpath(fullfile(base,'simplextools'));
```

Tutorial

After you have downloaded and correctly installed the pHtools you should be able to type

```
>> help pHtools
```

at the MATLAB prompt and see a list of available m-files in the pHtools toolbox. You can then type

```
>> help <mfile>
```

for more detailed help on <mfile>.

## Creating Buffer Solutions

The most frequent task you have to perform when using pHTools is creating a solution from several buffers. The function which is used to do this is MKSOL. The input expected by MKSOL is a comma-separated list of buffer names and their molar concentration. For example, to create a 30mM solution of acetic acid, type in at the MATLAB prompt the following code

```
>> S1 = mksol('Acetic',0.03);
```

To create a solution containing multiple components such as lactic acid(30mM), and NaCl(0.5M) you would do

```
>> S2 = mksol('Lactic',0.03,'NaCl',0.5);
```

Some times it is convenient or desirable to create new buffer solutions from previously existing buffer solutions. The standard way to do this is to use MIXSOL, which mixes solutions using given dilution factors. For example, to create a 2:1 S1:S2 solution one would do

```
>> DF = [2 1];
>> DF = DF./sum(DF);
>> S3 = mixsol(S1,DF(1),S2,DF(2));
```

# Generating Titration Curves

Let's see what the predicted titration curve for this solution would look like. Recall that when plotting a titration curve, the moles of base added per liter is plotted on the x-axis and the resultant pH is plotted on y-axis. The function CB returns the moles base added per liter. Try the following at the MATLAB prompt.

```
>> S = mksol('Acetic',0.03);
>> Cb(S);
```

The titration plot automatically created by this command is displayed in Figure 1. *If the output of CB is not assigned to a variable then a figure of the predicted titration is automatically generated.* The default pH range used by pH Tools is [2, 12]. To focus on specific pH you can specify the pH directly in the call to CB. Here we use the standard Matlab function LINSPACE to generate 30 evenly-spaced points in the range [2,6].

```
>> P = linspace(2,6,30);
>> Cb(S,P);
```

The titration plot should now displayed over the pH range [2,6] using 30 equally-space points (see Fig. 2).

## Partial Contributions

A solution may contain several buffers. In order to see how each buffer of the solution contributes to the titration, you can call the CB function with the 'partial' keyword. This will generate output corresponding to the partial buffer component of each buffer in the solution. Note that in the following examples we will specify [ ] as the pH which will cause the default pH range of [2,12] to be used.

```
>> Cb(S,[],'partial');
```

# Generating Buffer Capacity Curves

A titration will give the relationship between the moles of base added per liter of buffer solution and the pH . From a titration curve, the buffer capacitycan be

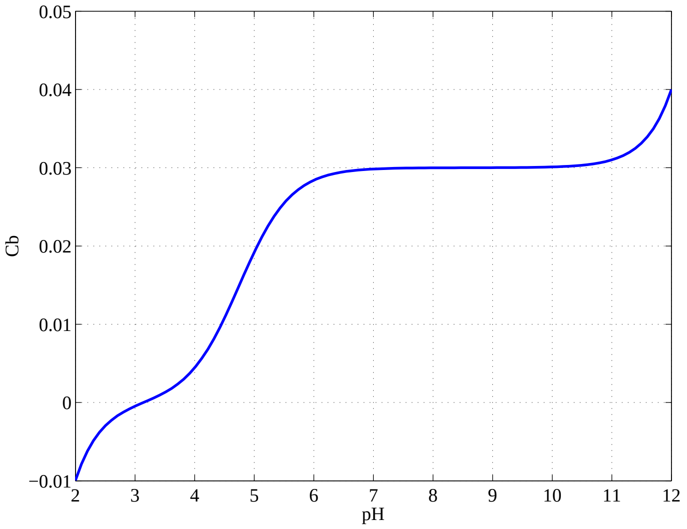


Figure 1: Titration plot of a 30mM solution of acetic acid over the default pH range of [2, 12].

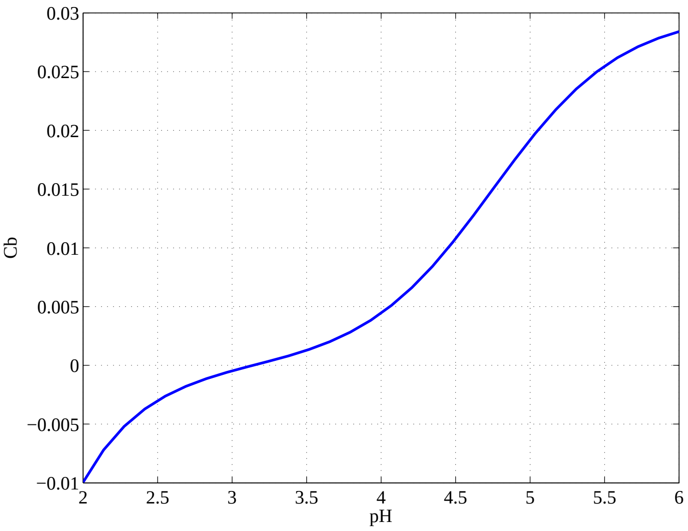


Figure 2: Titration plot of a 30mM solution of acetic acid over pH range of [2, 6]. The pH used were generated evenly over the interval using the Matlab function LINSPACE.

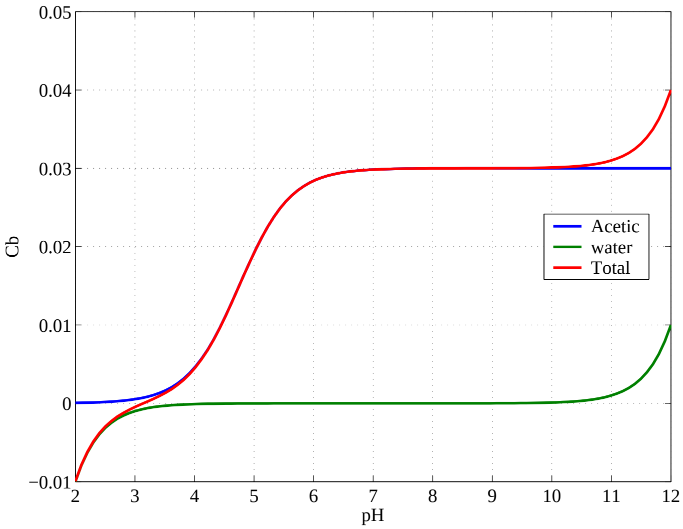


Figure 3: Plot of partial contributions to the titration of a 30mM solution of acetic acid.

determined. The buffer capacity is a measure of the the buffer solution's ability to resist a change in pH with addition of base and is defined mathematically as

$$\beta = \frac{\partial C_b}{\partial \mathrm{pH}} \tag{1}$$

We may explore the buffer capacity of the acetic acid solution by using the function BUFFCAPEVAL (see Fig. 4).

```
>> buffcapeval(S)
```

The syntax of BUFFCAPEVAL is analogous to that of CB. For example, to see the partial contributions to the buffer capacity we can use the following syntax.

```
>> buffcapeval(S,[],'partial');
```

which will produce Fig. 5

# Generating Ionic Strength Curves

An important measure of ion dissociation is the ionic strength of a solution. pH Tools allows you to explore the ionic strength of a buffer solution at any desired pH with the function IONICSTR. The syntax of IONICSTR is analogous to that of CB and BUFFCAPEVAL. For example, to generate a plot of the partial ionic strength contributions of the 30mM acetic acid solution over the default pH range you would enter the following at the MATLAB prompt.

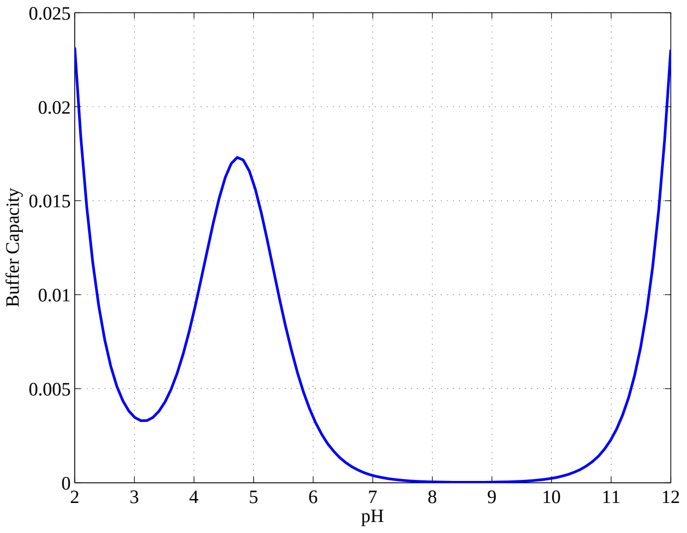


Figure 4: Plot of the buffer capacity of a 30mM solution of acetic acid. The peak occurs when the pH equals the $\mathrm{pK}_a$ of the acid (i.e. 4.75 for acetic acid).

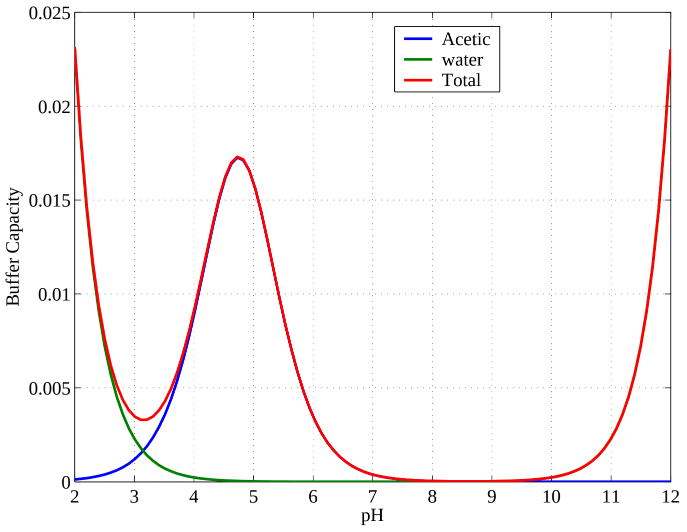


Figure 5: Plot of partial contributions to the buffer capacity of a 30mM solution of acetic acid.

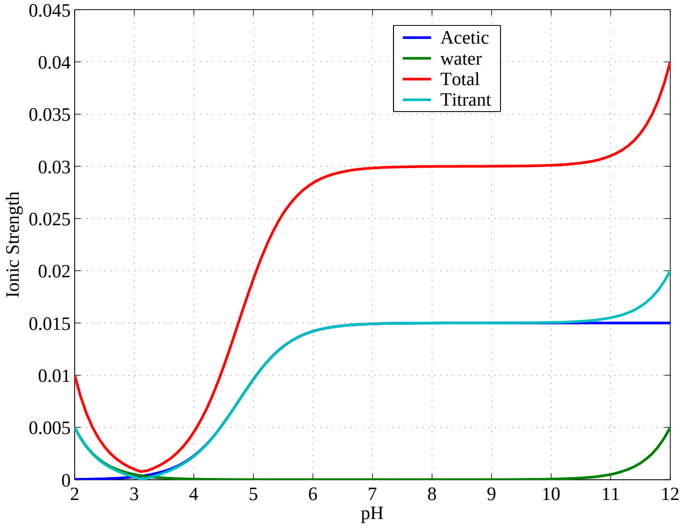


Figure 6: Partial ionic strength contributions in a 30mM acetic acid solution.

```
>> ionicstr(S,[],'partial');
```

which will produce Fig. 6

# Generating Sillén Diagrams

A Sillén diagram is a semi-logarithmic plot of species molar concentrations vs. pH. In a Sillén diagram, the slope of the H+ curve is -1, the slope of the OH- curve is +1 and the points of intersection between 2 species curves correspond with the Ka relating those 2 species. pHTools allows you to produce Sillén diagrams over any desired pH range with the function SPECIES. The syntax of SPECIES is analogous to that of CB and BUFFCAPEVAL except that there is no 'partial' option. For example, to generate a plot of the Sillén diagram of the 30mM acetic acid solution over the default pH range you would enter the following at the MATLAB prompt.

```
>> species(S,[]);
```

which will produce Fig. 7

# Capturing the Output

In the preceding examples, we exploited the default behavior of CB, BUFFCAPEVAL, IONICSTR and SPECIES in generating plots of the desired quantities. In many cases, we will want to use the numerical values generated rather

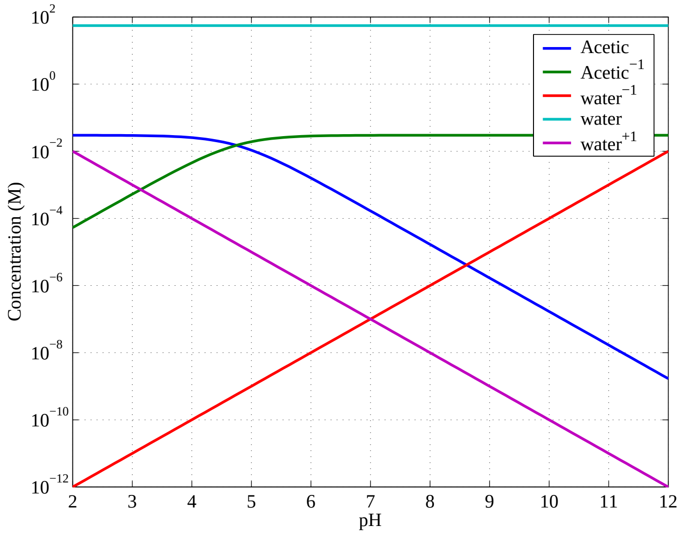


Figure 7: Sillén diagram of ionic species of a 30mM acetic acid solution.

than to see a plot. You can assign the output from any of the just mentioned functions to a variable. For example, to assign the ionic strength prediction to the variable I we would do the following.

```
>> I = ionicstr(S,[],'partial');
```

Now I is a matrix whose columns hold the partial contributions. You should try "capturing the output" of the other functions using the various syntax options.

# Determining the Initial pH of Buffer Solution

The initial pH of a buffer solution can be determined graphically from a plot of the titration curve. The initial pH corresponds to $C_b = 0$. To determine the initial pH algorithmically we must determine the *zero crossing* of the $C_b$ function for a particular solution. pHTools provides the function INITPH for automating this task. For example, to determine the initial pH of the acetic acid solution enter the following code.

```
>> initpH(S)
```

# Reaching a Desired pH

Let's suppose that we really want our 30mM acetic acid solution to have an initial pH of 5.3. We want to add sufficient NaOH to bring about this change.

However, we would like to know ahead of time how much NaOH we will need to add. pHTools provides a standard means for getting this information about any buffer solution you construct. Let's say our stock of NaOH has a concentration of 100mM. Try the following code at the Matlab prompt.

```
>> S2 = mksol('NaOH',0.1);
>> Amnt = targetpH(S,S2,[0 50],5.3)
```

# Ionic Strength, Temperature and Dielectric Adjustments

In all of the function calls mentioned so far, it has been assumed that the ionic strength of the medium is zero, that the temperature is 25°C and the dielectric constant is the same as that for water (78.38). In reality, the ionic strength is never zero. The dielectric constant depends on the material properties of the solvent. The ionic strength, dielectric constant and to a lesser extent the temperature can have an effect on the dissociation of acids and bases. *pHTools allows the user to generate dissociation constant adjustments for any solution over an entire range of pH.* To illustrate, consider the full syntax of the CB function.

$$M = Cb(SOL, pH, 'partial', ADJ); \tag{2}$$

In this syntax ADJ is a data structure containing the dissociation constant adjustments for the components of the buffer solution. To generate adjustments for a particular solution use the function OPTIMADJ as in the following.

```
>> SOL = mksol('Acetic',0.03,'NaCl',0.3422);
>> pH = linspace(2,12,100)';
>> B1 = buffcapeval(SOL,pH);
>> ADJ = optimadj(SOL,pH,25);
>> B2 = buffcapeval(SOL,pH,[],ADJ);
>> plot(pH,B1,'r--');
>> hold on;
>> plot(pH,B2,'r-');
>> legend('Unadjusted','Adjusted');
```

Analgous syntaxes hold for the function BUFFCAPEVAL, IONICSTR and-SPECIES. For the functions INITPH and TARGETPH the syntax is slightly different. For example,

```
>> initpH(SOL,[0,14],'adjust',35)
```

determines the zero-rossing of the Cb function while "adjusting" for the effects of ionic strength and temperature. In this case, the temperature is 35°C. A similar syntax is used for TARGETPH.

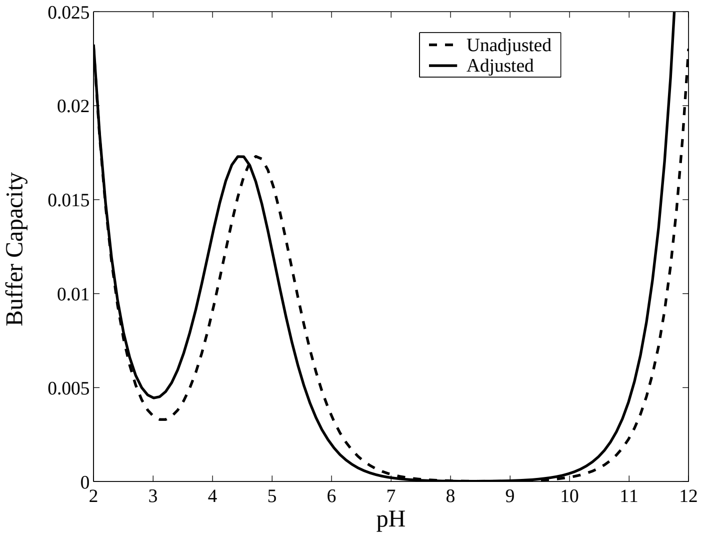


Figure 8: Comparison of adjusted and unadjusted buffer capacity curves. Increasing ionic strength tends to cause a decrease in an acids $pK_a$ (up to a point anyway.)

# Finding the Right Buffer

There are buffers available within the databases which come with the pHTools installation. You may also have created your own buffers and buffer databases. To search for buffers matching certain criteria you can use the FINDBUFFER dialog.

Steps to finding a buffer.

- Open the FINDBUFFER dialog.
- Select one or more databases to search.
- Type in criteria for searching.
- Click on search.

# Creating Your Own Buffer Database

While there are several buffers available within the databases which come with pHTools you will no doubt want to add your own buffers. In this section we will describe how this is done.

Steps to creating a new buffer.

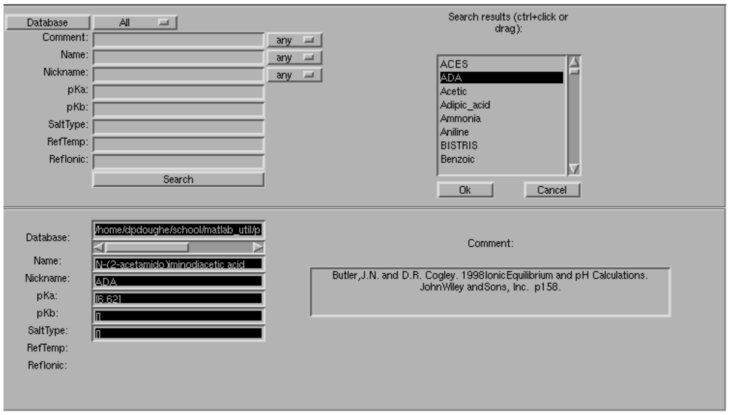


Figure 9: Screen grab of the FINDBUFFER dialog. You can use the findbuffer dialog to find buffers matching certain criteria.

- Open the REPORTSHEET dialog.
- Select or type in the file path of a database (.mat) file. The .mat file need not already exist.
- Type in the Nickname of the buffer you want to add to the database. This will be used as the MATLAB variable name associated with the buffer.
- Enter any information or comments.
- Click on Save.
- Add more buffers to the database or exit the REPORTSHEET dialog.

# Handling Complex Buffers

Complex buffers are specified by experimental titration data. A special dialog is provided by pHTools to facilitate the creating of complex buffers. The REPORTSHEET dialog allows you to enter titration data and obtain a titration plot in real-time. You can also import and export data to and from tab-delimited text files. The format of such text files must adhere to the following standard.

- A single header line is assumed. You may put anything here that you want.
- First column (numeric) contains the observation numbers. These determine the order of the sampling and should never repeat.
- Second column (numeric) contains the run number.

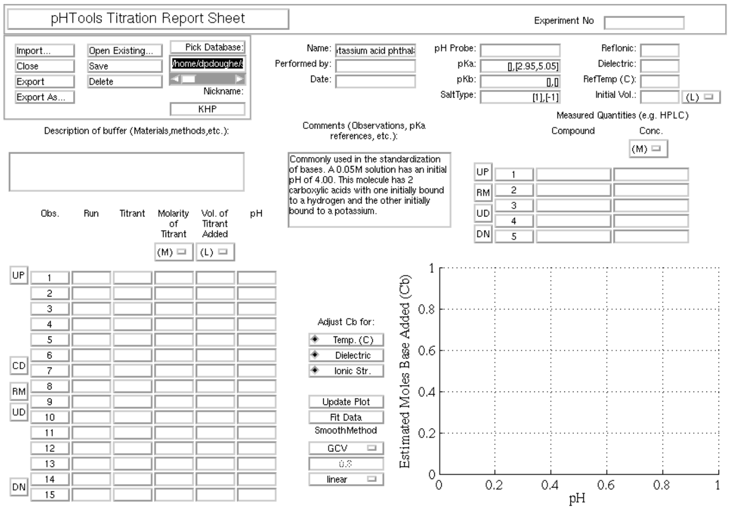


Figure 10: Screen grab of the REPORTSHEET dialog.

- Third column (character) contains the nickname of the titrant.
- Fourth column (numeric) contains the molarity of the titrant.
- Fifth column (numeric) contains the current volume of the solution.
- Sixth column (numeric) contains the volume of titrant just added.
- Seventh column (numeric) contains the current pH of the solution.

The following example illustrates an acceptable tab-delimited text file ready for import.

```
Obs     Run     Titrant Molarity        Vol     Vol_Added       pH
1       1       NaOH    3       0.2     0       3.86
2       1       NaOH    3       0.201   0.001   4.1
3       1       NaOH    3       0.202   0.001   4.3
4       1       NaOH    3       0.203   0.001   4.5
5       1       NaOH    3       0.204   0.001   4.9
6       2       HCl     3       0.2     0       3.84
7       2       HCl     3       0.201   0.001   3.8
8       2       HCl     3       0.202   0.001   3.6
9       2       HCl     3       0.203   0.001   3.4
```

Experimentalists often may have quantified one or more of the chemicals within a solution that may be contributing to the buffer capacity of a complex

buffer. pHTools can take advantage of this information and provide better estimates of the buffer capacity and ionic strength of complex buffers. In order for pHTools to use such information you simply need to specify the measurements in the Contains field of the complex buffer. This can be done easily through the Contains dialog of the REPORTSHEET dialog. **You must enter the Nickname of the buffer that you have measured and its molar concentration.** If the measured component is not currently a buffer in any database then just give it a reasonable Nickname. If at some future time you can enter such a compound as simple or complex buffer then pHTools will be able to take advantage of it. *It is advisable to enter contained compounds even when they are not thought to contribute to the buffer capacity.* These might include compounds such as sugars and polymers. This information may be useful in dynamic simulations or in other inquires involving the complex buffer.

It is possible to nest complex buffers. That is a complex buffer can contain other complex buffers. In such cases, the concentration of the contained buffer is interpreted as a relative concentration.

## Managing the Buffer Databases pHTools Searches

The mechanism that pHTools uses to reference buffers stored within the databases is meant to facilitate speed during dynamic simulations and to simplify function calls within programs. Searching databases for any conceivable set of criteria is nice but computationally wasteful. As you work more with pHTools you will find that you may be using the same buffers over and over. pHTools provides a mechanism for searching only certain buffer databases which can greatly reduce search time and speed up numerical computations. You should strive to create small yet meaningful devisions among your databases (.mat files) to facilitate searching and sharing your databases with other pHTools users.

- While there may be many databases on MATLAB's search path, only those paths specified in the text file `mypHtoolsdb.txt` are recognized by pHTools
- You can use USERDB to add or remove database paths from the file `mypHtoolsdb.txt`.
- You need only refer to a buffer by its Nickname. pHTools will search all databases listed in `mypHtoolsdb.txt` for the **first** matching buffer.

## Combining and Averaging Solutions

New solutions can be created from existing solutions be mixing or averaging them. Practically, this amounts to assigning a weighting factor to each solution. During the combination process each solution's concentrations are multiplied by the weighting factor. An important detail of the mixing process is that the

solvent, water in most cases, does not change concentration in the same way as the rest of the solution components. The function MIXSOL automates the process of forming new solutions from pre-existing ones.

The ability to modify and combine pre-existing solutions using MIXSOL is especially beneficial in dynamical settings where computation expense is at a premium. In particular, MIXSOL does not need to search databases to find the buffer components.

EXAMPLE 2

Make a 50/25 (percent by volume) mixture of solution A and solution B. Solution A is a 0.05 M solution of acetic acid and solution B is a 0.01 M solution of malic acid.

---

In a laboratory, this could be done by taking 500 mL of solution A, mixing it with 250 mL of solution B and bringing the total volume up to 1000 mL.

```
>>    A = mksol('Acetic',0.05);
>>    B = mksol('Malic',0.01);
>>    C = mixsol(A,0.5,B,0.25);
```

## Buffer Capacity and pH in Dynamic Applications

In this section, we discuss how pHTools can be used to dynamically update pH predictions. While incapable of producing lag phase or stationary phase, we consider for simplicity the following model for homolactic fermentation of glucose. The density of the bacteria responsible for the fermentation will be represented by $N$, the molar concentration of glucose is represented by $G$, and the molar concentration of *total* lactic acid by $L$.

$$\frac{dN}{dt} = \mu\text{max}\left(\frac{G}{K_G + G}\right) N \tag{3}$$

$$\frac{dG}{dt} = -\mathcal{Y}GN \tag{4}$$

$$\frac{dL}{dt} = \mathcal{Y}GN \tag{5}$$

$$\frac{d\text{pH}}{dt} = \frac{1}{\beta_t}\frac{dC_b}{dt} \tag{6}$$

The parameters have physiological meaning. The parameter $\mu$ is the maximum specific growth rate of the bacteria, $\mathcal{Y}$ is the specific fermentation rate of the bacteria, and $K_G$ is the glucose concentration at which growth rate is 1/2 saturated.

Here is a driver program *drivebioproc.m* which calls the ode solver and plots the results of the simulation. Notice that a parameter struct is used so that

familar names of parameters can be used within the code. Depending on the values of parameters used, a stiff ODE solver, in this case ode15s, may be more efficient due to the rapid relative changes in variable magnitudes. Of key importance is efficiency in database look-ups. In this code we use a *persistent* variable to store the growth medium solution (SOL). The SOL variable will be empty the first time the model is called by the main program. In that case the database will be queried to form up the buffer. However, in subsequent calls only updates to the component concentrations are needed. Any searching of the database is avoided and the resulting code runs approximately 10X faster than if the database were queried repeatedly.

```
%DRIVEBIOPROC Dynamic growth and pH change in buffered media.
%This is script m-file which drives the bioproc.m code and
%then plots some simulation results.
%This is an example showcasing the kind of MATLAB code
%that can be used to make efficient and readable models.
%The example is not meant to be biologically realistic
%but rather a simple toy example.
%

close all;
clear all;

PARAMS = struct('mu',1.2,...       %Max. growth rate of bacteria
                'Y',1.e-7,...      %Specific fermentation rate
                'Kg',0.0001,...    %Glucose at which growth rate is 1/2 saturated
                'N_init',1.e4,...  %Initial bacterial density (CFU/mL)
                'G_init',0.03,...  %Initial glucose concentration (M)
                'L_init',0.00,...  %Initial lactic acid concentration (M)
                'pH_init',6.8);    %Initial pH

OPTS = odeset;
model2use = 'bioproc';

varnames = feval(model2use,[],[],'variables');
Nvar = length(varnames);
yinit = zeros(1,Nvar);

initconds = strcat(varnames,'_init');

for i=1:Nvar
        yinit(i) = PARAMS.(initconds{i});
end

tspan = [0 10];
```

```
%More efficient to use a
%stiff-solver due to time-scales.
[T,Y] = ode15s('bioproc',tspan,yinit,OPTS,PARAMS);

%Store the model predictions in a struct by variable name.
PRED = cell2struct(mat2cell(Y,size(Y,1),ones(1,size(Y,2))),varnames,2);

subplot(3,1,1);
plot(T,[PRED.G,PRED.L]);
legend('Glucose (M)','Lactic (M)');
subplot(3,1,2);
semilogy(T,PRED.N);
legend('Bacteria (CFU/mL)');
subplot(3,1,3);
plot(T,PRED.pH);
legend('pH');
xlabel('Time (h)');


AX = findobj(get(gcf,'Children'),'Type','axes','Tag','');
set(AX,'FontSize',24,'Box','off');
L = findobj(gcf,'Type','line');
set(L,'LineWidth',3);
G = findobj(gcf,'Tag','legend');
set(G,'FontSize',14);
set(gcf,'PaperUnits','inches','PaperSize',[6.45,5]);
print -dpng bioproc
```

Now, here is the Matlab ODE *bioproc.m* which calculates the rates. Figure 11 illustrates the results of this dynamic buffered fermentation simulation.

```
function varargout = bioproc(t,y,flag,P)
%BIOPROC Dynamic growth and pH change in buffered media.
%This is an example showcasing the kind of MATLAB code
%that can be used to make efficient and readable models.
%The example is not meant to be biologically realistic
%but rather a simple toy example.
%

%Using a persistent variable allows us to only query the
%database just once.  This greatly increases efficiency.
persistent SOL
```

```
switch flag
case ''
        N  = y(1);%Bacteria
        G  = y(2);%Glucose
        L  = y(3);%Lactic acid
        pH = y(4);%pH

        if isempty(SOL)
                %Only need query database first time through.
                SOL = mksol('Lactic',0);
        else
                SOL(1).Conc = L;
        end

        Buff = buffcapeval(SOL,y(4));

        dNdt = (P.('mu')*G/(P.('Kg')+G))*N;
        dGdt = -P.('Y')*G*N;
        dLdt =  P.('Y')*G*N;;

        DCbDt = -dCbdt(SOL,pH,[],[],'Lactic',dLdt);
        dpHdt = (1/Buff)*DCbDt;

        varargout{1} = [dNdt,dGdt,dLdt,dpHdt]';

case 'variables'
        varargout{1} = {'N','G','L','pH'};

otherwise
        error(['Unexpected flag ',flag,' to ',mfilename]);
end
```

# Dealing With Complexation/Precipitation Reactions

As was noted earlier, one of the assumptions required by pHTools is that complexation and precipitation reactions can be ignored. In some applications, it may be the case that these reactions significantly effect.

Computational Approach

In this section, we present the basic formula useful in modeling pH and buffer capacity.

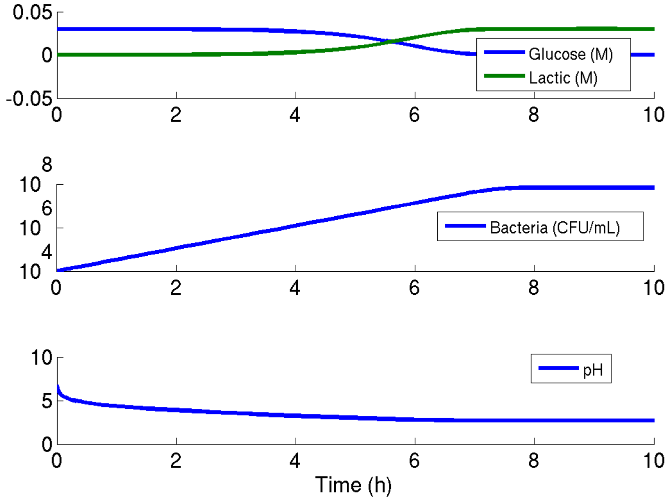


Figure 11: Results of the bioproc example MATLAB code. This is a demonstration of a simplistic model of a homolactic batch fermentation with dynamically varying pH.

# Brønsted-Lowry Acids and Bases

Van Slyke (1922) introduced buffer capacity as a measure of the ability of a buffer to resist change in pH with the addition of base during a titration. He defined the buffer capacity ($\beta$) of a solution as

$$\beta \equiv \frac{\partial C_b}{\partial \mathrm{pH}} \tag{7}$$

where $C_b$ is the number of moles of base added per liter of solution. Van Slyke (1922) was also the first to demonstrate the additivity of buffer capacities. For example if a solution consists of $n$ compounds each of which contribute to $\beta$, then a decomposition of the form

$$\beta = \beta_1 + \beta_2 + \ldots + \beta_n \tag{8}$$

is admissible where $\beta_i$ is called the $i$th *partial buffer capacity* component.

When the dissociation constants for various compounds are known, the partial buffer contribution of that compound can be expressed explicitly. For example, the buffer capacity of an acid with a single dissociable proton has the form

$$\beta = \ln(10)\left(C\frac{K_a\left[\mathrm{H}^+\right]}{(\left[\mathrm{H}^+\right]+K_a)^2}\right) + \ln(10)\left(\frac{K_w}{\left[\mathrm{H}^+\right]} + \left[\mathrm{H}^+\right]\right) \tag{9}$$

where C is the concentration of the acid, $K_a$ is the dissociation constant of the single proton and $K_W$ is the ion-product constant of water ($K_w \approx 1.8 \times 10^{-16}$ at 25°C and zero ionic strength). Here $\ln(10)(K_w/\left[H^+\right] + \left[H^+\right])$ is the partial buffer contribution of water and $\ln(10)C(K_a\left[H^+\right])/(\left[H^+\right]+K_a)^2)$ is the partial contribution due to the acid. For an acid with 2 dissociable protons the expression for $\beta$ becomes

$$\beta = \ln(10)C\left(\frac{K_{a1}\left[H^+\right]^3 + 4K_{a1}K_{a2}\left[H^+\right]^2 + K_{a1}^2K_{a2}\left[H^+\right]}{\left(\left[H^+\right]^2 + K_{a1}\left[H^+\right] + K_{a1}K_{a2}\right)^2}\right) \quad (10)$$

$$+\ln(10)\left(\frac{K_w}{\left[H^+\right]} + \left[H^+\right]\right) \quad (11)$$

where $K_{a1}$ and $K_{a2}$ are the acid dissociation constants for the primary and secondary dissociable protons respectively. Notice that the partial contribution of a diprotic acid is <u>not</u> equivalent to the sum of 2 monoprotic acids (Butler and Cogley, 1998, p.134).

Similar formula may be derived for buffers with any number of dissociable groups. Eqn. 12 (proven in the Appendix) is a computational formula useful in the computation of the partial buffer capacity of a compound with an arbitrary number ($n$) dissociable groups.

$$C_b = \ln(10)\left[H^+\right]\frac{C\sum_{i=1}^{n}\left[(n+1-i)\left[H^+\right]^{i-1}\prod_{j=1}^{n+1-i}K_{aj}\right]}{\sum_{i=1}^{n+1}\left[\left[H^+\right]^{i-1}\prod_{j=1}^{n+1-i}K_{aj}\right]} \quad (12)$$

Along with the fact that $d\left[H^+\right]/d\text{pH} = -\ln(10)\left[H^+\right]$, Eqn 12 can be differentiated with respect to $\left[H^+\right]$ to give a formula for the buffer capacity of an $n$-protic acid.

$$\beta = \ln(10)\left[H^+\right]\left[\frac{BC - AD}{B^2}\right] \quad (13)$$

where

$$A = C\sum_{i=1}^{n}\left[(n+1-i)\left[H^+\right]^{i-1}\prod_{j=1}^{n+1-i}K_{aj}\right] \quad (14)$$

$$B = \sum_{i=1}^{n+1}\left[\left[H^+\right]^{i-1}\prod_{j=1}^{n+1-i}K_{aj}\right] \quad (15)$$

$$C = C\sum_{i=2}^{n}\left[(n+1-i)(i-1)\left[H^+\right]^{i-2}\prod_{j=1}^{n+1-i}(K_{aj})\right] \quad (16)$$

$$D = \sum_{i=2}^{n+1} \left[ (i-1) \left[ \mathrm{H}^{+} \right]^{i-2} \prod_{j=1}^{n+1-i} (K_{aj}) \right] \tag{17}$$

Note that Eqn. 13 reduces to Eqn. 9 for $n = 1$ and reduces to Eqn. 10 for $n = 2$.

Van Slyke (1922) points out that the formula corresponding to a base of concentration $C$ is obtained by simply replacing the $K_a$ in the expressions with $Kw/K_b$. Also, for a compound having both acid and base components (amphoteric) the expression for $\beta$ is simply the summation of its acid and base components. If the titration is by acid then Eqn. 12 can still be used assuming the convention that $C_b$ take on negative values with magnitude equal to the number of moles of *acid* added per liter of solution.

# Generalized Titrations

Normally, the titrants used in performing titrations are strong acids or strong bases. Because these titrants dissociate essentially completely, their contribution to $C_b$ is easily calculated. In many instances (e.g. food grade applications) strong acids or bases are undesirable and weak acids or bases are used instead. In order to still use the data from such titrations in calculating buffer capacities, a more general approach to calculating $C_b$ must be taken. When a weak acid or weak base is used as the titrant we will refer to the process of inferring a relationship between pH and $C_b$ as a *Generalized Titration*.

The contribution to $C_b$ from a weak acid or base will vary depending on the extent to which it dissociates and thereby depend on the pH. For clarity we let $C_b$ (pH) indicate $C_b$ at a particular pH. If we denote the partial contribution *of the weak titrant* to $C_b$ (pH) by $C_\tau$(pH) then

$$C_b\ (\mathrm{pH}) = -C_\tau(\mathrm{pH}) \tag{18}$$

That is, the moles of base added is the negative of the moles of base that would be required to bring the buffer $\tau$ to the pH in question. Similarly, for complex buffers containing more than 1 buffering compound $\tau_1$,$\tau_2$,..., we can use the additivity of the partial $C_b$ and make the following definition

$$C_b\ (\mathrm{pH}) = -(C_{\tau_1}(\mathrm{pH}) + C_{\tau_2}(\mathrm{pH}) + \ldots + C_{\tau n}(\mathrm{pH})) \tag{19}$$

That is, the moles of base added is equal to the negative of the sum of the contributions from the weak acids or bases. To illustrate these points consider Fig. 12 which is a plot of the partial $C_b$ of a 0.01M NaOH (p$K_b \approx$ -3) solution over the pH range 2–12. Over this interval the contribution to $C_b$ remains a constant 0.01 M because NaOH is a strong base and its dissociation is essentially complete over this pH range. In contrast consider Fig. 13 which gives the partial contributions to $C_b$ from a 0.01M solution of gluconic acid (p$K_a \approx$ 3.6). At low

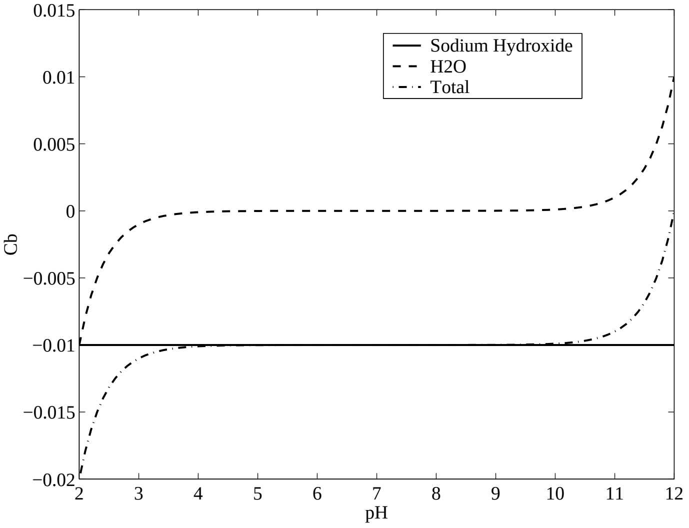


Figure 12: Partial contributions to $C_b$ from the components of a 0.01M NaOH solution. In a generalized titration it is *not* the total $C_b$ but rather the partial contribution (solid line) that is relevant.

pH, this weak acid is completely protonated and therefore makes no contribution to $C_b$ . Conversely at high pH all of the gluconic acid is dissociated and its contribution to $C_b$ is equivalent to the negative of its concentration (i.e. the conjugate base of a weak acid is a strong base). *Clearly to determine the moles of base available for titration we need to consider the dissociation of the weak titrant at the particular pH in question.* Thus, for generalized titrations, determination of a mathematical relationship between $C_b$ and pH requires a function which maps pH to $C_b$ (an approach which at first may seem counter-intuitive to those acquainted only with strong acid or strong base titrants). **It is the convention of this software to treat pH as the predictor variable and $C_b$ as the response variable.**

# Dynamic Systems Modeling

The buffer capacity measure is also useful for dynamic system modeling. It can provide a simple mechanism for updating pH predictions over time. To develop an updating formula, simply re-write $\beta$ as

$$\frac{\partial \mathrm{pH}}{\partial t} = \frac{1}{\beta}\frac{\partial C_b}{\partial t} \tag{20}$$

We may assume that $\left[\mathrm{H}^+\right]$ is constant during the small time interval $\partial t$ so that the partial contribution to the change in pH due to a small change in the

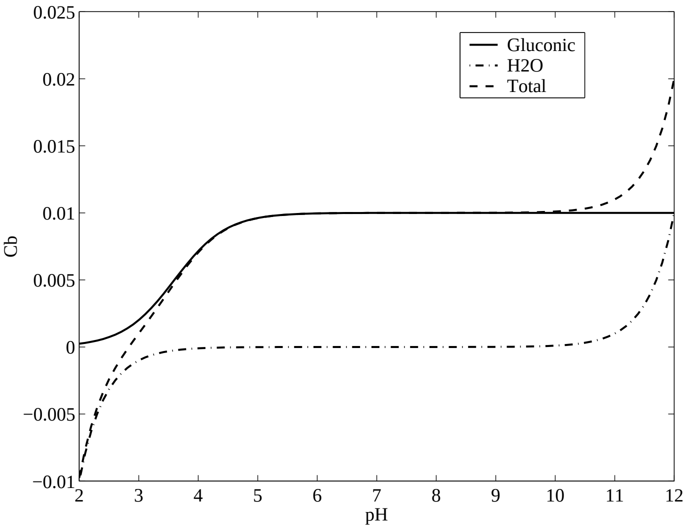


Figure 13: Partial contributions to $C_b$ derived from the components of a 0.01M gluconic acid solution. In a generalized titration it is *not* the total $C_b$ but rather the partial contribution (solid line) that is relevant.

concentration of an $n$-protic acid is

$$\frac{\partial \text{pH}}{\partial t} = \frac{1}{\beta_t} \left( \frac{\frac{dC}{dt} \sum_{i=2}^{n+1} \left( \prod_{j=1}^{i-1} K_{a_j} \left[ \text{H}^+ \right]^{n+1-i} \right)}{\sum_{i=1}^{n+1} \left( \prod_{j=1}^{i-1} K_{a_j} \left[ \text{H}^+ \right]^{n+1-i} \right)} \right) \tag{21}$$

$\beta_t$ is the buffer capacity given the concentration of the acid at time $t$ and $dC/dt$ is the instantaneous rate of change in the concentration of the acid. $\beta_t$ indicates that the buffer capacity is calculated given the concentrations of weak acids and bases at time $t$. Consider this simplistic Monod type model for single substrate ($G$) limited batch fermentation. The bacteria ($N$) produce a weak acid ($L$) as a metabolic waste product.

$$\frac{dN}{dt} = \mu\text{max} \left( \frac{G}{K_G + G} \right) N \tag{22}$$

$$\frac{dG}{dt} = -\frac{\mu\text{max}}{\mathcal{Y}} \left( \frac{G}{K_G + G} \right) N \tag{23}$$

$$\frac{dL}{dt} = \frac{\mu\text{max}}{\mathcal{Y}} \left( \frac{G}{K_G + G} \right) N \tag{24}$$

$$\frac{d\text{pH}}{dt} = \frac{1}{\beta_t} \frac{dC_b}{dt} \tag{25}$$

While this model gives the basic mathematical formalism there are obstacles which need to be over come to make this approach viable. Microbial growth

media is often quite complex consisting of undefined components such as yeast extract, peptic digests etc. In order to predict pH continuously in time we need a way of calculating $\beta_t$. The next section will discuss how this is to be done.

# Local Polynomial Modeling of Buffers

Eqns. 13and 12 are applicable so long as the concentrations of all acids and bases and their dissociation equilibria are known. Unfortunately, these requirements are usually not met in biological applications due to the complexity and undefined nature of most growth media and biological buffers. Although the buffer capacities of such solutions can be quite complex chemically, it is reasonable to assume that the relationship between $C_b$ and pH should be smooth and continuous. While a titration can be used to determine the relationship between $C_b$ and pH there is error in measurement. We also can not observe the effect of adding a small amount of base at every possible pH value. We therefore require a flexible regression procedure which will provide a continuous differentiable prediction function for $C_b$ given pH .

In order to obtain such a predictor, we use local polynomial regression (for an introduction to the subject see (Fan and Gijbels, 1996, pp.57-107)) to predict pH as a function of $C_b$ . There are several reasons for choosing local polynomial regression over other smoothing methods. Local polynomial regression automatically controls for model complexity, automatically adjusts for unevenly spaced design points and behaves well at the boundaries of the data. The ease with which derivative estimates are obtained is especially nice since it allows for the straightforward calculation of $\beta$. Predictors of $C_b$ and $\beta$ for mixtures of undefined and defined buffers consist of summations of local polynomial regression estimators for the complex components and algebraic predictors of defined buffer components as described above. Thus, the local polynomial regression procedure provides a transparent means of extending the computational formula given for buffers with known dissociation constants to complex buffers containing undefined components.

One important consideration relating to estimating derivatives from experimental data is the influence of spurious data points. For example, most pH probes manifest drift in measurement during use and must be re-calibrated. Re-calibrations should also be performed whenever the pH exceeds the calibration pH . The occurrences of drift, re-calibration or incorrectly recorded data can lead to small jumps in the titration data that can create spurious jumps in the estimation of $\beta$. For this reason, iteratively re-weighted least squares is used to down-weight unlikely data points.

As pointed out in (Cleveland et al., 1988) the critical assumptions of local polynomial regression are

1. $\epsilon_i \sim i.i.d.N(0, \sigma^2)$

2. $\sigma^2$ is constant

3. $E(\hat{\mathcal{F}}(\text{pH}\,)) = \mathcal{F}(\text{pH}\,)(\hat{\mathcal{F}}$ is an unbiased estimator)

A simple way to verify these assumptions is to view a plot of the residuals versus the predicted or to view a normal probability plot of the residuals.

# Generalized Additive Modeling of Buffer Mixtures

In many instances buffers solutions are mixtures of defined as well as undefined components. In this section we discuss some formal statistical approaches for modeling mixtures and methods for inference.

Consider the titration of weak acid at concentration $C$ in a complex buffer.

$$C_{b\ i} \quad = \quad \frac{CKa_1}{\left[\text{H}^+\right]_i + Ka_1} + \mathcal{F}(\text{pH}\,_i) + \epsilon_i \tag{26}$$

where $\epsilon_i \sim N(0, \sigma^2)$.

The methods of inference for local polynomial regression is well known (Cleveland et al., 1988)

Let $\mathcal{H}$ represent the pseudo-projection matrix which maps the observations to predictions (the Hat matrix)

$$\hat{y} \quad = \quad \mathcal{H}y \tag{27}$$

In the case of local polynomial regression the Hat matrix is of the form

$$\mathcal{H} \quad = \quad X(X'WX)^{-1}X'W \tag{28}$$

Letting $R_{\mathcal{H}} = (I - \mathcal{H})$ we have that

$$\epsilon \quad = \quad R_{\mathcal{H}}y \tag{29}$$

Thus the variance covariance matrix for the vector $\hat{y}$ is $\sigma^2\mathcal{H}\mathcal{H}'$ and the variance covariance matrix for $\epsilon$ is $\sigma^2 R_{\mathcal{H}} R'_{\mathcal{H}}$. Using the fact that the trace of a pseudo-projection matrix is equivalent to its rank, we can define the following degrees of freedom we can formulate an F test for the difference between the fit of a null model and and alternative model.

$$\nu_1 \quad = \quad tr(R_{\mathcal{H}2} - R_{\mathcal{H}1}) \tag{30}$$
$$\nu_2 \quad = \quad tr((R_{\mathcal{H}2} - R_{\mathcal{H}1})(R_{\mathcal{H}2} - R_{\mathcal{H}1})' \tag{31}$$
$$\delta_1 \quad = \quad tr(R_{\mathcal{H}1}) \tag{32}$$
$$\delta_2 \quad = \quad tr(R_{\mathcal{H}3} R'_{\mathcal{H}2}) \tag{33}$$

Then to test model 2 against model 1 we formulate the score

$$\hat{F} = \frac{\frac{RSS_2 - RSS_1}{df2 - df1}}{\frac{RSS_1}{df1}} \tag{34}$$

$$= \frac{(y' R_{\mathcal{H}2} y - y' R_{\mathcal{H}1} y)/\nu_1}{(y' R_{\mathcal{H}1} y)/\delta_1} \tag{35}$$

which is distributed approximately as F with numerator degrees of freedom $\nu_1^2/\nu_2$ and $\delta_1^2/\delta_2$.

This methodology allows us to test for the significance of each components contribution to the buffering of the solution. This is carried out by re-estimation of $\mathcal{F}$ after the particular defined component in question is removed from the model. The new fit is then compared to the old and significance of the F-test indicates significance of the buffering component. In addition one can test for the contribution to buffering of the undefined components.

# Ionic Strength and Temperature Adjustments

Ionic strength is a measure of the electrical interactions occurring within a solution. For relatively dilute solutions ionic strength is adequately defined by

$$I = \sum_{i=1}^{n} (c_i z_i^2) \tag{36}$$

where $c_i$ is the concentration of the $i^{th}$ ion in the solution and $z_i$ is its charge.

Debye and Hückle derived thermodynamic equations governing the properties of non-ideal solutions of electrolytes. However, the Debye-Huckle equations are well known to behave poorly for ionic strengths above 0.1M (Butler and Cogley, 1998).

A common method for modifying activity coefficients and to improve the accuracy of ion-dissociation models is the Davies equation. The original Davies equation predicts the activity coefficient of the $i^{th}$ ion by

$$-\log(\gamma_i) = A z_i^2 \left( \frac{\sqrt{I}}{1 + \sqrt{I}} - bI \right) \tag{37}$$

where $A = 1.825 \times 10^6 (\epsilon T)^{-3/2}$. $\epsilon$ is the dielectric constant of the solvent ($\epsilon = 78.3808$ for water) and T is the temperature in Kelvin.

The standard Davies equation can predict ion activities for ionic strengths up to 0.5 M. At higher ionic strengths, an increased tendency for ion-ion pairing reduces the number of ions contributing to the ionic strength. In such regimes, the Davies equation over-estimates the Higher ionic strengths can be modeled by reducing the salting out parameter $b$. However this reduces accuracy at low ionic strength. Samson et al. (1999), however, give a simple modification of

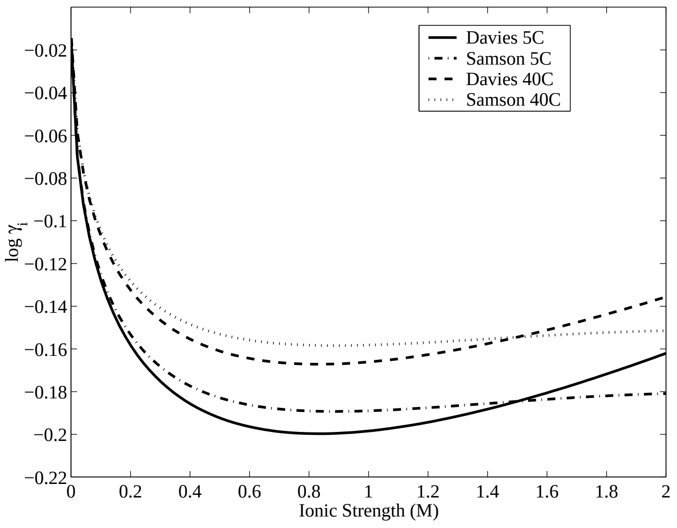


Figure 14: Comparison of the Davies equation with the modification due to Samson et al. (1999).

the standard Davies equation that reduces $b$ linearly in proportion to the ionic strength.

$$-\log(\gamma_i) \quad = \quad Az_i^2\left(\frac{\sqrt{I}}{1+\sqrt{I}} - ((-1/30)I + b)I\right) \tag{38}$$

Samson et al. suggest that this modified version yields reliable results up to an ionic strength of 1.2 M.

## Ionic Strength of Complex Buffers

In this section we will introduce a useful lower bound for the ionic strength conribution from a complex buffer. Consider, the charge balance of a solution of NaOH and C moles $L^-$ monoprotic weak acid HA.

$$\left[Na^+\right] + \left[\mathrm{H}^+\right] \quad = \quad \left[OH^-\right] + \left[A^-\right] \tag{39}$$

which is re-arranged to give $C_b$ .

$$\left[Na^+\right] \quad = \quad \left[OH^-\right] + \left[A^-\right] - \left[\mathrm{H}^+\right] \tag{40}$$

$$C_b \quad = \quad \frac{CK_a}{K_a + \left[\mathrm{H}^+\right]} + \frac{K_\mathrm{W}}{\left[OH^-\right]} - \left[\mathrm{H}^+\right] \tag{41}$$

If we compare this to the ionic strength of the solution

$$I \quad = \quad 0.5(\left[Na^+\right] + \left[OH^-\right] + \left[A^-\right] + \left[H^+\right]) \tag{42}$$

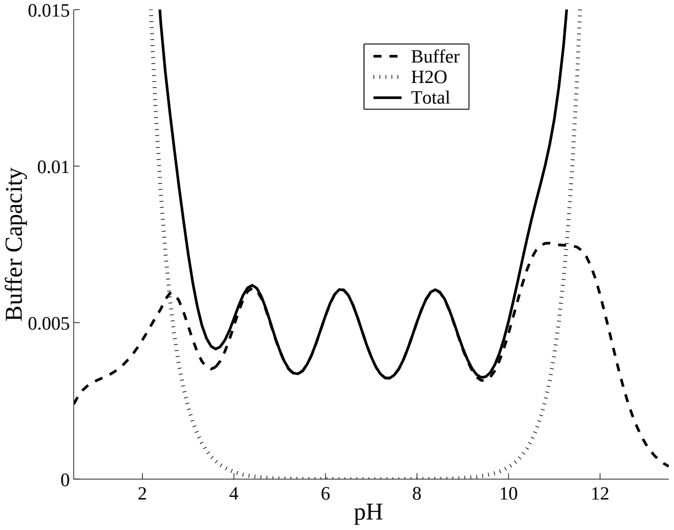


Figure 15: Partial buffer capacity diagram of a 15 mM solution of a theoretical acid with 6 p$K_a$. To account for the changes in ionic strength, the p$K_a$'s were modified by their activities as calculated by the Davies equation. The p$K_a$ of the acid at I=0 are 3,5,7,9,11, and 12.

we notice that $I = C_b + \left[\mathrm{H}^+\right]$. Unfortunately this relationship does not hold when compounds with multiple dissociable groups or salts containing ions with charges greater than 1 or -1 are present. However, in such cases it will always be the case that $I \geq C_b + \left[\mathrm{H}^+\right]$. **This fact leads us to suggest $C_b + \left[\mathbf{H}^+\right]$ as a lower bound for the ionic strength in complex buffers.** We view this as a convenient improvement over the zero ionic strength assumption.

The Nuts n' Bolts of pHTools Software Now we will discuss some of the nitty-gritty details of the pHTools software. Recall that S was the variable storing the information of a 0.03M acetic acid solution. We can see the data structure of S by typing S and the Matlab prompt without any semi-colon.

```
>> S

S =

2x1 struct array with fields:
    Comment
    Contains
    Data
    Func
    Name
    RefIonic
```

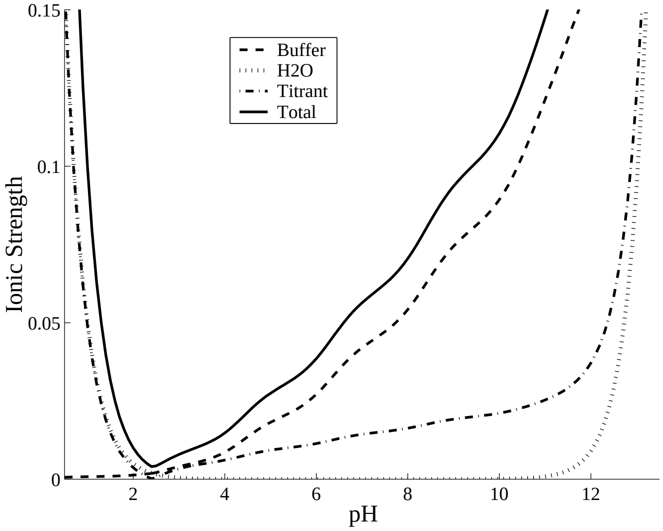


Figure 16: Partial ionic strength diagram of a 15 mM solution of a theoretical acid with 6 pKa. To account for the changes in ionic strength, the $pK_a$ 's were modified by their activities as calculated by the Davies equation. The $pK_a$ of the acid at I=0 are 3,5,7,9,11, and 12. Clearly, as the pH increases, the predominant species are more negatively charged and have greater affect on the ionic strength.

```
    RefTemp
    SaltType
    pKa
    pKb
    Nickname
    Conc
```

This says that S is an array with 2 rows and 1 column. Each row in the array contains a struct. To reference the first element in the array try

```
>> S(1)

ans =

     Comment: 'Merck'
    Contains: []
        Data: []
        Func: []
        Name: 'Acetic'
    RefIonic: 0
     RefTemp: 25
    SaltType: {[]}
         pKa: {[4.7500]}
         pKb: {[]}
    Nickname: 'Acetic'
        Conc: 0.0300
```

Here we see all the information about the first buffer in the solution including its molar concentration which is 0.03 molar as was desired. We notice also that acetic acid has a p$K_a$ of 4.75 at 0 ionic strength and 25°C. The comment Merck refers to the fact that the information was obtained from the Merck manual.

# Database Organization

This section describes the structure of the buffer databases that pHTools uses. The standard data file format for Matlab is the mat-file (.mat). mat-files are binary files which can be quickly read directly into working memory by Matlab. Each buffer database is housed within a mat-file. *The buffers are the variables which get stored in the mat-file.* There may be many buffers stored within a single mat-file.

### Nickname

The buffers stored in the database are Matlab variables and therefore must be given valid Matlab variable names. *The variable associated with a buffer is*

*called its Nickname.* In other words, the Nickname of a buffer is the valid Matlab variable name that is used to refer to the buffer stored within a mat-file. *Names containing spaces, arithmetic operators or names in which the first character is a number are disallowed.* Underscores in the nickname are legal however. For example 1,2,3 triaminopropane is not a legal Nickname but triaminopropane is valid.

### Name

In most cases the chemical name of a compound can not be used as the Nickname. Therefore, a field in the database is reserved to hold a string representing the true chemical name of a buffer. Any characters are valid in the Name field. For example 1,2,3 triaminopropane is a legal Name.

### Comment

The comment field is a block of arbitrary text associated with a buffer. Typically this text will cite the source of any dissociation constants or, if the buffer is complex, any experimental conditions encountered during a titration.

### SaltType

A salt is technically defined as the neutral product of the reaction between and acid and a base. In the context of pHTools all salts are considered to be completely dissociated. Each dissociated ion is associated with a charge. *The SaltType field specifies the charge on each dissociable ion of a buffer molecule.* A dissociable ion is designated by [] The order of the charges is not important, however the same order should be used among the pKa, pKb and SaltType fields. *A single empty field for SaltType indicates that the buffer is not a salt but rather a simple Brønsted-Lowry acid or base (e.g. acetic acid and lactic acid).*

> pHTools assumes that dissociation constants are relative to a neutral form of the compound (this may be a conjugate acid or base associated with 1 or more ions, a salt or a simple Brønsted-Lowry acid or base not associated with any ions). The reasoning behind this convention is that the charged forms exist only in solution – it is the neutral forms that are actually weighed out in preparing buffers.
>
> Please note that many sources do not follow this convention but rather list only the $pK_a$ of particular compounds. This can create great confusion unless the chemical structure of the compound is known and the appropriate $pK_a$ can be converted to $pK_b$ ($K_a = K_W / K_b$).

Table 1: Buffer fields for $NaCl$

| | |
|---|---|
| Comment: | ′Table salt.′ |
| Contains: | |
| Data: | |
| Func: | |
| Name: | ′Sodium chloride′ |
| RefIonic: | 0 |
| RefTemp: | 25 |
| SaltType: | {[-1], [1]} |
| pKa: | {[ ], [ ]} |
| pKb: | {[ ], [ ]} |
| Nickname: | ’NaCl’ |

Table 2: Buffer fields for $Ca(Cl)_2$

| | |
|---|---|
| Comment: | |
| Contains: | |
| Data: | |
| Func: | |
| Name: | Calcium chloride |
| RefIonic: | 0 |
| RefTemp: | 25 |
| SaltType: | {[2], [-1], [-1]} |
| pKa: | {[ ], [ ],[ ]} |
| pKb: | {[ ], [ ],[ ]} |
| Nickname: | ’CaCl2’ |

### pK$_a$

Each ion can have multiple Brønsted-Lowry dissociations. For example, the malate ion has 2 dissociation sites with different dissociation constants at each site. See the examples given below for the various possibilities.

### pK$_b$

The conventions for listing pK$_b$ are similar to those for pK$_a$ . See the examples given below for the various possibilities.

Note that potassium acid phthalate has both a pK$_a$ and a pK$_b$ . This means that KHP is a zwitterion.

Table 3: Buffer fields for $Ca(OH)_2$

| | |
|---|---|
| Comment: | |
| Contains: | |
| Data: | |
| Func: | |
| Name: | Calcium hydroxide |
| RefIonic: | 0 |
| RefTemp: | 25 |
| SaltType: | {[2], [-1], [-1]} |
| pKa: | {[ ], [ ]} |
| pKb: | {[ ], [1.5], [1.5]} |
| Nickname: | 'CaOH2' |

Table 4: Buffer fields for $Ca(lactate)_2$

| | |
|---|---|
| Comment: | |
| Contains: | |
| Data: | |
| Func: | |
| Name: | Sodium chloride |
| RefIonic: | 0 |
| RefTemp: | 25 |
| SaltType: | {[2],[-1],[-1]} |
| pKa: | {[ ],[ ],[ ]} |
| pKb: | {[ ],[10.14], [10.14]} |
| Nickname: | 'CaLactate' |

Table 5: Buffer fields for Lactic Acid

| | |
|---|---|
| Comment: | Table salt. |
| Contains: | |
| Data: | |
| Func: | |
| Name: | Sodium chloride |
| RefIonic: | 0 |
| RefTemp: | 25 |
| SaltType: | {[ ]} |
| pKa: | {[3.86]} |
| pKb: | {[ ]} |
| Nickname: | 'Lactic' |

Table 6: Buffer fields for Malic Acid

| | |
|---|---|
| Comment: | Table salt. |
| Contains: | |
| Data: | |
| Func: | |
| Name: | Sodium chloride |
| RefIonic: | 0 |
| RefTemp: | 25 |
| SaltType: | {[ ]} |
| pKa: | {[3.4, 5.05]} |
| pKb: | {[ ]} |
| Nickname: | 'Malic' |

Table 7: Buffer fields for Potassium Acid Phthalate

| | |
|---|---|
| Comment: | [Commonly used in standardization of bases. A 0.05M solution has an initial pH of 4.00.] |
| Contains: | [] |
| Data: | [] |
| Func: | [] |
| Name: | 'Potassium acid phthalate' |
| RefIonic: | [] |
| RefTemp: | [] |
| SaltType: | {[]} |
| pKa: | {[5.0500]} |
| pKb: | {[11.0500]} |
| Nickname: | 'KHP' |

**Data**

The Data field holds the titration data for complex buffers . A smoothed version of the $C_b$ and pH values given by the data can be stored in the Func field (see the section of the Func field).

## Complex Buffers

Complex buffers contain components which we either do not or can not quantify. In order to relate Cb to pH we perform titrations and smooth the resulting titration curve. In pHtools complex buffers are stored as structs with the following fields

**DerivFun** Name of a function FUN such that feval(FUN,Smooth,Cb) returns a prediction of the pH at the given Cb

**EvalFun** Name of a function FUN such that feval(Fun,Smooth,Cb) returns a prediction of the derivative of pH with respect to Cb at the given Cb

**Smooth** A dataobject of some sort that when called with the DerivFun or EvalFun behaves in the intended manner. This is usually also a struct.

## Using Your Own Smoothing Functions

The user configuration file identifies smoothing methods available to pHtools. To have pHtools use a smoothing method of your own you need to add the name of the m-file which contains the smoothing method. For a smoothing m-file to be compatible with pHtools the following requirements must be met.

1. The Function must accept as input the Run, pH, Cb,Temperature, and Contains information (see CBGAM for an example).
2. The function must produce a complex buffer struct (i.e. a struct with the fields DerivFun, EvalFun and Smooth).

Notice that a valid smoothing function can be a GUI itself. This allows for example use of buttons to tune adjustable parameters of use of other toolboxes such as MATLAB's Curvefitting Toolbox.
Function Reference

## CB.

```
Cb Return the theoretical moles base added for a solution
   at specified pH and temperature.
   M = CB(SOL,pH) returns Cb at the specified pH.
      If [] is input for the pH, then the default pH range
```

```
   [2 12] is used.
M = CB(SOL,pH,'partial') returns partial contributions
   from each buffer solution component. If [] is input
   instead, the partials are not produced (the default).
M = CB(SOL,pH,'partial',ADJ) additionally use the
   adjustment coefficients in ADJ. If ADJ is [] then
   I=0, T=25, and E=78.38 are assumed (the default).

When called with no output argument, this function plots its
result.

Example:
      %Generate a titration curve for a 12mM solution.
      %of malic acid.
      sol = mksol('Malic',0.012);
      mypH = [2:0.1:12]';
      myCb = Cb(sol,mypH);
      figure(1);
      plot(myCb,mypH,'ro-');
      xlabel('Cb'); ylabel('pH');

See also BUFFCAPEVAL, IONICSTR, DCBDH, DCBDT, OPTIMADJ.
```

## CBGAM.

```
CbGAM Return a buffer struct resulting from a GAM fit.
   B = CBGAM(Run,ph,cb,Contains) returns a buffer struct
   resulting from fitting a generalized additive model to
   the input data.  RUN corresponds to a titration run,
   pH is the measured pH, cb is the predicted moles of
   base added per liter of solution and Contains is a
   struct containing the contained buffers (complex
   buffers only).

   Note: CBGAMDER, CBGAMVAL, CBGAMDER and CBGAM are smoothing routines
   intended for use only with the LPREGTOOLS toolbox. If you
   want to use your own smoothing routines then read the
   pHTools manual.
```

## CBGAMDER.

```
CBGAMDER Evaluate the derivative of GAM-type buffer model.
   C = CBGAMDER(FUNC,pH,T,Contains) returns the predicted dCbdpH
   at the specifed pH and temperature.  FUNC is a structure
   produced by LPREG, pH is the specified pH, T is the temperature
   and Contains is a Contains struct belonging to a buffer.

   See also CB, CBGAMVAL, CBGAMDER, LPREG.

   Note: CBGAMDER, CBGAMVAL, CBGAMDER and CBGAM are smoothing routines
   intended for use only with the LPREGTOOLS toolbox. If you
   want to use your own smoothing routines then read the
   pHTools manual.
```

## CBGAMVAL.

```
CbGAMval Evaluate a generalized additive buffer model.
   C = CbGAMval(func,pH,T,Contains) returns the predicted moles
   of base added per liter of solution at the given pH
   and temperature.  FUNC is a structure produced by LPREG, pH
   is the specified pH, T is the temperature and Contains is a
   Contains struct belonging to a buffer.

   See also CB, CBGAMVAL, CBGAMDER, LPREG.

   Note: CBGAMDER, CBGAMVAL, CBGAMDER and CBGAM are smoothing routines
   intended for use only with the LPREGTOOLS toolbox. If you
   want to use your own smoothing routines then read the
   pHTools manual.
```

## CONTENTS.

```
 General purpose commands.
 MATLAB Version 7.1 (R14SP3) 26-Jul-2005

 General information.
   syntax        - Help on MATLAB command syntax.
   demo          - Run demonstrations.
```

```
  ver          - MATLAB, Simulink and toolbox version information.
  version      - MATLAB version information.

Managing the workspace.
  who          - List current variables.
  whos         - List current variables, long form.
  clear        - Clear variables and functions from memory.
  pack         - Consolidate workspace memory.
  load         - Load workspace variables from disk.
  save         - Save workspace variables to disk.
  saveas       - Save Figure or model to desired output format.
  memory       - Help for memory limitations.
  recycle      - Set option to move deleted files to recycle folder.
  quit         - Quit MATLAB session.
  exit         - Exit from MATLAB.

Managing commands and functions.
  what         - List MATLAB-specific files in directory.
  type         - List M-file.
  open         - Open files by extension.
  which        - Locate functions and files.
  pcode        - Create pre-parsed pseudo-code file (P-file).
  mex          - Compile MEX-function.
  inmem        - List functions in memory.
  namelengthmax - Maximum length of MATLAB function or variable name.

Managing the search path.
  path         - Get/set search path.
  addpath      - Add directory to search path.
  rmpath       - Remove directory from search path.
  rehash       - Refresh function and file system caches.
  import       - Import Java packages into the current scope.
  finfo        - Identify file type against standard file handlers on path.
  genpath      - Generate recursive toolbox path.
  savepath     - Save the current MATLAB path in the pathdef.m file.

Managing the java search path.
  javaaddpath   - Add directories to the dynamic java path.
  javaclasspath - Get and set java path.
  javarmpath    - Remove directory from dynamic java path.

Controlling the command window.
  echo         - Echo commands in M-files.
  more         - Control paged output in command window.
  diary        - Save text of MATLAB session.
  format       - Set output format.
```

```
  beep          - Produce beep sound.
  desktop       - Start and query the MATLAB Desktop.
  preferences   - Bring up MATLAB user settable preferences dialog.

Operating system commands.
  cd            - Change current working directory.
  copyfile      - Copy file or directory.
  movefile      - Move file or directory.
  delete        - Delete file or graphics object.
  pwd           - Show (print) current working directory.
  dir           - List directory.
  ls            - List directory.
  fileattrib    - Set or get attributes of files and directories.
  isdir         - True if argument is a directory.
  mkdir         - Make new directory.
  rmdir         - Remove directory.
  getenv        - Get environment variable.
  !             - Execute operating system command (see PUNCT).
  dos           - Execute DOS command and return result.
  unix          - Execute UNIX command and return result.
  system        - Execute system command and return result.
  perl          - Execute Perl command and return the result.
  computer      - Computer type.
  isunix        - True for the UNIX version of MATLAB.
  ispc          - True for the PC (Windows) version of MATLAB.

Debugging.
  debug         - List debugging commands.
  mexdebug      - Debug MEX-files.

Tools to locate dependent functions of an M-file.
  depfun        - Locate dependent functions of an M-file or P-file.
  depdir        - Locate dependent directories of an M-file or P-file.

Loading and calling shared libraries.
  calllib          - Call a function in an external library.
  libpointer       - Creates a pointer object for use with external libraries.
  libstruct        - Creates a structure pointer for use with external libraries.
  libisloaded      - True if the specified shared library is loaded.
  loadlibrary      - Load a shared library into MATLAB.
  libfunctions     - Return information on functions in an external library.
  libfunctionsview - View the functions in an external library.
  unloadlibrary    - Unload a shared library loaded with LOADLIBRARY.
  java             - Using Java from within MATLAB.
  usejava          - True if the specified Java feature is supported in MATLAB.
```

```
 See also LANG, DATATYPES, IOFUN, GRAPHICS, OPS, STRFUN, TIMEFUN,
 MATFUN, DEMOS, GRAPHICS, DATAFUN, UITOOLS, DOC, PUNCT, ARITH.

   Overloaded functions or methods (ones with the same name in other directories)
      help timer/Contents.m
      help serial/Contents.m
      help instrument/Contents.m
      help icinterface/Contents.m
      help audioplayer/Contents.m
      help audiorecorder/Contents.m
```

---

## BUFFCAPEVAL.

---

```
BUFFCAPEVAL Calculate buffer capacity of a solution at specific pH.
   B = BUFFCAPEVAL(SOL,pH) calculates the buffer capacity of a
   buffer solution at specified pH.
   B = BUFFCAPEVAL(SOL,pH,'partial') returns a matrix whose
   columns are the partial buffering capacities.  Note: The last
   column is always reserved for the partial buffer capacity due
   to water.
   B = BUFFCAPEVAL(SOL,pH,'partial',ADJ) additionally use the ADJivity
   coefficients in ADJ. If ADJ is [] then I=0, T=25, and E=78.38-8
   is assumed (the default).

   When called with no output argument, this function plots its
   result.

   Example:
      %Determine the buffer capacity of
      %10mM malic acid and 25 mM lADJic acid.
      sol = mksol('Malic',0.01,'LADJic',0.025);
      subplot(2,1,1);
      buffcapeval(sol,[],'partial');
      subplot(2,1,2);
      buffcapeval(sol,[]);

   See also CB, IONICSTR, DCBDH, DCBDT, OPTIMADJ.
```

---

## BUFFERSTATS.

---

```
BUFFERSTATS Obtain lack of fit statistics for buffer components.
```

## CONTAINSDLG.

```
CONTAINSDLG Graphical interface for specifying
   the buffers contained within another.

   This is a utility function used by EDITBUFFER.
```

## DCBDH.

```
DCBDH Return the derivative of moles base with respect to
proton concentration (i.e. dCb/dH).
   M = DCBDH(SOL,pH) returns dCb/dH at the specified pH.
      If [] is input for the pH, then the default pH range
      [2 12] is used.
   M = DCBDH(SOL,pH,'partial') returns partial contributions
      from each buffer solution component. If [] is input
      instead, the partials are not produced (the default).
   M = DCBDH(SOL,pH,'partial',ADJ)additionally use the adjustment
      coefficients in ADJ. If ADJ is [] then I=0 T=25 is
      assumed (the default).

   Example:
      sol = mksol('Malic',0.012);
      pH = linspace(3,11,50);
      dcbdh = dCbdH(sol,pH);
```

## DCBDT.

```
DCBDT Return the change in Cb with time at specified pH
   and temperature.

   M = DCBDT(SOL,[],[],[],'B1',dB1dt,'B2',dB2dt,...) returns
   dCb/dt at the default pH range [2 12]. 'B1' and 'B2' are
   the partial buffer components and dB1dt and dB2dt are their
   respective time derivatives.
```

```
M = DCBDT(SOL,pH,'partial',[],'B1',dB1dt,'B2',dB2dt,...)
returns partial contributions from each buffer solution
component. If [] is input instead, the partials are not
produced (the default).

M = DCBDT(SOL,pH,'partial',ADJ,'B1',dB1dt,'B2',dB2dt,...)
additionally use the ADJivity coefficients in ADJ. If ADJ
is [] then I=0 T=25 is assumed (the default).

When called with no output argument, this function plots its
result.

Note: The derivatives of any unspecified buffers are
assumed to be 0.
```

---

## DAVIES.

---

```
DAVIES Return adjustment for dissociation constants given ion
   charges, ionic strength, temperature and dielectric
   constant of the solvent.

   DAVIES(Z,I,T,E) returns the negative log (base 10)
      of the adjustment for each Ka. Each column of Z
      holds the charges of the charged ions in
      a particular equilibrium.  Each column of Z
      corresponds to a particular Ka.

      I should be a column vector where each row
      of I can hold a different ionic strength.

      T holds the temperature in
      degrees C E L S I U S.  The number of
      rows in I and T must be the same.
      In this way, activities are
      calculated under (potentially) different
      environmental conditions.

      E is the dielectric constant of the solvent.

   If I is empty then an ionic strength of 0 is assumed.
   If T is empty then a temperature of of 25 C is assumed.
   If E is empty then a dielectric constant of 78.3808 is
```

```
used.
```

## FINDBUFFER.

```
FINDBUFFER Search the database for buffers with specific characteristics.
   B = FINDBUFFER(...) returns a structure containing the buffers.
   [B,INFO] = FINDBUFFER(...) also returns the database locations.

   Searches can specify the following:

   'Database' : Limit search to a few databases (default is all).
 'BufferType' : Search for type of buffer (all, simple, complex)
       'Name' : The name (or part thereof) of the buffer.
   'Nickname' : The name of the variable storing the buffer.
        'pKa' : Value or range of pKa.
        'pKb' : Value or range of pKb.
       'func' : Currently not available.
    'Comment' : Match words or phrases in Comment section.
    'RefTemp' : Value or range of reference temperatures.
   'RefIonic' : Value or range of reference ionic strengths.
   'SaltType' : Charges on ions of a salt.
    'Options' : See FINDBUFFSET.

   Example:
      B = findbuffer('pKa',[3.86]); %Finds buffers with pKa equal to 3.86
      B = findbuffer('pKa',[0 4]); %Finds buffers with pKa between 0 and 4.
      %Find buffer with the word universal in the comment section.
      B = findbuffer('Comment','universal');
      opts = findbuffset('CommentSearch','phrase');

   See also FINDBUFFSET
```

## FINDBUFFSET.

```
FINDBUFFSET Options structure for FINDBUFFER
   FINDBUFFSET controls the behavior of FINDBUFFER.

   Example:
      findbuffset %Display the possibilities.
      findbuffset('CommentSearch','phrase');
```

```
    findbuffset('NameSearch','all');
    findbuffset('NicknameSearch','any','NameSearch','all');

 See also FINDBUFFER
```

---

## FINDPI.

---

```
FINDPI Estimate the isoelectric point for a species in a solution.
 PI = FINDPI(SOL) attempts to determine PI values for every
 ion in the solution SOL except salts.
 [PI,TYPE] = FINDPI(SOL) returns the type of molecule predominant
 at the PI.  Type is either 'uncharged' or 'zwitterion'.
 [PI,TYPE] = FINDPI(SOL,COMPOUND) only returns the results for
 COMPOUND.
 [PI,TYPE] = FINDPI(SOL,COMPOUND,R) searches the PI using the
 initial guess in R.  If R is a 2 element vector, the search is
 performed over the range (R(1),R(2)) and the initial guess is taken
 to be MEAN(R).
 [PI,TYPE] = FINDPI(SOL,COMPOUND,R,'adjust') determines the PI
 adjusting activity coefficients.
 [PI,TYPE] = FINDPI(SOL,COMPOUND,R,'adjust',T,E) also adjusts for
 temperature (T) and dielectric constant E.

 Example:
    Find isoelectric point of a weak acid.  Such a compound
    has no iso-electric point!  But numerically we should
    always hit a lower bound of pH.

   S = mksol('Acetic',0.01);
   [Pi,Type] = findpi(S,'Acetic',[2 12])
   %Try lowering search bound
   [Pi,Type] = findpi(S,'Acetic',[0 4])

  %Here is a case where there is actually a Pi.
  S = mksol('glycine',0.01);
  [Pi,Type] = findpi(S,'glycine')

  %Verify the estimate of Pi for glycine is zero to laboratory
  %precision...
  Species = species(S,Pi.glycine);
  ISO = Species.glycine.p1 - Species.glycine.m1
```

---

## FINDPI_OBJ.

---

---

## GETBUFFER.

---

```
GETBUFFER Get 1 or more buffers from the databases based on their Nicknames.
        [A,INFO] = GETBUFFER(N) returns a cell array of buffers in A
       and a structure containing the database location of each
       Nickname. N is a cell array of Nicknames in the buffer databases.
       The result is sorted by Nickname.

   Example:
      Buffers = {'Lactic','Malic','ACES'};
      [A,I] = getbuffer(Buffers);
```

---

## GETSOL.

---

```
GETSOL Extract information about the buffers in a solution.
I = GETSOL(S,flag) where S is a solution as created by MKSOL and
flag is one of 'Nickname','pKa','pKb','Type','Comment',or 'Conc'.
```

---

## INITPH.

---

```
INITPH Estimate the initial pH of a solution.
   INITPH(SOL) returns an estimate of the initial pH of a solution.
   The algorithm finds the pH at which Cb equals zero.  The default
   search range for pH is [2,12].  By default the ionic strength is
   assumed to be equal to 0 and T=25 celsius.

   INITPH(SOL,R) finds the initial pH searching over the range R.
   R should be a 2 element vector specifying a pH range.

   INITPH(SOL,R,'adjust') searches for the initial pH over the interval R
   using ionic strength adjustments.
```

```
INITPH(SOL,R,'adjust',T) searches for the initial pH over the interval R
using ionic strength and tempeature adjustments where T is the
temperature in celsius.

Example:
   S = mksol('Lactic',0.01);
   pHi = initpH(S)

   S = mksol('Aniline',0.01);
   pHi = initpH(S)
   pHi = initpH(S,[0 14])

See also BUFFCAPEVAL, TARGETPH
```

---

## INITPHOBJ.

---

```
INITPHOBJ Objective function for INITPH.
   The initial pH is estimated as
   the pH at which Cb equals zero.
```

---

## IONICSTR.

---

```
IONICSTR Return the ionic strength of a solution
   at a particular pH.  IONICSTR assumes that
   all pH adjustments are accomplished by strong
   acid or strong base with 1 -1 charges (e.g.
   NaOH and HCl).

   M = IONICSTR(SOL,pH) ionic strength at a particular pH.

   M = IONICSTR(SOL,pH,'partial') return partial contributions
   to the ionic strength.
   M = IONICSTR(SOL,pH,...,ADJ) ionic strength using the activity
   coefficents in ADJ. If ADJ is empty then *no* adjustments
   are made.
   M = IONICSTR(SOL,pH,...,...,N) multiplies the contribution
   from the titrant by N.  This allows one to consider titrants
   with multiple dissociable groups.  By default N = 1 which
   assumes a 1-1 titrants such as NaOH or HCl. For example,
   if calcium hydroxide is the titrant, then N=2.
```

```
   When called with no output argument, this function plots its
   result.

   See also BUFFCAPEVAL, CB, DCBDH, DCBDT, OPTIMADJ.
```

---

## KEEP.

---

```
KEEP keeps the caller workspace variables of your choice and clear the rest.
       Its usage is just like "clear" but only for variables.

       Xiaoning (David) Yang   xyang@lanl.gov 1998
       Revision based on comments from Michael McPartland,
       michael@gaitalf.mgh.harvard.edu, 1999
```

---

## MANAGEDB.

---

```
MANAGEDB Manage buffer databases.
   MANAGEDB allows one to view and edit descriptions of databases
   as well as create and delete entire databases.
```

---

## MIXSOL.

---

```
MIXSOL Combine 1 or more solutions by specifying dilution factors.
   MIXSOL(SOL1,W1,SOL2,W2,SOL3,W3,...);
   Inputs should be ordered pairs of solutions and dilution factors.
   The DF are multiplied by the concentrations of the
   respective solutions before they are combined.
   Redundant entries occuring within the input list
   are condensed by summing the weighted concentrations.

   Make a mixture of solution A and solution B by diluting
   solution A by 2X and solution B by 4X.
   Solution A is a 0.05 M solution of acetic acid and
   solution B is a 0.01 M solution of malic acid.
   A = mksol('Acetic',0.05);
```

```
B = mksol('Malic',0.01);
C = mixsol(A,0.5,B,0.25);
```

---

## MKADJ.

---

```
MKADJ Adjustment coefficients for dissociation constants.
   MKADJ(SOL) returns -log10(adjustment) for solution SOL
      assuming I=0, T=25C, and E=78.38.
   MKADJ(SOL,I) -log10 adjustments for ionic strength I
      assuming T = 25C and E=78.38.
   MKADJ(SOL,I,T) -log10 adjustments for I and T assuming.
      E=78.38
   MKADJ(SOL,I,T,E) adjusts additionally for dielectric
      constant E.
   MKADJ(SOL,I,T,E,p1,p2,...) sends additional inputs to the
      adjustment function.

   Note: The default adjustment function is Davies (with b=0.3).
   The user is free to specify their own function however via the
   PHTOOLSET function.  MKADJ requies that the adjust function
   be of the form F(z,I,T,p1,p2,...) where z is
```

---

## MKSOL.

---

```
MKSOL Make a solution of buffers.
   MKSOL('Buffer1',Conc1,'Buffer2',Conc2,...) makes a buffer
   by specifying nicknames and concentrations.

   Note: There is a special syntax allowed for a solution consisting
   of pure water.  S = MKSOL('water') returns a solution of water
   with a concentration of 55.556 Molar.  By default water is included
   in the solution for all solutions except those containing complex buffers
   which are assumed to already include the buffering due to water.

   Example:
      S1 = mksol('Malic',0.01,'Lactic',0.03);
      S1(1) %Access Malic acid information.
      S1(1).pKa %Access Malic acid pKa's.
```

## MULTISELECTDLG.

```
MULTISELECTDLG Select multiple items from list.
[Selection,OK]  = MULTISELECTDLG('String',S);  Select from a cell-array of
strings in S.  Selection is a cell array holding the final selection.  OK
will be 1 if the OK button was presses and will be 0 if either the CANCEL
button was pressed or if the figure was closed.

Example:

[A,B] = multiselectdlg('String',{'Apples','Pears','Cinnamon'});
```

## OPTIMADJ.

```
OPTIMADJ Optimize activity coefficients for a buffer solution.
   Activity coefficients depend on the ionic strength which
   in-turn depend on the degree of dissociation of acids and
   bases.  OPTIMADJ uses an iterative algorithm to optimize
   the adjustments due to activity coefficients.
   The output (ADJ) maybe used
   to update the dissociation constants by
      PKAadj = PKA - ADJ.adjpKa
   for pKa or
      PKBadj = PKB - ADJ.adjpKb
   for pKb.

   OPTIMADJ is vectorized to produce adjustments for vector
   valued inputs.

   ADJ = OPTIMADJ(SOL,pH) returns adjustment coefficients
   in struct ADJ.   adjpKa holds -log10(Ka adjustment) for each
   pKa.  adjpKb holds -log10(Kb adjustment) for each
   pKb.

   ADJ = OPTIMADJ(SOL,pH,T) determines adjustmnet based on
   celsius temperature T.

   ADJ = OPTIMADJ(SOL,pH,T,E) determines adjustment based on
   a solvent dielectric constant of E.
```

```
ADJ = OPTIMADJ(SOL,pH,T,E,I) determines adjustment based on
an ionic strength of I.  This cause optimadj to ignore the effects
on ionic strength from T and E.

ADJ = OPTIMADJ(SOL,pH,T,E,I,p1,p2,...) sends the additional
parameters to the adjustment function.

SOL = mksol('Acetic',0.03,'NaCl',0.3422);
pH = linspace(2,12,100)';
B1 = buffcapeval(SOL,pH);
ADJ = optimadj(SOL,pH,25);
B2 = buffcapeval(SOL,pH,[],ADJ);
plot(pH,B1,'r--');
hold on;
plot(pH,B2,'r-');
```

---

## PHTOOLSINFO.

---

```
PHTOOLSINFO Get information about the pHtools installation.
    Possible requests are
          home - The directory in which the toolbox is installed.
       version - The version of this installation.
          date - The copyright date of this installation.
        author - The people to whom the copyright belongs.
         state - The state of this release.
      lastpath - The path of the directory last visited.
         about - Information about pHtools and its history.
     defaultdb - The databases which come shipped with pHtools.
        userdb - The databases speciifed by the user (see USERDB).
      bufferdb - Both the user-created and default databases.

   Example:
      [H,V,D] = pHtoolsinfo('home','version','date');
```

---

## PRINTDB.

---

```
PRINTDB Print an inventory of one or more buffer databases.
   PRINTDB(DB,OUTFILE,FMT,FIELD1,FIELD2,...) DB is a cellstr of pathnames
   of databases, FMT is an output format and FIELD1, FIELD2,...
   are desired fields to be output.  By default all fields
```

```
   of a buffer are output.
   Acceptable values of FMT include 'ascii' and 'html'
   The HTML output format provides hyperlinks to the databases
   thereby allowing one to ``publish'' his or her database on the
   internet.

   When called without any input arguments this function uses a GUI.
```

---

## REPORTSHEET.

---

```
REPORTSHEET Interactive GUI for titration database.
   REPORTSHEET('FILE',FILENAME) uses data in FILENAME.
```

---

## RESETALLPOSITIONS.

---

```
Seems to be needed.  Add to figure ResizeFcn when
uipanel is used.
```

---

## SEARCHTEXT.

---

```
SEARCHTEXT(WORDS,STRUCT,FIELDNAME,FLAG);
Search a text field of a struct for a list of words.  Acceptable
flags are 'all','any','phrase'. The default flag is 'any'.
Spaces are used to delimit words in the list.  STRUCT may be
an array of structs.

   This is a utility function used by FINDBUFFER.
```

---

## SETLASTPATH_PHTOOLS.

---

```
SETLASTPATH_PHTOOLS Set the last path visited.

   SETLASTPATH_PHTOOLS(PATH) sets the last path visited to PATH.
```

## SPECIES.

```
SPECIES Determine concentrations of all species in solution.
   M = SPECIES(SOL,pH) returns a struct containing the
      concentrations at the specified pH.
      If [] is input for the pH, then the default pH range
      [2 12] is used.

      The output struct is organized in the folowing way.
      The main fields are the Nicknames of the buffers.
      Within each field is a struct containing the molar
      concentrations of each species. Negatively charged
      species are designated with an 'm' and positively
      charged species are designated with a 'p'.
      The value of the charge is appended to the field
      name.

   M = SPECIES(SOL,pH,ADJ) additionally use the
      adjustment coefficients in ADJ. If ADJ is [] then
      I=0, T=25, and E=78.38 are assumed (the default).

   Note: When called with no output argument, a Sillen diagram
   is produced (semi-log10 plot of concentrations vs. pH).
   In a Sillen diagram, the slope of the H+ curve is -1,
   the slope of the OH- curve is +1 and  the points of
   intersection between 2 species curves correspond with
   the Ka relating those 2 species.

   Example:
         %Generate Sillen diagram for a 12mM solution.
         %of malic acid using Davies equation adjustments.
         sol = mksol('Malic',0.012);
         mypH = [2:0.1:12]';
         ADJ = optimadj(sol,mypH);
         species(sol,mypH,ADJ);

   See also CB, BUFFCAPEVAL, IONICSTR, DCBDH, DCBDT, OPTIMADJ.
```

## SPECIES_NEW.

```
SPECIES Determine concentrations of all species in solution.
   M = SPECIES(SOL,pH) returns a struct containing the
      concentrations at the specified pH.
      If [] is input for the pH, then the default pH range
      [2 12] is used.

      The output struct is organized in the folowing way.
      The main fields are the Nicknames of the buffers.
      Within each field is a struct containing the molar
      concentrations of each species. Negatively charged
      species are designated with an 'm' and positively
      charged species are designated with a 'p'.
      The value of the charge is appended to the field
      name.

   M = SPECIES(SOL,pH,ADJ) additionally use the
      adjustment coefficients in ADJ. If ADJ is [] then
      I=0, T=25, and E=78.38 are assumed (the default).

   Note: When called with no output argument, a Sillen diagram
   is produced (semi-log10 plot of concentrations vs. pH).
   In a Sillen diagram, the slope of the H+ curve is -1,
   the slope of the OH- curve is +1 and  the points of
   intersection between 2 species curves correspond with
   the Ka relating those 2 species.

   Example:
         %Generate Sillen diagram for a 12mM solution.
         %of malic acid using Davies equation adjustments.
         sol = mksol('Malic',0.012);
         mypH = [2:0.1:12]';
         ADJ = optimadj(sol,mypH);
         species(sol,mypH,ADJ);

   See also CB, BUFFCAPEVAL, IONICSTR, DCBDH, DCBDT, OPTIMADJ.
```

## SPREADSTRUCT.

```
SPREADSTRUCT Create spreadsheet-like struct from vector and cell-array data.
```

```
Example:
Suppose you have data in a text file called mydata.txt like
Sarah 35 105.5 Female
John  43 210.8 Male
Mack  37 189.3 Male

Use TEXTREAD to read-in data.
[N,A,W,S] = textread('mydata.txt','%s %d %f %s');

Then use SPREADSTRUCT to place data in a spreadsheet-like
struct (i.e. one observation per record)

S = spreadstruct('Name',N,'Age',A,'Weight',W,'Sex',S);
Now to access each subjects data use the following syntax

S(1)

or to see data for a particular variable use

S(1).Weight

If desired, the original data vectors can be recovered at any time
by concatenation as in the following.

N = [S.Name]';
A = [S.Age]';

or equivalently

N = cat(1,S.Name);
A = cat(1,S.Age);

Statistics on the data can be computed easily as in

MeanAge = mean([S.Age]);
VarAge = var([S.Age]);
```

---

## STRUCTCAT.

---

```
STRUCTCAT Concatenate 2 struct arrays using only their common fields.
   STRUCTCAT(A,B)
   STRUCTCAT(A,B,'intersect') concatenates by eliminating non-common
   fields among the structs.
   STRUCTCAT(A,B,'union') concatenates by using the union of
```

```
fields among the structs.  Empty values are used to fill in
missing values in structs.
```

---

## TARGETCONC.

---

```
TARGETCONC(sol1,sol2,target_acid,target_species,target_conc,Xo,target_pH,varargin);
        Determine how much of sol2 to add to sol1 in order that the
           requested target_species of the requested target_acid component
       is equal to target_conc after adjusting the final pH to target_pH.
       Xo is used as the initial guess for the amount of sol2 needed to be
       added to sol1.

TARGETCONC(sol1,sol2,target_acid,target_species,target_conc,Xo,target_pH,varargin,flag,T,E,
will adjust for temperature T and dielectric E if flag='adjust' but will not adjust for
other values of flag.  Providing STOP_TOL will cause the algorithm to use the convergence
tolerance of STOP_TOL.  The default is 1.e-4. P1,P2,etc are parameters that
are passed to the objective function.  The default objective function ignores
these.

[fac,bounds] = TARGETCONC(...) returns in fac the dilution factor of sol2 needed
   to obtain the target species concentration and bounds is an upper and lower
   bound on that dilution factor considering a pH meter error of +/- 0.01 pH units.

   Users are encouraged to look at bounds when using this estimator in application
   or production settings where system variability is important.

   Example: Suppose we are given a 0.01 M solution of Lactic acid.  How much
   Malic acid would we need to add in order that the 2- anion of Malic acid
   has a concentration of 0.001 given that we will adjust the final pH
   (adjusted with say HCL or NaOH) to 4.0?

   Sgiven = mksol('Lactic',0.01);
   Sadd = mksol('Malic',1);
   target_acid = 'Malic';
   target_species = 'm2';
   target_conc = 0.001;
   init_guess = 1;
   target_pH = 4.0;
   [fac,sens] = targetconc(Sgiven,Sadd,target_acid,target_species,target_conc,init_guess,ta

   %Let's check the answer
   S = mksol('Lactic',0.01,'Malic',fac);
   V = species(S,4.0); V.Malic
```

```
   Err = abs(V.Malic.m2-target_conc)

   disp(['Thus, we should prepare a solution whose total malic acid concentration is ',num2
```

---

## TARGETCONC_OBJ.

---

```
TARGETCONC_OBJ(fact,sol1,sol2,target_conc,target_pH,flag,T,E,varargin)
   Objective function used internally by TARGETCONC.

   See also TARGETCONC
```

---

## TARGETPH.

---

```
TARGETPH Determine the concentration fraction of a second buffer solution
   required to modify the pH to a desired target value.

   F = TARGETPH(SOL1,SOL2,GUESS,pH) uses an initial guess to start a search
   to find the relative concentration of SOL2 required in SOL1 to give
   the target pH.  Algorithm works best when a 2 element vector is given
   for GUESS representing upper and lower bounds for the search.
   FAC can be thought of as the percentage of the concentration of SOL2
   required.

   NOTE!! Typically the output should be multiplied by the volume in liters
   of the actual volume in question to get the volume of SOL2 needed to be
   added.

   F = TARGETPH(SOL1,SOL2,GUESS,pH,'adjust') uses ionic strength adjustments.

   F = TARGETPH(SOL1,SOL2,GUESS,pH,'adjust',T) also adjusts for temperature T.
   TARGETPH(SOL1,SOL2,GUESS,pH,'adjust',T,E) also adjusts for dielectric
   constant E.

   F = TARGETPH(SOL1,SOL2,GUESS,pH,'adjust',T,STOP_TOL) uses the convergence
   tolerance STOP_TOL (DEFAULT = 1.E-4) for determining the mixture fraction F.


   Note:

   [F,B] = TARGETPH(...) additionally returns in B the concentration
```

```
fractions required at -0.01 and 0.01 pH units of the target pH.
This is recommended when working with weak acids and bases in in
solutions with little buffer capacity.  While the pH predictions
are usually quite accurate the concentration fractions may be
highly senstitive to small perturbations in the target pH.


Example:
    %One where we know the correct answer.
    sol_end = mksol('Malic',0.03,'Lactic',0.04);
    target = initpH(sol_end)
    sol_start = mksol('Malic',0.03);
    sol_add = mksol('Lactic',0.001); %Titrating with 1mM lactic acid.
    amount = targetpH(sol_start,sol_add,[1,50],target)

     %7mM benzoic acid is added to a cucumber slurry.
    %How much acetic acid is needed to lower the pH to 3.5?
     sol_start = mksol('CJU1',1,'Benzoic',0.007);
    sol_add = mksol('Acetic',0.001); %Consider 1mM amounts of acetic acid.
     amount = targetpH(sol_start,sol_add,1,3.7);
```

---

## TARGETPH_MC.

---

```
TARGETPH_MC Determine the concentration fraction of a second buffer solution
   required to modify the pH to a desired target value using a Monte-Carlo
   approach.  The output is a N-by-1 vector of estimates.

   F = TARGETPH_MC(N,SOL1,SOL2,GUESS,pH) uses an initial guess to start a search
   to find the relative concentration of SOL2 required in SOL1 to give
   the target pH.  Algorithm works best when a 2 element vector is given
   for GUESS representing upper and lower bounds for the search.
   FAC can be thought of as the percentage of the concentration of SOL2
   required.

   NOTE!! Typically the output should be multiplied by the volume in liters
   of the actual volume in question to get the volume of SOL2 needed to be
   added.

   F = TARGETPH_MC(N,SOL1,SOL2,GUESS,pH,'adjust') uses ionic strength adjustments.

   F = TARGETPH_MC(N,SOL1,SOL2,GUESS,pH,'adjust',T) also adjusts for temperature T.
   TARGETPH_MC(N,SOL1,SOL2,GUESS,pH,'adjust',T,E) also adjusts for dielectric
   constant E.
```

```
F = TARGETPH_MC(N,SOL1,SOL2,GUESS,pH,'adjust',T,STOP_TOL) uses the convergence
tolerance STOP_TOL (DEFAULT = 1.E-4) for determining the mixture fraction F.


Note:

[F,B] = TARGETPH_MC(...) additionally returns in B the concentration
fractions required at -0.01 and 0.01 pH units of the target pH.
This is recommended when working with weak acids and bases in in
solutions with little buffer capacity.  While the pH predictions
are usually quite accurate the concentration fractions may be
highly senstitive to small perturbations in the target pH.


Example:
    %One where we know the correct answer.
    sol_end = mksol('Malic',0.03,'Lactic',0.04);
    target = initpH(sol_end)
    sol_start = mksol('Malic',0.03);
    sol_add = mksol('Lactic',0.001); %Titrating with 1mM lactic acid.
    amount = targetpH_MC(100,sol_start,sol_add,[1,50],target)

    boxplot(amount);

     %7mM benzoic acid is added to a cucumber slurry.
    %How much acetic acid is needed to lower the pH to 3.5?
     sol_start = mksol('CJU1',1,'Benzoic',0.007);
    sol_add = mksol('Acetic',0.001); %Consider 1mM amounts of acetic acid.
     amount = targetpH(sol_start,sol_add,1,3.7);
```

---

## TARGETPHOBJ.

---

```
TARGETPHOBJ Objective function for targetpH.
   TARGETPHOBJ(fact,sol1,sol2,target_pH,flag,T,E,varargin)
   returns the Cb at the target pH for the mixture
   solution of concentration conc.
```

---

## TARGETSOL.

---

```
TARGETSOL Determine amounts to add to acheive targets in a solution.
   TARGETSOL is based on the idea that you want to use some stock solutions
   to achieve a desired ionic strength and species concentration.  All
   results are prepared on a 1L total volume basis.  If your stocks
   are too dilute to provide the necessary targets at volumes less than
   1L a warning message will be displayed.

   Simply type the word 'targetsol' at the MATLAB prompt to run
   TARGETSOL interactively. At the end (hopefully convergence
   has happened) then a little recipe is printed out - or you can
   save to a text file (your option).

   You will be asked to create your stock solutions which may include
   acidulents and electrolytes.

   The program attempts to determine "parts" of each solution required
   to meet the desired targets.

   Note on pH:  The pH you enter is assumed **fixed**.  What this means
   is that the program asssumes in all calculations that NaOH or HCl are
   being added to bring the pH of the final solution to the set pH.  The
   increase in ionic strength due to adding this NaOH or HCl **is** taken
   into account.

   Convergence issues:  Check the intermediate output that is printed
   during the optimization phase to see that the "Tracked species conc"
   and "Ionic Strength" are converging to the values you wanted.
   Obviously some combinations of pH, ionic strength and species
   concentrations are not possible given your stocks.  For example
   if your target species is not in any of your stocks the desired
   results are impossible or if the concentration in the Base stock
   is greater than in the acidulent stock then there may be problems
   and so on...

   TARGETSOL can also be run in batch mode.  Batch mode requires that
   the user enter in a cell array of stock solutions as created by MKSOL
   as well as a struct specifying linear constraints the target ionic
   strength, the set temperature, and the set pH.  Note that temperature and
   pH are always assumed given and constant. Finally, a text file name
   must be provided as the destination of the ouput.

   Example (Batch mode):
      StockSol = {mksol('Acetic',0.01),mksol('Lactic',0.01),mksol('NaCl',0.5)};
      C = [1 1 0 0 0 0 0 0
            0 0 0 0 0 0 0 0
            0 0 0 0 0 0 0 0
```

```
         0 0 0 0 0 0 0 0
         0 0 0 0 0 0 0 0
         0 0 0 0 0 0 0 0
         0 0 0 0 0 0 0 0
         0 0 0 0 0 0 0 0]
   V = [0.001 nan nan nan nan nan nan nan]';
   L = {'Acetic:m0','Acetic:m1','Lactic:m0','Lactic:m1','NaCl:lytes','water:m1','water:m

   Constraint = struct('Constraint',C,'Vals',V,'Labels',L);
   TargetIonicStr = 0.342;
   SetTemp = 25;
   SetpH = 2.5;
   Outfile = 'myresults.txt';

   targetsol(StockSol,Constraints,TargetIonicStr,SetTemp,SetpH,Outfile)


See also TARGETSOL_CNSTRNTDLG
```

## TARGETSOL_CNSTRNTDLG.

```
TARGETSOL_CNSTRNTDLG Matrix-based linear constraint dialog for TARGETSOL.
   CONSTRAINT = TARGETSOL_CNSTRNTDLG(Sol1,Sol2,...) creates a linear constraint
   dialog for the components of the solutions Sol1,Sol2,...

   See also TARGETSOL
```

## TARGETSOL_GUI.

```
TARGETSOL_GUI Determine amounts to add to acheive targets in a solution.
   TARGETSOL_GUI is based on the idea that you want to use some stock solutions
   to achieve a desired ionic strength and species concentration.  All
   results are prepared on a 1L total volume basis.  If your stocks
   are too dilute to provide the necessary targets at volumes less than
   1L a warning message will be displayed.

   Simply type the word 'targetsol_gui' at the MATLAB prompt to run
   TARGETSOL_GUI interactively. At the end (hopefully convergence
   has happened) then a little recipe is printed out - or you can
   save to a text file (your option).
```

## TARGETSOL_OBJ.

```
TARGETSOL_OBJ Objective function for TARGETSOL.
```

## TEST.

## TESTDATA.

```
Lets simulate a titration:
```

## UPDATE_OLD_DB.

```
UPDATE_OLD_DB Script that updates databases from previous pHtools versions.
   UPDATE_OLD_DB calls pHtoolsinfo('bufferdb') to determine which
   buffer databases to work on. Then all databases found are updated to
   the current format.
```

## USERDB.

```
USERDB Graphical interface for managing user databases.
   This interface allows you to modify which buffer databases
   pHtools software is aware of.  Only existing mat-file
   databases are allowed.

   Note: To create new databases use EDITBUFFER.
```

See also EDITBUFFER.

---

More Examples

# Simple Examples

EXAMPLE 3

What is the pH of a 0.01 M solution of NaOH?

---

In dilute aqueous solutions the ion-product constant is $\left[W^-\right]\left[H^+\right] = 1 \times 10^{-14}$. Since NaOH is a strong base we will assume that its dissociation is complete. We can ignore the contribution to $\left[W^-\right]$ from water alone ($1 \times 10^{-7}$M) and let $\left[W^-\right] = 0.01$.

$$\left[H^+\right] = \frac{1 \times 10^{-14}\text{M}}{0.01\text{M}} \quad (43)$$

$$= 1 \times 10^{-12}\text{M} \quad (44)$$

Therefore, the pH of a 0.01M solution of NaOH is approximately 12.

---

Using pHTools ...

```
S = mksol('NaOH',0.01);
ANS = initpH(S)
```

EXAMPLE 4

What is the initial pH of 0.01M solution of acetic acid?

---

The $pK_a$ of acetic acid is 4.75 which means that the $K_a$ is

$$1.778 \times 10^{-5} = \frac{\left[A^{-1}\right]\left[\mathrm{H}^{+}\right]}{[HA]} \tag{45}$$

$$\tag{46}$$

Also, the total concentration of acetic acid (C) is equal to the sum $[HA]$ and $[A^{-}]$ which must be 0.01. If we assume that the contribution to $\left[\mathrm{H}^{+}\right]$ from water is negligible then we can write

$$1.778 \times 10^{-5} = \frac{\left[\mathrm{H}^{+}\right]\left[\mathrm{H}^{+}\right]}{[C] - \left[\mathrm{H}^{+}\right]} \tag{47}$$

$$\tag{48}$$

To solve for $\left[\mathrm{H}^{+}\right]$ we need to use the quadratic formula and write

$$\left[\mathrm{H}^{+}\right] = \frac{\mathrm{K}_a + \sqrt{\mathrm{K}_a{}^2 + 4(\mathrm{K}_a)(C)}}{2} \tag{49}$$

$$= \frac{1.778 \times 10^{-5} + \sqrt{3.161 \times 10^{-10} + 7.112 \times 10^{-7}}}{2} \tag{50}$$

$$= 4.307 \times 10^{-4} \tag{51}$$

Therefore, we estimate the initial pH as $-\log_{10}(4.307 \times 10^{-4}) = 3.37$.

---

Using pHTools ...

S = mksol('Acetic',0.01);
ANS = initpH(S)

You should be aware of the following trends in the above examples. Many pH calculations proceed by ignoring the contributions of ions from water. This may not always be an acceptable approximation and consideration should be give to the dissociation constants of the buffers in question as well as there concentration. For problems with strong acids and bases the solution of a first order equation is usually all that is required. For problems concerning a weak acid or base the with a single dissociable group, the quadratic formula is required. For other problems the use of an iterative procedure such as the *method of*

*successive approximations* is required.

EXAMPLE 5

pH meters must be calibrated with standards of known pH. A particular recipe for a pH standard calls for adding 50 mL of 0.1 M potassium hydrogen phthalate and 22.3 mL of 0.1 M HCl to a flask and bringing the total volume to 100 mL $H_2O$. What pH is the standard in the flask supposed to represent?

---

```
S = mksol('KHP',50*0.0001/0.1,'HCl',22.3*0.0001/0.1);
p1 = initpH(S,[],'adjust')
```

# Generating Experimental Designs

You want to study the effect of organic anion concentration and pH on the growth rate of your favorite bacterium. Design a $5^2$ factorial experiment in which pH and *protonated lactic acid* concentrations are varied. Consider the 5 protonated lactic acid levels 0,7.5,15.0, 22.5, and 30.0 mM and the 5 pH levels 3.5, 4.25,5.00,5.75, and 6.50. In a factorial arrangement, where all possible combinations of these levels are considered, this gives 25 treatment combinations. To make results comparable you decide you want to fix the ionic strength at 0.342. Determine the amounts of total lactic acid and NaCl to add to obtain each of the desired treatment combinations.

Remember that most pHTools functions require pH as an input. We will use this to our advantage and just concentrate on the ionic strength and lactate issues. In other words, we will just fix the pH at the desired level and determine the necessary NaCl and lactic acid amounts assuming that the pH can be obtained by adjusting the final pH with sufficient NaOH or HCl.

Here is a driver program (gendesign.m) which determines the required amounts. It involves an iterative minimization approach. The goal is to minimize a penalty function which measures the discrepancy between our desired lactate and ionic strength levels and those predicted given the most recent suggested levels of total lactic acid and NaCl. The m-file designobj.m is the function which calculates the penalty to be minimized. The outputs are stored in a 5x5 cell array.

---

```
%Consider the pH fixed at desired level and then determine
%salt and total lactic amounts to add.  You will need to
%add NaOH or HCl to obtain the pH experimentally.

pH = linspace(3.5,6.5,5)'; %Consider these fixed and known.

%Desired concentration of anion (not total).
```

```
desired_lactate = linspace(0,0.03,5);

%This is the ionic strength we are shooting for.
desired_ionic = 0.342;

%Use these initial guesses in the optimization process.
init_salt = 0.342;
init_lactic = 0.015;
init_guess = [init_lactic init_salt];

use = cell(length(pH),length(lactate));

for i = 1:length(pH)
        for j = 1:length(lactate)
                opts = seqsimpset('Display','iter');
                use{i,j} = seqsimp('design_obj',init_guess,...
           opts,pH(i),...
                           'U_TSG_ex0702','Lactic',...
   'NaCl',desired_ionic,...
   desired_lactate(j));
        end
end
```

---

Here is the designobj.m which produces the penalty to be minimized.

---

```
function Penalty = design_obj(V,pH,buffer,acid_sol,salt_sol,...
desired_ionic,desired_lactate)

S = mksol(buffer,1,acid_sol,V(1),salt_sol,V(2));



ADJ = optimadj(S,pH);
I = ionicstr(S,pH,[],ADJ); %Get the estimated ionic
                           %strength of the solution.

%Now we need to get the concentration of just the anion.
index = find(strcmp({S.Nickname},'Lactic'));
conc = Cb(S,pH,'partial',ADJ);
Lactate = conc(1,index);

%This is the Euclidean norm of the difference.
Penalty = sqrt((desired_ionic-I)^2 + (desired_lactate-Lactate)^2);
```

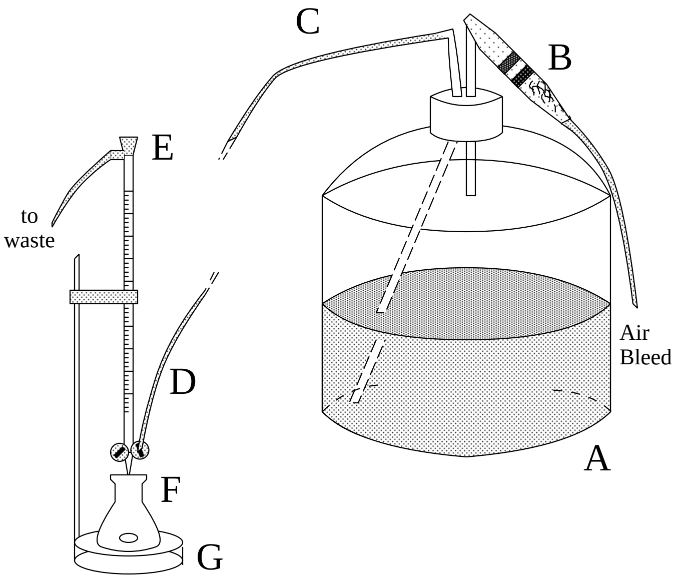


Figure 17: A)Reservoir containing sodium hydroxide stock B) $CO_2$ trap. Polyethylene drying tube is first packed with about 3 cm of cotton followed by about 3 cm Drierite$^{TM}$then 3 cm of Ascarite$^{TM}$followed by 3 cm of Drierite and finally more cotton. C) Reservoir outlet to volumetric pipet D) From reservoir outlet connecting to stopcock 1 of volumetric pipet. E) Double stopcock volumetric pipet. F) Flask containing magnetic stir bar and buffer to be titrated. G) Magnetic stir plate. H) Ring stand

---

Setting Up a Sodium Hydroxide Reservoir

- For most applications (microbiological media, fruit juices etc.) a 100mM stock of sodium hydroxide is convenient.
- The sodium hydroxide stock drawn from the reservoir should be calibrated often against a standard (see Appendix ).

Preparation of Standardized Sodium Hydroxide and Hydrochloric Acid Stocks

## Preparation of NaOH Stock

1. Boil 1 L of $dH_2O$ for 5 minutes to remove $CO_2$ (Keep flask covered with foil to prevent evaporative loss). Transfer water to reservoir while still warm.

2. Pipet approximately 7mL of 50% NaOH solution to the reservoir and mix thoroughly.

## Calibration of NaOH Stock

The following method is designed to calibrate a sodium hydroxide stock that is made to be approximately 100mM (0.1N).

Materials:

- Uncalibrated sodium hydroxide stock
- Potassium acid phthalate (m.w. 204.22)
- 100 mL Erlenmeyer flask
- Magnetic stir bar and stir plate
- Phenolphthalein indicator
- Volumetric pipet

Directions:

1. Weigh out approximately 0.5g of of dessicated potassium acid phthalate. Record the weight as W.
2. Into the Erlenmeyer flask put the potassium acid phthalate and dissolve with 20 mL of $dH_2O$. Note, not all crystals may dissolve.
3. Add 2-3 drops of phenolphthalein indicator.
4. Wash the volumetric pipet with several volumes of sodium hydroxide stock.
5. Fill volumetric pipet and record initial volume (in liters) as V1.
6. While stirring at a slow rate, titrate the potassium acid phthalate solution to the phenolphthalein end-point.
7. Record the volume (in liters) left in the pipet as V2.

The concentration of the sodium hydroxide stock is given by

$$C = \frac{W}{204.22(V2 - V1)} \tag{52}$$

## Calibration of HCL Stock

Materials:

- Calibrated NaOH stock (yN).
- Uncalibrated HCl stock.
- 100 mL Erlenmeyer flask
- Magnetic stir bar and stir plate
- Phenolphthalein indicator
- Volumetric pipet

Directions:

1. Add approximately 20mL of HCl stock into the Erlenmeyer flask.
2. Add 2-3 drops of phenolphthalein indicator.
3. Wash the volumetric pipet with several volumes of sodium hydroxide stock.
4. Fill volumetric pipet and record initial volume (in liters) as V1.
5. While stirring at a slow rate, titrate the HCl solution to the phenolphthalein end-point.
6. Record the volume (in liters) left in the pipet as V2.

The concentration of the HCl stock is given by

$$C = y\frac{(V2 - V1)}{20mL} \tag{53}$$

Proof of Computational Formula for $C_b$ and $\beta$

**Theorem 1.** *The partial contribution of a single n-protic acid having molar concentration $C$ in a solution containing $C_b$ moles of base is*

$$\frac{C\sum_{i=1}^{n}\left[(n+1-i)\left[H^{+}\right]^{i-1}\prod_{j=1}^{n+1-i}K_{aj}\right]}{\sum_{i=1}^{n+1}\left[\left[H^{+}\right]^{i-1}\prod_{j=1}^{n+1-i}K_{aj}\right]} \tag{54}$$

*Proof of 12.* Proof is by induction. For clarity we will define $X_n^{j-}$ to be a molecule having $j$ dissociated groups out of a total of $n$ and $C_k$ to equal $\sum_{p=1}^{k}[X_k^p]$. We note that for a monoprotic weak acid ($n = 1$), in a solution

containing strong acid at a concentration of $C_a$ the equilibrium and conservation equations

$$K_{a1} = \frac{\left[\mathrm{H}^+\right] X_1^{1-}}{X} \tag{55}$$

$$C_k = [X_1] + \left[X_1^{1-}\right] \tag{56}$$

$$K_w = \left[\mathrm{H}^+\right] \left[OH^-\right] \tag{57}$$

along with the the charge balance

$$C_b = \left[X_1^{1-}\right] + \left[OH^-\right] + C_a - \left[\mathrm{H}^+\right] \tag{58}$$

$$\tag{59}$$

give

$$C_b = \frac{C_1 K_{a1}}{\left[\mathrm{H}^+\right] + K_{a1}} + \frac{K_w}{\left[\mathrm{H}^+\right]} + C_a - \left[\mathrm{H}^+\right] \tag{60}$$

Here $K_w / \left[\mathrm{H}^+\right] - \left[\mathrm{H}^+\right]$ is the partial contribution due to the dissociation of water and $C_a$ is the partial contribution due to strong acid (Butler and Cogley, 1998, pp.130-134). Therefore, the partial contribution of the weak acid is

$$C_b = \frac{C_1 K_{a1}}{\left[\mathrm{H}^+\right]} \tag{61}$$

which is 12 evaluated at $n = 1$. Now, assuming that ( 12) is true for $n = m$, which gives

$$\frac{C_m \sum_{i=1}^{k} \left[(m+1-i) \prod_{j=1}^{m+1-i} (Kaj) \left[\mathrm{H}^+\right]^{i-1}\right]}{\sum_{i=1}^{m+1} \left[\left[\mathrm{H}^+\right]^{i-1} \prod_{j=1}^{m+1-i} K_{aj}\right]} \tag{62}$$

we must prove that ( 12) is true when $n = m+1$. First note that if $n = m+1$ it must actually be that

$$C_m = C_{(m+1)} - \left[X_{m+1}^{(m+1)-}\right] \tag{63}$$

and that

$$K_{a(m+1)} = \frac{\left[\mathrm{H}^+\right] \left[X_{(m+1)}^{(m+1)-}\right]}{\left[X_{(m+1)}^{m-}\right]} \tag{64}$$

$$= \frac{\left[\mathrm{H}^+\right] \left[X_{(m+1)}^{(m+1)-}\right]}{C_b} \tag{65}$$

$$= \frac{\left[\mathrm{H}^+\right] \left[X_{(m+1)}^{(m+1)-}\right]}{\frac{C_{(m+1)} \sum_{i=1}^{m+1} \left[(m+2-i) \prod_{j=1}^{m+2-i} (Kaj) \left[\mathrm{H}^+\right]^{i-1}\right]}{\sum_{i=1}^{m+1+1} \left[\left[\mathrm{H}^+\right]^{i-1} \prod_{j=1}^{m+1+1-i} K_{aj}\right]}} \tag{66}$$

Solving now for $\left[X_{(m+1)}^{(m+1)-}\right]$ we get,

$$\left[X_{(m+1)}^{(k+1)-}\right] = \frac{C_{(m+1)} \prod_{j=1}^{m+2}(K_{aj})}{\sum_{i=1}^{m+2+1}\left[(i-1)\prod_{j=1}^{m+1-i}(Kaj)\left[\mathrm{H}^{+}\right]^{i-1}\right]} \tag{67}$$

which allows us to write the corrected charge balance contribution as

$$\left[X_{m+1}^{(m+1)-}\right] + (m+2)\left[X_{(m+1)}^{(m+1)-}\right] \tag{68}$$

$$= \frac{\left[\mathrm{H}^{+}\right] C_b}{K_a(m+1)} + (m+2)\left[X_{(m+1)}^{(m+1)-}\right] \tag{69}$$

$$= \frac{C_{m+1}\sum_{i=1}^{m+1}\left[(m+2-i)\prod_{j=1}^{m+2-i}(Kaj)\left[\mathrm{H}^{+}\right]^{i-1}\right]}{\sum_{i=1}^{m+2}\left[(i-i)\prod_{j=1}^{m+2-i}(Kaj)\left[\mathrm{H}^{+}\right]^{i-1}\right]} \tag{70}$$

which is ( 12) evaluated at n = m+1 completing the proof. □

Statistical Significance Versus Biological Significance

The purpose of this section is to address some caveats regarding the tests of significance of buffer components. The F-tests for buffer components are based on a comparison of variability in the titration data explained by a model with the component included versus one in which the component is not. If the titration data is very noisy then a component which contributes to the buffering will not be detected. In cases such as this, **when the null hypothesis of no difference is not rejected**, one should consider the power of the test. Powers less than 0.8 are considered poor.

Alternatively if the precision of the pH measurements and HPLC measurements is very good then even very small contributions can be significant. In such cases, **when the null hypothesis of no difference is rejected**, it is important to consider if the buffering due to the component is biologically significant or meaningful.

Lastly, it should be understood that even when the power of the test is greater than 0.8 and the null hypothesis of no difference is not rejected the buffer component may be biologically significant. Low concentrations of detergents would be a good example.

# Glossary

## A

**acid** The Brønsted-Lowry definition of an acid is a molecule which can donate a proton., p. 10.

## B

**base** The Bronsted-Lowry definition of an acid is a molecule which can accept a proton., p. 8.

**buffer capacity** Defined by Van Slyke in 1922 as $\beta = \frac{\partial Cb}{\partial pH}$. Buffer capacity, unlike the end-point of a titration, does depend on the volume of the solution being titrated., p. 5.

## E

**end-point** The point in a titration at which some indicator of equivalance is first noticed. The most common end-point indicators are dyes that change color around the equivalence point(e.g. phenolphthalene, bromocresol red)., p. 71.

## F

**F-test** A statistical hypothesis test concerning the ratio of two variances. The ratio of two variances has an F-distribution., p. 30.

## G

**Generalized Titration** A titration in which the moles of base added per liter does not equal the moles of titrant added per liter. This typically occurs when the titrant is a weak acid or base., p. 25.

## H

**Hat matrix** The projection matrix (or pseudo-projection) matrix which maps the observables into the prediction space. For standard linear least-squares, the Hat matrix is $X(X'X)^{-1}X'$., p. 29.

## I

**ionic strength** The quantitity $I = \sum_{i=1}^{n} c_i z_i^2$ is typically called the ionic strength. This is actually a simplification of the form derived from Debye-Hückle theory which holds for dilute solutions of ions., p. 5.

## L

**local polynomial regression** Weighted polynomial regression where the weights are non-zero only in some local region about the desired prediction point. Typically weights are assigned using a Kernel function of the distance between the ., p. 5.

## M

**mat-file** A binary file with a .mat extension. Mat-files are structured to efficiently store all MATLAB variables., p. 34.

## P

**partial buffer component** When several compoounds are present in a solution their contriubtions to the overall buffer capacity is additive. The partial buffer component is just the part of such a sum corresponding to a particular compound., p. 8.

## S

**salt** The electrically neutral product of the reaction between an acid and a base. Some salts are highly soluable in water while others may precipitate out of aqueous solution., p. 35.

## T

**titrant** The buffer (typically a strong acid or base) being used to titrate a buffer solution., p. 25.

**titration** An experimentally determined relationship between the moles of base (Cb) added per liter of a buffer solution and the pH of the buffer solution., p. 5.

## Z

**zwitterion** A molecule containing both an acidic and a basic functional group., p. 36.

# References

Butler, J. N. and D. R. Cogley, 1998. Ionic Equilibrium: Solubility and pH Calculations. John Wiley and Sons.

Cleveland, W. S., S. J. Devlin, and E. Grosse, 1988. Regression by local fitting: Methods, properties, and computational algorithms. J. Econometrics. 37:87–114.

Fan, J. and I. Gijbels, 1996. Local polynomial modelling and its applications. Chapman and Hall.

Samson, E., G. Lemaire, J. Marchand, and J. Beaudoin, 1999. Modeling chemical activity effects in strong ionic solutions. Comp. Mater. Sci. 15:285–294.

Van Slyke, D., 1922. On the measurement of buffer values and on the relationship of buffer value to the dissociation constant of the buffer and the concentration and reaction of the buffer solution. Journal of the American Chemical Society 52:525–570.
